# Temporal dynamics of premenstrual dysphoric disorder revealed through predictive symptom networks

**DOI:** 10.1038/s41467-026-78590-x

**Published:** 2026-11-10

**Authors:** Ella Schleimann-Jensen, Inger Sundström-Poromaa, Johanna M. Hoppe, Sinha Engel, Jeff Kiesner, Erika Comasco

**Affiliations:** 1 Department of Women’s and Children’s Health, Uppsala University, Uppsala, Sweden; 2 Department of Psychology, Uppsala University, Uppsala, Sweden; 3 Department of Psychology, Friedrich-Alexander-Universität Erlangen-Nürnberg, Erlangen, Germany; 4 Department of Psychology (DPSS) and Padova Neuroscience Center, University of Padova, Padova, Italy; 5 Science for Life Laboratory (SciLifeLab), Uppsala University, Uppsala, Sweden

**Keywords:** Computational neuroscience, Psychiatric disorders, Reproductive signs and symptoms

## Abstract

Temporal dynamics of psychiatric symptoms challenge researchers and clinicians due to their inherent unpredictability. The cyclical on–off nature of symptoms linked to physiological rhythms, such as the menstrual cycle, provides a unique window to apply temporal and dynamic modeling of psychiatric symptoms. This can be studied in premenstrual dysphoric disorder, a female hormone-triggered condition characterized by cyclical symptoms. Yet intra-individual temporal patterns and dynamics of these symptoms remain unknown. Here we show that individuals with premenstrual dysphoric disorder differ widely in symptom timing, severity, and persistence, clustering into distinct temporal symptom profiles. Dynamic modelling uncovers temporal order among core psychiatric symptoms, with anxiety emerging as a central symptom in the unfolding of affective dysregulation. Depression shows the strongest predictive associations with subsequent severity of the other core symptoms, while irritability predicts the severity of depression at the longest time-interval. These temporal symptom dynamics reveal mechanistic psychoneuroendocrine pathways and inform precision, time-sensitive interventions for the mental health of individuals with premenstrual dysphoric disorder.

## Abstract (web summary)

Premenstrual dysphoric disorder causes cyclical mental symptoms, but how these symptoms unfold over time is not yet understood. This study reveals temporal patterns and identifies anxiety as a promising treatment target.

## Introduction

The temporal dynamics of psychiatric symptoms represent a challenge to researchers and clinicians due to their inherent unpredictability. The cyclical “on–off” nature of symptoms linked to physiological rhythms such as the menstrual cycle, however, provides a unique window to apply temporal and dynamic modelling of psychiatric symptoms^1^. By integrating psychoneuroendocrinology with computational psychiatry approaches, our mechanistic understanding of psychiatric symptom generation and relief can be advanced to inform precision, and time-sensitive approaches to mental health care.

Menstruation, experienced daily by over 300 million individuals worldwide, remains stigmatized despite being a physiological process regulated by functional hormonal changes that are beneficial for maintaining individuals’ reproductive function and health^2^. Notably, for some, the menstrual cycle imposes a wave of emotional and physical challenges^3,4^, which are still often dismissed by care professionals^5,6^. Around 3-8% of people who menstruate experience affective, cognitive, and physical symptoms of an intensity that severely impairs daily functioning and creates distress, meeting the diagnostic criteria for premenstrual dysphoric disorder (PMDD)^7,8^. Given the accumulated years of a woman’s reproductive life, PMDD imposes substantial burden, with an estimated loss of 3000 healthy days of life ( > eight years) in affected individuals^9,10^, including suicidal thoughts^11^. This burden has a typical cyclical pattern – commonly described as symptoms emerging during the premenstrual phase, and remitting after the onset of menses^7^.

The cyclical nature of PMDD suggests a role of sex hormones in central nervous system regulation, further supported by the observation that symptoms remit in non-ovulatory cycles, after ovariectomy, or with drugs inducing ovulation suppression^12,13^. Additionally, the hormonal changes that trigger the transition from the asymptomatic to the symptomatic state, affect not only cortico-limbic brain functions^14^, but also a variety of physiological systems, such as the autonomic nervous system, circadian rhythm, the gastrointestinal system, and the immune system^15–17^. Given that PMDD symptomatology is highly dynamic and that the menstrual cycle itself involves changes in many physiological systems across the body that could influence psychological symptoms and mental states^18^, our understanding of PMDD must be further advanced. This can be achieved by recognizing the heterogeneity of symptom changes across individuals, by identifying subtypes of patients and symptoms, and by establishing the dynamic temporal associations among symptom changes that can be associated with distinct physiological systems and neural circuitries.

To date, PMDD symptomatology and its temporal onset and offset have generally been treated as homogeneous across, and stable within, individuals, with little attention given to inter- and intra-individual heterogeneity. Kiesner and colleagues^19–23^ have examined inter- and intra-individual differences in physical and psychological changes across the menstrual cycle, although this research focused solely on the general population without considering PMDD. However, evidence limited to total PMDD symptoms, anxiety, sudden sadness, and anger/irritability has pointed to temporal subtypes among patients with PMDD^24^. Emerging theoretical work has further highlighted the potential relevance of heterogeneity in menstrual cycle-related affective sensitivity^25^. Notably, the intra-individual dynamic manifestation of these psychophysiological changes over time and the temporal ordering of putative dynamic effects among symptoms are not known, yet crucial to provide mechanistic insights into PMDD and to enhance diagnosis precision and guide treatment individualization. The present study, thus, sought to profile premenstrual symptomatology by assessing its day-to-day intra-individual symptom-specific temporal pathways and dynamics among patients prospectively diagnosed with PMDD, in comparison with controls without significant premenstrual symptoms.

**Fig. 1:**
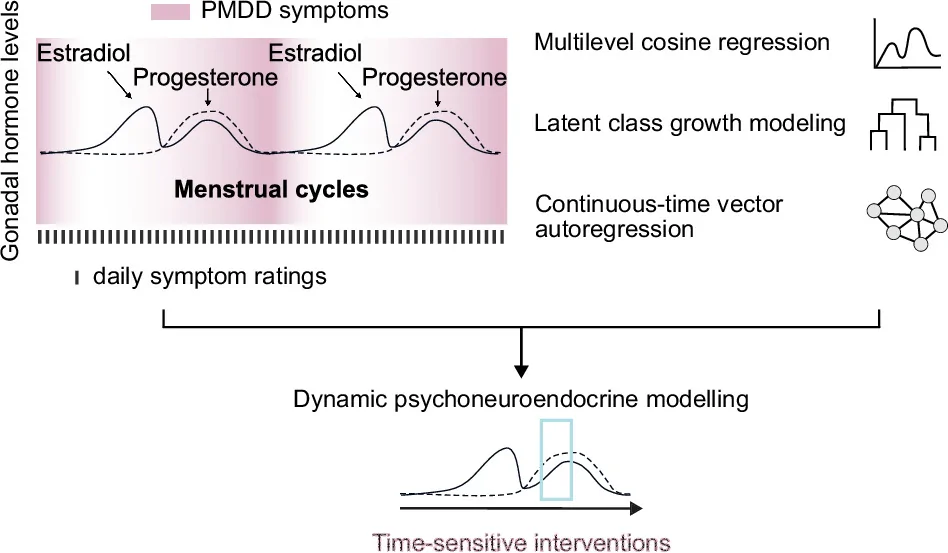
Temporal and dynamic systems modeling of PMDD symptoms. Daily symptom ratings across menstrual cycles in patients with PMDD and controls were leveraged using multilevel cosine regression, latent class growth modeling, and continuous-time vector autoregression. Bridging psychoneuroendocrinology with computational psychiatry contributes to advancing mechanistic understanding of psychiatric symptom variability and to informing precision time-sensitive approaches to people who menstruates’ mental health care. The present findings point at PMDD as a temporally heterogeneous syndrome with trajectories and dynamic interactions that unfold differently across symptoms and individuals.

Three questions were addressed in the present study: (i) How do specific symptoms of PMDD vary across the menstrual cycle in patients with PMDD compared to controls, and how much variation exists within and across patients with PMDD?; (ii) Do distinct temporal patterns of PMDD symptoms exist and how do these temporal patterns relate to each other?; and (iii) How is the level of severity of one symptom associated with the rate of change of another symptom (or itself) and how does the level of severity of one symptom predict the severity of another symptom (or itself) at a later specific point in time (Fig. 1). The findings point to within-person temporal patterns of PMDD symptom profiles, symptom interdependencies, and directional influences, thus contributing to a deeper understanding of PMDD’s underlying mechanisms and supporting the development of more refined differential diagnoses and personalized treatments.

## Results

Participants´ characteristics are presented in Supplementary Table 1. Patients reported an average age of PMDD onset of 24 years (SD 6.2), and the majority reported to have tried pharmacological or psychological treatment for their disorder in the past. The total DRSP score in the mid-follicular (day +7 to +11) and premenstrual phase (day −5 to −1) for patients with PMDD was 29 (SD 8.6) and 69 (SD 19), respectively. Corresponding total DRSP scores for controls were 26 (SD 6.2) in the mid-follicular phase and 28 (SD 6.7) in the perimenstrual phase (Supplementary Table 2).

### Premenstrual symptom variability across the menstrual cycle

**Fig. 2:**
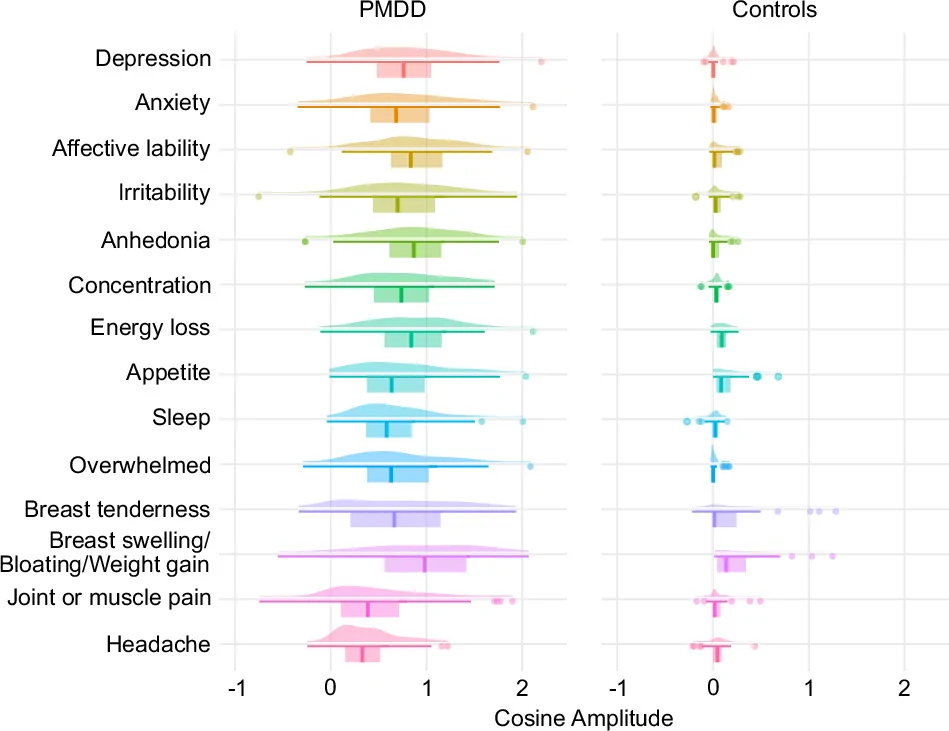
Variability and severity of premenstrual symptoms across the menstrual cycle in patients with PMDD and controls. Visualized are the estimated values of cosine amplitudes (*x*-axis) for patients with PMDD (*n* = 90) and controls (*n* = 44) yielded from the final multilevel cosine regression models for each individual symptom (*y*-axis). The cosine amplitudes correspond both to the timing and severity of symptoms across the menstrual cycle. Timing is indicated by the sign of the value, where positive values indicate a premenstrual increase of symptoms, negative values correspond to a mid-cycle increase of symptoms, and values of zero indicate no variation in symptomatology across the menstrual cycle. Severity is indicated by the magnitude of the cosine amplitude value, where larger/more negative values correspond to a higher severity of symptoms premenstrually/mid-cycle, respectively. Each box represents 50% of the data, with the median line in bold. The whiskers extend to the minimum and maximum of the data, except for those with outliers, which are represented by the dots. The violin plot illustrates the density distribution of the cosine amplitudes (i.e., a flatter curve indicates that the values are more spread along the values on the *x*-axis). The cosine regression models that the cosine amplitudes stem from are estimated using a proportional time variable that is scaled differently in the follicular and luteal phases depending on each individual’s length of each phase when setting ovulation to day −15.

A majority of the patients with PMDD (*n* = 90) showed a premenstrual increase in symptoms and thus a W-shaped curve across two menstrual cycles, as indicated by the pattern of positive cosine regression coefficient values (Fig. 2). A great range of values was observed among patients with PMDD, while controls (*n* = 44) demonstrated cosine regression coefficients near zero, with little variability.

Significant positive correlations were found across all symptoms in patients with PMDD (*r*(88) = 0.37–0.90; all *p* < 0.001; Supplementary Table 3), with the strongest correlations observed among the core affective symptoms, especially between depression and anxiety and between depression and affective lability (Supplementary Fig. 1). Only a few significant symptom correlations were noted in controls (*r*(42) = 0.37–0.88; *p* < 0.001 to *p* = 0.049; Supplementary Table 3), with no clear pattern regarding symptom type (Supplementary Fig. 1).

**Fig. 3:**
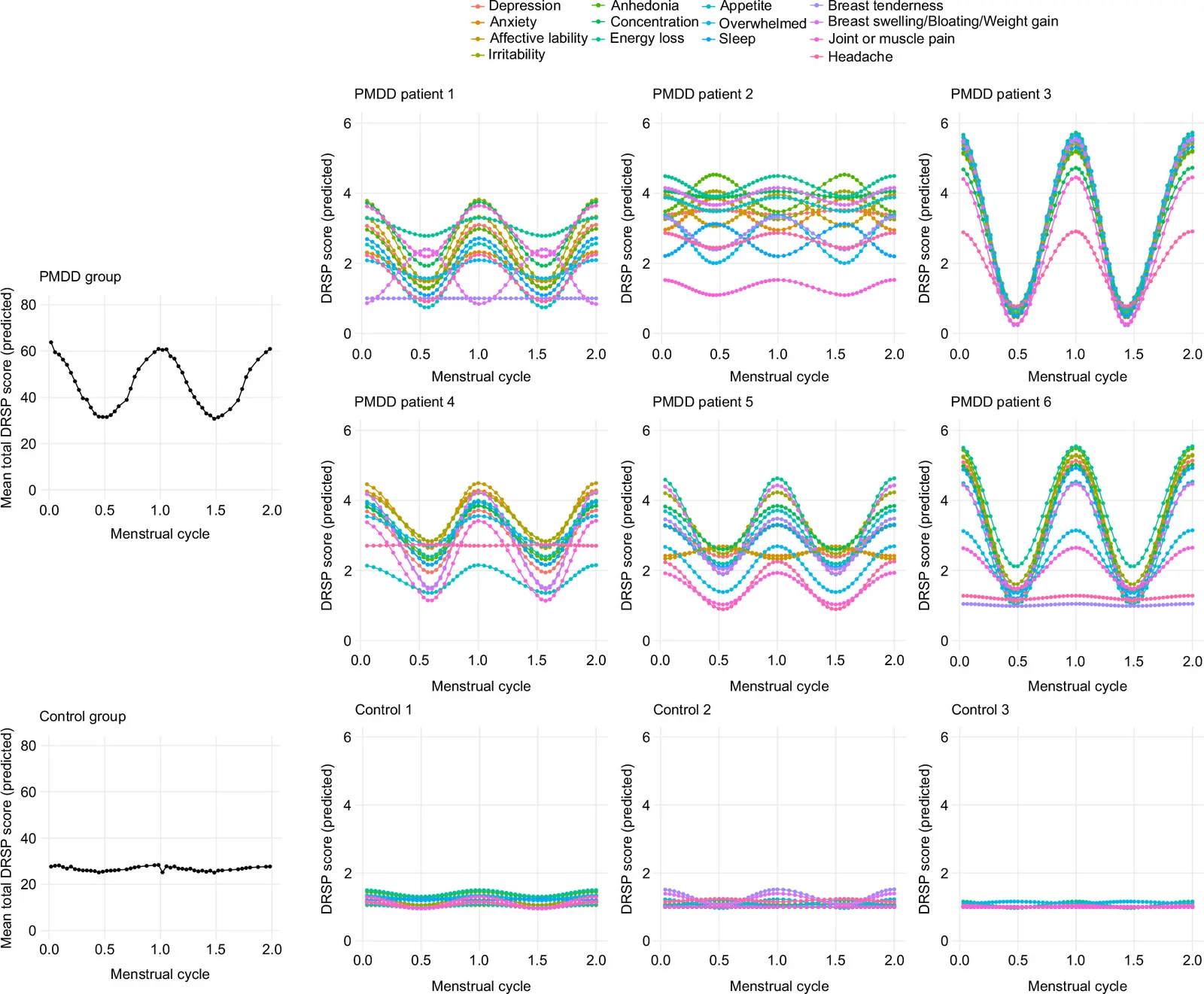
Examples of heterogeneity in variation in symptom severity over two menstrual cycles. The two graphs in the left panel show the average cosine regression curve of the total DRSP score for all patients with PMDD and controls. In the right panel, there are six examples of cosine regression curves of patients with PMDD over two menstrual cycles for each PMDD symptom, and three examples of controls, based on individual cosine regression models. The cosine regression curves represent how the symptoms vary over the two menstrual cycles, with steeper curves representing a more marked increase/decrease between the symptomatic and the asymptomatic phase. A W-shaped curve indicates a premenstrual increase of symptom, while an M-shaped curve indicates a midcycle increase of symptom. The timescale is proportional and scaled differently in the follicular and luteal phases based on each individual’s length of the two phases when setting ovulation to day −15, with 0 representing the first day of the first cycle, 1 representing the last day of the first cycle, and 2 representing the last day of the second cycle. The scores are predicted from the cosine regression models and do therefore not directly reflect the raw DRSP scores.

Despite the strong evident premenstrual increase in symptoms among patients with PMDD, variability among and within patients with PMDD was observed (Fig. 3). Some individuals showed consistent strong premenstrual increases across all symptoms, while others had more variable symptom profiles with different magnitudes of symptom change or less consistency in the cyclical timing across symptoms (e.g., with some symptoms showing mid-cycle increases).

Individuals differed from one another in their average symptom severity across time. For all symptoms, this between-person variability accounted for a larger proportion of the total variance among controls than among patients with PMDD. Moreover, and as expected, the menstrual cycle explained much more of the variance in symptoms among patients with PMDD than in the control group, and this was true for both the fixed effects (averaged across participants) and random effects (individual differences) of the menstrual cycle (Supplementary Tables 4–5).

In addition, differences across symptoms within the PMDD group were also observed (Supplementary Table 4). For example, variance explained by the fixed effects of the menstrual cycle, which was significant for all symptoms (*p* < 0.001), ranged from 6.3% for headaches to 17% for affective lability, 18% for depression, and 23% for breast swelling/bloating/weight gain. Similarly, variance explained by the random effects of the menstrual cycle (individual differences in menstrual cycle effects) was also significant for all symptoms (*p* < 0.001), ranging from 12% for headaches to 24% for affective lability, 25% for depression, and 33% for breast swelling/bloating/weight gain. Notably, the random effects of the menstrual cycle were larger than the fixed effects, implying that a larger proportion of the variance is explained by between-person differences than by a common tendency. Cosine regression models estimated with a uniformly scaled proportional time variable yielded virtually identical results (Supplementary Note 1; Supplementary Fig. 2; and Supplementary Tables 6–7), likewise age-adjusted analyses (Supplementary Note 1 and Supplementary Fig. 3).

Between-group differences in cyclical patterns were confirmed for all symptoms by a significant interaction between time and group (PMDD vs. control) (p < 0.001 across symptoms; Supplementary Table 8). Complementary Generalized Additive Mixed Models (GAMM), using both PACTS-scaled and uniformly scaled proportional time variables, captured the average observed pattern of symptom change across the menstrual cycle and further supported the robustness of the cosine regression findings (Supplementary Note 1; Supplementary Figs. 4–6; and Supplementary Tables 9–10).

### Temporal patterns and clustering of PMDD symptoms

**Fig. 4:**
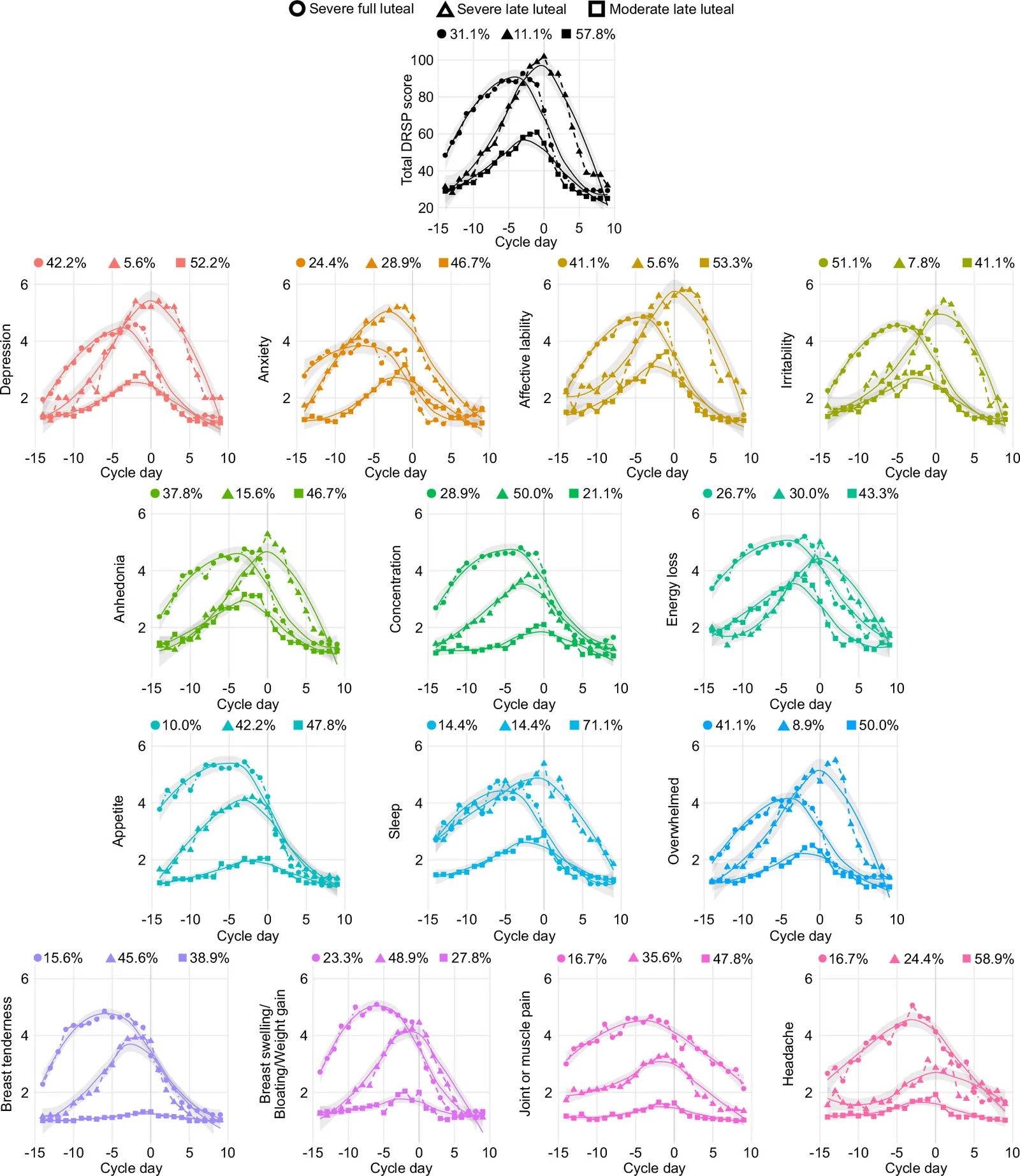
Temporal patterns of premenstrual symptoms. The top graph shows the temporal patterns obtained from the Latent Class Growth Analysis model based on total DRSP scores, while the colored ones show the temporal patterns for each PMDD symptom. The different patterns of each model do not necessarily include the same participants. All models are based on *n* = 90 patients with PMDD. Lines with circles/triangles/squares show the average score for menstrual cycle day −14 to +9 of participants included in each pattern, while solid lines show a smoothed curve with a shaded 95% confidence interval. Generally, the severe full luteal pattern displays an early luteal increase in severe symptoms persisting throughout the luteal phase, with an early offset after menses. The severe late luteal pattern generally has a mid/late luteal onset of severe symptoms and a late offset after menses onset for most symptoms, however with an earlier offset for some symptoms. Lastly, the moderate late luteal pattern generally displays a late luteal increase of moderate symptoms and an offset shortly after menses onset, however, this pattern was nearly asymptomatic for a few symptoms.

Having established substantial individual heterogeneity in symptom cyclicity, we next examined whether recurring temporal symptom profiles could be identified across patients and symptoms. Based on temporal variations in the total DRSP score and separate PMDD symptoms, patients (*n* = 90) were assigned to three general temporal patterns: severe full luteal, severe late luteal, and moderate late luteal (Fig. 4). Classification accuracy was indicated by the average posterior probabilities of group membership ranging between 0.87 and 0.99, and entropy ranging between 0.82 and 0.99 (Supplementary Table 11), which indicates good classification. Across the majority of symptoms, the severe full luteal pattern was characterized by an increase in severe PMDD symptoms in the early luteal phase persisting throughout the luteal phase, with an offset shortly after menses onset. The severe late luteal pattern was characterized by an increase in severe PMDD symptoms in the mid- or late luteal phase and later offset of symptoms for most symptoms (some symptoms demonstrated an earlier offset). Finally, the moderate late luteal pattern was characterized by a moderate increase of PMDD symptoms in the late luteal phase, with an offset of symptoms shortly after the start of menses. The moderate late luteal pattern was associated with very low severity scores for some symptoms (Fig. 4). Age-adjusted analyses yielded similar results (Supplementary Note 1; Supplementary Fig. 7; and Supplementary Table 12).

Qualitative and temporal differences were observed across the three temporal patterns in individual symptoms (Fig. 4). The core symptoms, along with anhedonia and feeling overwhelmed, had severe full luteal and severe late luteal patterns with clear temporal differences in the symptom peaks and resolutions: early onset and early offset vs. later onset and late offset around 4-5 days after the start of menstruation, respectively. In contrast, concentration difficulty, appetite disturbance, breast tenderness, and breast swelling/bloating/weight gain were characterized by a temporal alignment in the peak and resolution of these two patterns. Thus, for these later symptoms, these two temporal patterns were only differentiated by the onset of symptoms, not by the timing of the peak or by the resolution. Additionally, minimal premenstrual change and nearly asymptomatic levels characterized the moderate late luteal pattern for physical symptoms.

Temporal pattern proportions also varied across symptoms (Table 1). Among the core PMDD symptoms, the severe full luteal pattern was the most common pattern for irritability (51%), while the moderate late luteal pattern was the most common for the other core symptoms (depression (52%), anxiety (47%), and affective lability (53%)). Notably, the severe late luteal pattern was more commonly displayed for anxiety (29%) than for the other core symptoms, though with an earlier onset. Secondary symptoms displayed large variation in proportions across temporal patterns. Muscle pain and headache were most frequently assigned to the moderate late luteal pattern (48% - 59%), and sleep disturbances confined to this pattern in more than 71% of patients.

**Table 1:** Proportions of temporal patterns across symptoms

Symptom	Proportion of temporal pattern (%)		
	Severe full luteal	Severe late luteal	Moderate late luteal
Total DRSP score	31.1 %	11.1 %	57.8 %
Depression	42.2 %	5.6 %	52.2 %
Anxiety	24.4 %	28.9 %	46.7 %
Affective lability	41.1 %	5.6 %	53.3 %
Irritability	51.1 %	7.8 %	41.1 %
Anhedonia	37.8 %	15.6 %	46.7 %
Concentration	28.9 %	50.0 %	21.1 %
Energy loss	26.7 %	30.0 %	43.3 %
Appetite	10.0 %	42.2 %	47.8 %
Sleep	14.4 %	14.4 %	71.1 %
Overwhelmed	41.1 %	8.9 %	50.0 %
Breast tenderness	15.6 %	45.6 %	38.9 %
Breast swelling/bloating/weight gain	23.3 %	48.9 %	27.8 %
Joint or muscle pain	16.7 %	35.6 %	47.8 %
Headache	16.7 %	24.4 %	58.9 %

Proportions of each of the three temporal patterns for *k* = 3 number of temporal patterns, which was the number evaluated as most optimal for the total DRSP score and all individual PMDD symptoms, presented for the total DRSP and each individual PMDD symptom.

**Fig. 5:**
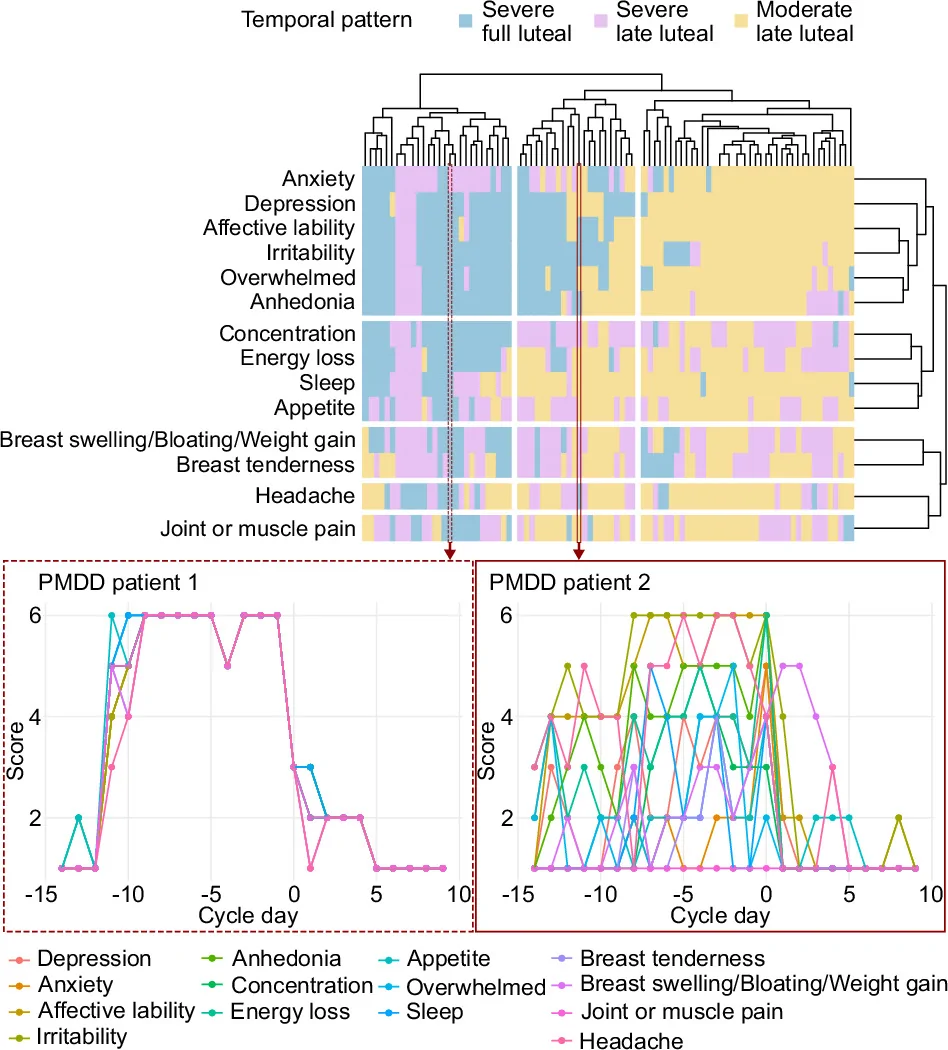
Clusters of symptom-specific temporal pattern memberships within and across patients. Building on the temporal patterns identified for each symptom using LCGA (Fig. 4), Fig. 5 illustrates how these patterns cluster across symptoms and across patients with PMDD, revealing the similarity and variability of temporal pattern membership across symptoms within and across individuals. In the upper panel, the heatmap and dendrograms show the result of the hierarchical clustering of the temporal pattern memberships across patients and symptoms. Blue represents the severe full luteal pattern (early onset and offset of severe symptoms); purple the severe late luteal pattern (mid/late onset of severe symptoms but with varying offset); and yellow the moderate late luteal pattern (late onset and early offset of moderate symptoms, but nearly asymptomatic for the physical symptoms). Columns of the heatmap represent individual patients, clustered into three groups based on similarities in their temporal pattern memberships across symptoms. The left vertical group shows predominantly the severe full luteal temporal pattern for psychological and vegetative symptoms, although with an exception of a few patients within the severe late luteal pattern for the same symptoms, but also for breast swelling/bloating/weight gain and breast tenderness. The middle vertical group shows a mix of all three temporal patterns across symptoms. The vertical group on the right shows predominantly the moderate late luteal pattern across symptoms, but with cases of the severe late luteal pattern mainly for vegetative and physical symptoms. The rows of the heatmap represent PMDD symptoms, clustered into five groups based on similarities in temporal pattern memberships across patients, separating psychological, vegetative, and physical symptoms, although with joint or muscle pain and headache emerging as separate clusters within the physical symptoms. Presented in the lower panel are examples of the raw DRSP scores from two patients with PMDD where patient 1 displays the same temporal pattern membership for all symptoms, and patient 2 displays varying temporal pattern memberships across symptoms (marked with dashed and solid lines in the heatmap, respectively). Lines of several symptoms may be overlapping, and therefore all symptoms might not be visible.

Given the symptom-specific variation in temporal patterns, we examined whether symptoms clustered according to shared temporal trajectories within individuals. Clusters based on participants’ patterns of temporal changes across symptoms and on individual symptoms’ patterns of change were identified through two-dimensional hierarchical cluster analysis (Fig. 5). Patients generally showed similar patterns of change across diverse symptoms within symptom groups. However, this similarity appeared to be strongest for the psychological and vegetative symptoms, where 39% and 23% followed the same temporal pattern, respectively. Regarding physical symptoms, the proportion following the same temporal pattern was 21%. However, headaches and joint or muscle pain emerged as separate clusters in the hierarchical clustering, and 68% followed the same temporal pattern for breast tenderness and breast swelling/bloating/weight gain. The majority of patients (98%) displayed a variable symptom profile, with different symptoms or symptom clusters following different temporal patterns; all symptoms followed the same temporal pattern in only 2% of patients.

### Temporal dynamics of core symptoms and predictive ability

Beyond identifying when symptoms emerge and resolve, it was assessed whether symptoms have dynamic temporal interdependencies over time among the patients with PMDD (*n* = 90). The temporal dynamics among the four core symptoms of PMDD were examined using Continuous-Time Vector Autoregressive (CT-VAR) modelling and interpreted as how the level of severity of one symptom is associated with the rate of change of another symptom (or itself), and how the level of severity of one symptom predicts the severity of another symptom (or itself) at a later specific point in time (lagged effect).

### Interpreting the daily dynamics using drift parameters

**Fig. 6:**
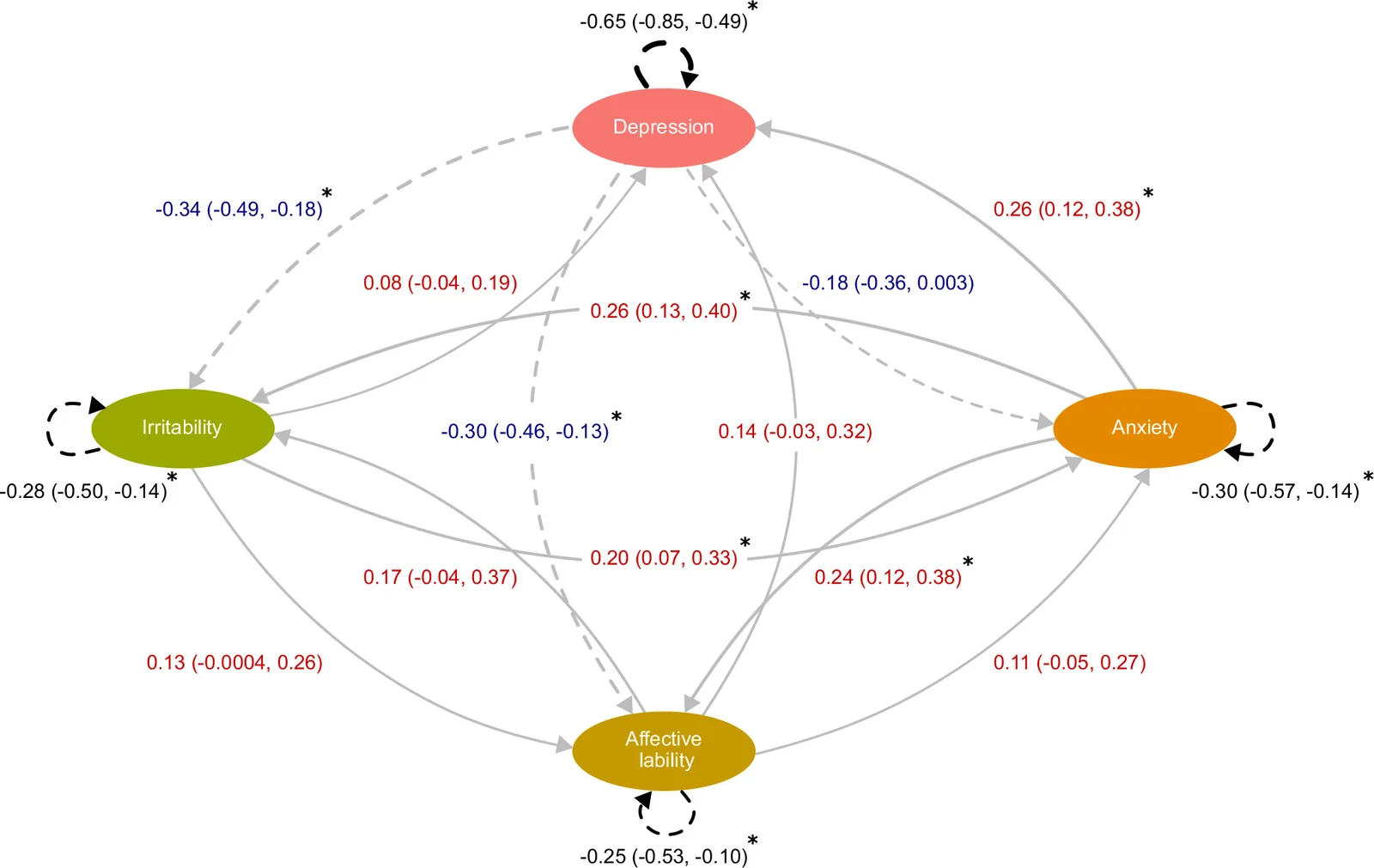
Dynamic network of the core symptoms in relation to each other, estimating how an increase in one symptom will affect another symptom in the next moment. Presented in the network are parameters representing estimates of daily dynamics, averaged across individuals, derived from the CT-VAR model of the four core symptoms of PMDD; depression, anxiety, affective lability, and irritability. These depict both the autoregressive effects, as well as how each core symptom is related to each other in a dynamic manner. The parameters between symptoms reflect the cross-symptom effect, which is the rate of change of how variation in one symptom affects variation in another from one moment to the other. The autoregressive effects reflect how each variable affects its own rate of change from one moment to the other. The grey lines display the effects between symptoms, and the black lines the autoregressive effects. Dashed lines with blue numbers are negative effects, and solid lines with red numbers are positive effects. Positive effects indicate that increase of the affecting symptom leads to a future increase of the connected symptom, as well as a decrease of the affecting symptom leads to a future decrease of the connected symptom; i.e., that changes in the connected symptom co-varies with the same changes as in the affecting symptom by being affected on its rate of change in the same direction. Negative effects however, indicate that an increase of the affecting symptom leads to a future decrease in the connected symptom, as well as a decrease of the affecting symptom leads to a future increase of the connected symptom, i.e., that changes in the connected symptom are inversely affected by the affecting symptom by being affected on its rate of change in the opposite direction. The temporal dynamic network is derived from one CT-VAR model including all four core symptoms of PMDD, estimated using Maximum A Posteriori optimization in ctsem with default regularizing priors, considering all days from the most severe cycle for each patient with PMDD (*n* = 90). The regular time variable in days was used, and a low/no symptom state was used as equilibrium. * Indicate effects that were significant after correction for multiple testing using the Benjamini-Hochberg correction.

We found significant autoregressive and cross-symptom associations with the rate of change in symptom state for the four core symptoms of PMDD (Fig. 6), based on the most symptomatic cycle, indicating daily dynamics. The autoregressive effects were negative for all symptoms (−0.65 to −0.30; *p* < 0.001 to *p* = 0.036; Supplementary Table 13), indicating that these symptoms tend to go back towards equilibrium (as is the case after onset of menses). Depression had the strongest negative autoregressive effect, indicating a faster rate of return towards equilibrium than other core PMDD symptoms.

Most significant cross-symptom effects were positive, with anxiety showing significant positive effects on all the other three core symptoms, with effects ranging from 0.24 to 0.26 (*p* < 0.001; Supplementary Table 13). This suggests that anxiety is a potential dynamic driver of the rate of change of the other core symptoms. Complementary analyses with anxiety and secondary psychological symptoms (anhedonia, concentration difficulties, and feeling overwhelmed) and anxiety with vegetative symptoms (energy loss, appetite, and sleep) showed that anxiety also was associated with the rate of change of anhedonia, concentration difficulties, and all vegetative symptoms with the same direction and similar effect sizes (Supplementary Fig. 8). Irritability also had a significant positive cross-symptom effect but only on the rate of change of anxiety, with a slightly lower effect of 0.20. Thus, the co-occurrence of irritability and anxiety was associated with increases in the rate of change of the other symptom. On the contrary, depression showed significant negative effects on affective lability and irritability, with effects of −0.30 and −0.34, respectively, suggesting a dampening effect of depression on the other two symptoms’ rate of change. Affective lability did not have any significant association with the rate of change of the other core symptoms.

A sensitivity analysis using the less symptomatic cycle yielded similar findings, suggesting robustness of the estimation of symptom dynamics across menstrual cycles (Supplementary Note 1 and Supplementary Fig. 9), likewise age-adjusted analyses (Supplementary Note 1; Supplementary Fig. 10; and Supplementary Table 14).

### Interpreting predictive abilities through lagged effects

**Fig. 7:**
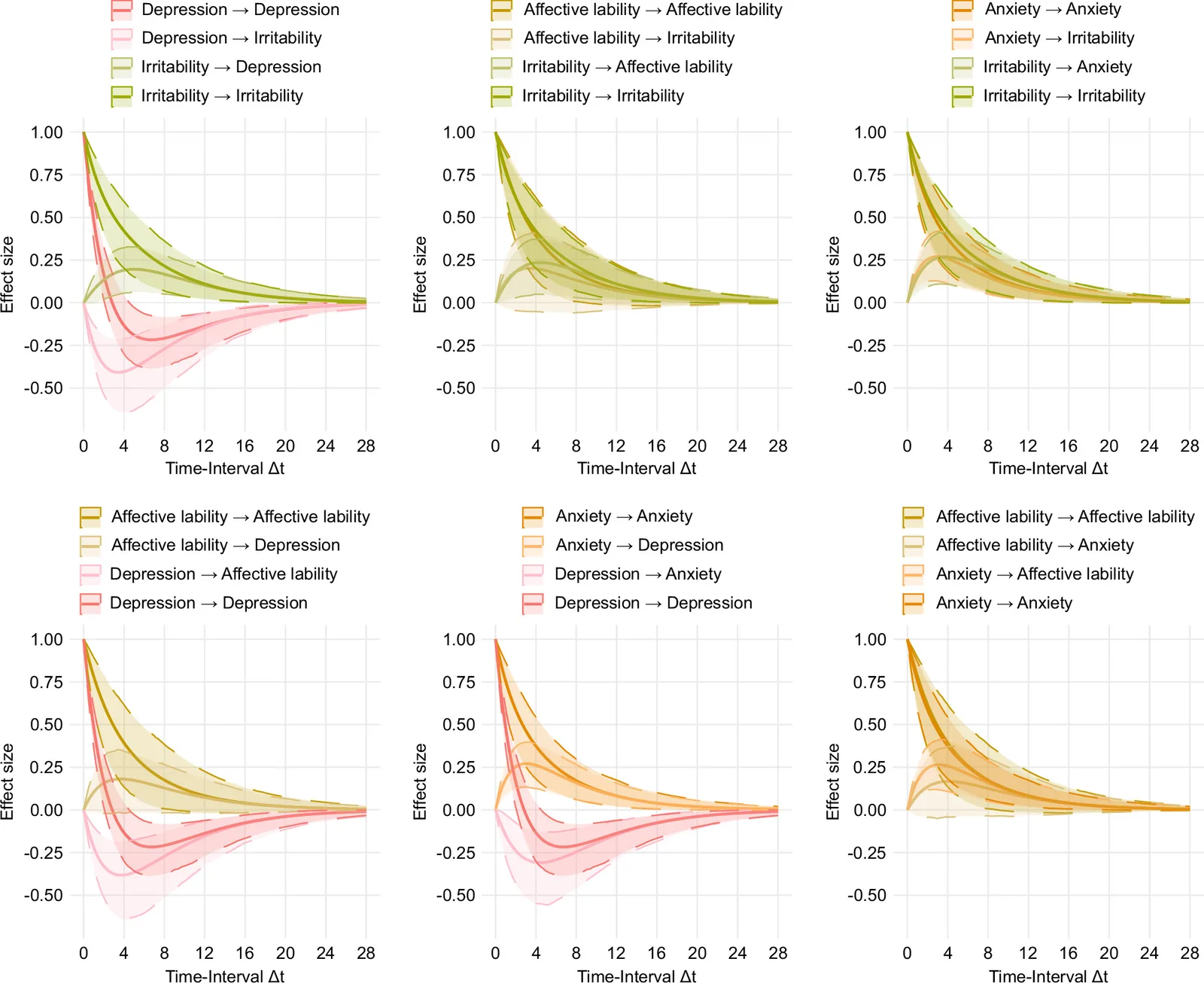
Lagged effects of the core symptoms of PMDD. The figures depict how each of the core symptoms of PMDD will predict the future values of itself and the other core symptoms depending on the time interval (days) between measurements across a range of time differences between 0 and 28 days. Shaded areas show the 95% confidence intervals. The graphs are derived from one CT-VAR model including all four symptoms, estimated using Maximum A Posteriori optimization in ctsem with default regularizing priors, considering all days in the most symptomatic cycle for each patient with PMDD (*n* = 90). A regular time variable in days was used in the model, and the low/no symptom state was used as equilibrium.

Lagged autoregressive (self-predictive) and cross-symptom (predictive) effects estimated at various time intervals (∆t) across an underlying continuous and dynamic process indicated how the different core symptoms of PMDD were associated with each other at different time lags, reflecting predictive ability among individual symptoms (Fig. 7). Signs of effect sizes stem from the drift matrix parameters, and their absolute values represent the magnitude of the lagged association from one symptom to another (or itself).

The lagged autoregressive effect for depression had the steepest decline among the four core symptoms, indicating that depression, compared to anxiety, affective lability, and irritability, changes more rapidly and can predict its own future values at shorter ∆t’s. Anxiety, affective lability, and irritability all showed self-predictive effects that decreased over a range of ∆t of 0–10 days, suggesting more stable tendencies across the order of days.

The lagged cross-symptom effects revealed significant predictive effects between all four core symptoms, except from affective lability to the other core symptoms. Across significant cross-symptom results, the peak effect sizes were strongest within ∆t of 3–5 days (Supplementary Table 15). The predictive ability of depression on the other symptoms had the highest maximum absolute effect sizes for all lagged cross-symptom effects, ranging between 0.31 and 0.41, with a peak at 3.4–4.4 days. Irritability predicting depression had the longest ∆t of 5 days, although with an effect size of 0.20.

## Discussion

These findings on temporal dynamics of psychiatric symptoms provide a nuanced perspective of the pathophysiology and symptom dynamics of PMDD in four key areas: the psychoneuroendocrine structure of symptom expression, PMDD definition, evidence for dissociable neurological vs physiological underpinnings, and understanding of the temporal ordering of putative dynamic effects among symptoms.

First, cyclical symptom changes across menstrual cycles, captured using multilevel cosine regression analyses, underscore the importance of symptom and temporal specificity—demonstrated here for prospectively, stringently defined patients with PMDD (Figs. 2 and 3). By capturing the premenstrual peaks and postmenstrual resolution, daily-level modelling confirmed W-shaped cyclical fluctuations in patients with PMDD, with pronounced interindividual heterogeneity in the severity and presence of specific symptoms (12–33% in the range of variance across symptoms) (Supplementary Table 4). This has to date only been explored in population-based samples of people who menstruate (without considering PMDD)^20–22^. In addition to the W-shaped pattern, the cosine approach also allowed detection of mid-cycle M-shaped symptom increases, which reflect periovulatory symptom changes. Because ovulation was not biologically confirmed in the present study, cosine regression models were estimated using a phase-aligned cycle time scaled (PACTS) variable assuming ovulation at day -15 across individuals^26–28^. As the vast majority of cosine amplitudes were positive for all symptoms in patients with PMDD (94–100%; Fig. 2), reflecting predominant W-shaped patterns, potential misalignment around the periovulatory phase likely had a minor, non-systematic impact on the results. The strongest cyclical effects were noted for affective and physical symptoms, especially depression, breast tenderness, and breast swelling/bloating/weight gain (Supplementary Table 4), underscoring hormonally modulated neural systems and physical changes. These findings therefore support the need for an integrated model of the psycho-somato-neuroendocrine structure of PMDD symptom expression^9,20,24^. In comparison, controls showed mild premenstrual increases of appetite, breast tenderness and breast swelling/bloating/weight gain, for which the discrepancies in variance explained by the menstrual cycle between patients with PMDD and controls were less prominent than for other symptoms such as sleep and energy loss (Supplementary Tables 4–5). Mild mid-cycle symptom increases were more common for sleep, joint or muscle pain, and headache in controls (Fig. 2).

The second key contribution of this study is the refinement of the definition of PMDD by identifying symptom-specific temporal patterns and evidence of cross-symptom connections within the symptomatic time frame (day −14 to +9, centered around menstruation onset). Three general temporal patterns for the total DRSP score emerged: severe full luteal, severe late luteal, and moderate late luteal (Fig. 4); they differed in severity, timing, and persistence. As the focus of this analysis was on premenstrual dynamics and the luteal phase is assumed to be relatively stable^27,28^, the results are likely less sensitive to ovulation timing. Furthermore, these results were consistent with those found in an independent sample for total DRSP scores^24^. Interestingly, temporal patterns varied not only across individuals, but also across symptoms within individuals, underscoring the multi-dimensional heterogeneity of the disorder (Fig. 5). For example, a patient may exhibit the severe full luteal pattern for irritability while displaying a severe late luteal pattern for depression, and a nearly asymptomatic moderate late luteal pattern for physical symptoms (Fig. 5). This diverse temporal unfolding of symptoms suggests that PMDD should be conceptualized as a spectrum of temporally patterned symptom profiles, rather than a single homogeneous condition. This variability, especially when considering core mental vs. physical symptoms, highlights the need to study multiple physiological pathways that may or may not converge, creating patterns of change across symptoms that may be very focal and synchronized, or generalized and affecting multiple systems and tissues each with a different temporal course. Acknowledging this extensive heterogeneity, not only between but also within individuals across symptom domains^21^, is essential for developing more accurate diagnostic assessments and tailoring treatment strategies to the timing and trajectory of each symptom.

Another interesting result of the present study is the varying proportions among the temporal patterns across symptoms (Fig. 4 and Table 1). Regarding core symptoms, the most common temporal pattern was the moderate late luteal pattern for depression, anxiety, and affective lability, while symptoms of irritability more prevalently showed the severe full luteal pattern. A high number of patients for anxiety (29%), and some secondary symptoms, were included in the severe late luteal pattern, although for anxiety the onset of symptoms was earlier compared to the other core symptoms. This is in line with the assumption that increased anxiety in the mid/late luteal phase could be due to differential sensitivity of the GABA_A_ receptor to the fluctuations of allopregnanolone levels and supports the trigger role of anxiety, as also the severe full luteal pattern shows an earlier onset compared with the other core symptoms^24^. Proportions of secondary symptoms varied. Interestingly, the majority of patients with PMDD displayed a moderate late luteal temporal pattern characterized by very low symptom severity for joint or muscle pain and headache (48% and 59%, respectively). These findings suggest that joint or muscle pain may be less central to PMDD than currently assumed. This may also warrant reconsideration of how physical symptoms are weighted within PMDD diagnostic frameworks and commonly used symptom rating tools such as the DRSP^29^, which includes the additional symptom of headache.

The current findings can be interpreted in light of aspects of the Dimensional Affective Sensitivity to Hormones across the Menstrual Cycle (DASH-MC) framework^25^, supporting heterogeneity in temporal symptom patterns and hormonal sensitivity, consistent with prior work in non-clinical samples^21^. Irritability showed a predominant severe full luteal pattern, which aligns with the luteal-onset sensitivity dimension and previous findings^24^. Notably, anxiety emerged as a central symptom in the CT-VAR analyses despite not being central in DASH-MC, suggesting that existing dimensional models may not fully capture dynamic symptom interactions in PMDD. Depression showed partial correspondence with prior hypotheses, here predominantly luteal rather than perimenstrual timing^24,25^. Furthermore, unlike^24^, the absence of asymptomatic patterns for anxiety and irritability may reflect the more stringent symptom threshold used here^30^. The present findings apply to a prospectively confirmed PMDD sample, using a threshold of >50% symptom increase from the follicular to the luteal phase, which may limit generalizability to less severe PMDD presentations but enhances diagnostic specificity and reduces false positives^30^.

The third key area of contribution concerns the correlations between cyclical changes across diverse symptoms and clusters of temporal patterns across symptoms^14,31^. Psychological and vegetative symptoms clustered separately from all four physical symptoms (Fig. 5), suggesting multiple, partially independent, neurobiological pathways mediating affective vs. somatic sensitivity to hormonal changes. Among the physical symptoms, temporal patterns of joint or muscle pain and headache did not cluster together with the other physical symptoms (Fig. 5), in line with lower covariation of cyclical changes with the other physical symptoms (Supplementary Fig. 1). This aligns with the idea that PMDD may arise from differential sensitivity of distinct brain systems^19–22,32^—such as limbic circuits for emotion regulation but somatosensory networks for physical discomfort^31^—to hormonal fluctuations. The menstrual cycle’s clear impact on the core affective symptoms in patients with PMDD (Fig. 2) is consistent with conceptualizing PMDD as an affective disorder. Among the physical symptoms, breast tenderness and breast swelling/bloating/weight gain were to a greater extent influenced by the menstrual cycle than headaches and pain (Supplementary Table 4). This is hardly surprising, as the underlying bodily functions (e.g., mammary glands) are directly influenced by female sex hormones and have a clear role in reproduction^33^. As premenstrual increases of these domains were also observed in healthy controls, these might be interpreted as female-, rather than PMDD-specific. The effect size, however, was much stronger for the patients with PMDD than the controls, providing strong support for the idea that PMDD involves a general hormonal sensitivity affecting multiple systems.

The fourth contribution regards the insights this study provides into a temporal ordering of symptom change in PMDD. CT-VAR modelling, capturing deviations during the premenstrual phase from each individual’s low/no-symptom state in the follicular phase, revealed temporally ordered associations among the core PMDD symptoms across adjacent observations, whereby the worsening of one symptom was associated with subsequent worsening of other symptoms. As CT-VAR models within-person symptom dynamics rather than precise cycle transitions, these findings are likely less sensitive to ovulation misalignments (Fig. 6). Results based on the cross-symptom effects identified anxiety as a potential dynamic driver of the other symptoms’ rate of change, suggesting that increases in anxiety were associated with accelerated worsening of the other three core symptoms, as well as five of the secondary psychological symptoms. This is consistent with the LCGA findings, where the severe full luteal and severe late luteal patterns together accounted for the majority of participants, both showing an earlier luteal onset compared to the other symptoms, suggesting that anxiety may emerge earlier in the luteal phase. This is further supported by findings pointing at the corticolimbic circuit as being anatomically shaped and functionally modulated by gonadal hormones and showing alterations in PMDD that partly overlap with neuroimaging findings on anxiety disorders^14^. Additionally, irritability was associated with increases in the rate of change of anxiety, suggesting a potential amplifying escalation loop between irritability and anxiety, though anxiety had a larger effect.

Depression occupied a distinct temporal position relative to the other core PMDD symptoms, appearing as facilitator of resolution of affective lability and irritability and to be more self-correcting than the other three core PMDD symptoms, as it displayed the fastest rate towards the low/no symptom state in the estimated autoregressive effects of the CT-VAR model (Fig. 6). However, as anxiety was associated with increases in the rate of change of depression, symptoms of anxiety experienced at the same time as depression could delay the resolution of depression. While depression appeared more self-correcting, anxiety, affective lability, and irritability could be interpreted as becoming entrenched in more stable states that are less dependent on immediate steroid fluctuations or daily environmental conditions, resulting in a delayed return towards the low/no symptom state.

While temporal associations in the CT-VAR models are described in terms of symptom-to-symptom influences for interpretive clarity, these findings cannot be interpreted as evidence of direct causal relationships between symptoms. An alternative explanation is that observed temporal dependencies reflect a shared underlying neuroendocrine process, such as cyclical fluctuations in ovarian hormones and neurosteroids, with different symptoms exhibiting distinct sensitivity profiles and temporal lags in response to these upstream biological changes. Under this interpretation, and consistent with the GABAergic sensitivity discussed above, anxiety may appear to precede other symptoms because affective and threat-related systems are closely linked to rapid modulation of GABAergic-mediated inhibitory tone by neurosteroids as allopregnanolone, which has been shown to influence both amygdala-based threat sensitivity and central stress–arousal regulation via effects on HPA-axis reactivity and neural excitability^34–37^. This has also been recently corroborated by PMDD having been associated with altered GABA_A_ receptor subunit plasticity (especially δ-subunit)^38^.

Additionally, the core mental symptoms, except affective lability, were predictive of each other’s future values, as a function of the time interval between observations (∆t) (Fig. 7). Cross-symptom predictive ability peaked at a range of ∆t of 3–5 days, though with moderate effect sizes, meaning that the predictive association between one symptom and another was strongest when observations were 3–5 days apart, depending on which symptom. Depression showed the largest effect sizes for all three cross-symptom predictive effects, while irritability showed the longest ∆t of 5 days between measurements for the prediction of depression. This suggests that depression was the best predictor of the subsequent severity of the other three core symptoms, but irritability could predict depression across the longest time interval between measurements. Such insights may help to forecast symptom escalation for patients with PMDD, by pointing to CT-VAR as a potential approach for identifying cross-symptom temporal relatioships and personalized interventions.

Treatment individualization can indeed be pursued through targeting those symptom(s) whose improvement could be expected to have the greatest impact on each individual’s overall symptomatology. Anxiety’s central role in the temporal dynamics of PMDD core symptoms, according to the present findings, positions it as a potential therapeutic target. Standard pharmacological treatment for PMDD is selective serotonin reuptake inhibitors^39,40^, and psychotherapeutic treatments evaluated for PMDD have adopted techniques, such as cognitive restructuring and behavioral activation^41,42^. While psychotherapy is an efficacious treatment for anxiety disorders^43^, anxiety-specific psychotherapeutic techniques have not yet been systematically evaluated for PMDD. However, the gold standard psychological treatment technique, i.e., exposure therapy, for anxiety disorders such as phobias or social anxiety disorder^44,45^, is unlikely to translate well into PMDD treatments, given that anxiety symptoms in PMDD are intermittent and progesterone-dependent, and not bound to a specific psychological stimulus. Empirical evaluation of the efficacy of emotion regulation and distress tolerance techniques, which can empower patients to observe, modulate, and withstand fluctuating emotions (depression, anxiety, irritability), is still missing for PMDD^46^. Similarly, anxiolytic therapy tailored for PMDD remains to be investigated^39^. Thus, according to the present findings, interventions combining SSRIs with psychotherapeutic strategies targeting anxiety (e.g., emotion regulation, distress tolerance) may disrupt the temporal interdependency between anxiety and irritability, and yield broader symptom relief, which should be further evaluated and confirmed within clinical trial settings.

In conclusion, by bridging psychoneuroendocrinology with computational psychiatry, the present findings advance our understanding of PMDD as a complex, cyclical disorder rooted in psychoneuroendocrine dynamics. These results suggest the presence of inter- and intra-individuality of PMDD symptoms, temporally patterned PMDD symptom profiles, symptom interdependencies and directional associations, including feedback loops, as well as predictive abilities between and within symptoms. They demonstrate how disaggregated daily symptom ratings can inform on symptom trajectories to neurophysiological systems sensitive to hormonal changes. Such knowledge provides insights into the sex-specific, hormone-sensitive, and individual mental health profiles, and the unfolding of heterogeneous symptoms over time and affective regulation, which we suggest will be central in understanding the idiosyncratic expression of PMDD.

Moving beyond static and homogeneous conceptualization of PMDD, these findings can inform future refinement of the PMDD diagnostic criteria and tools for daily symptom rating, and pave the way for temporally targeted, evidence-based treatments tailored to individual symptom profiles to prevent symptom escalation. By integrating dynamic symptom modelling with clinical practice, we could advance precision in the mental health care of people who menstruate, and reduce the burden of PMDD (Fig. 1). More broadly, these findings highlight PMDD as a promising model for studying hormone-sensitive psychiatric dynamics.

## Methods

### Participants

This observational, prospective study was performed as part of two double-blind randomized control trials, one on escitalopram or placebo, and one on selective progesterone receptor modulator (SPRM) or placebo, conducted at Uppsala University. All procedures were approved by the Regional Ethical Review Board in Uppsala (Dnr. 2016/184 and 2016/312, respectively) and the Swedish Medical Products Agency. The trials were registered as EDURA-CT 2016-001217-25 and EDURA-CT 2016-001719-19. All participants in both trials provided written informed consent. Data presented in this study is limited to the prospective daily rating phase of these studies. The present observational study was not separately preregistered.

Upon recruitment through advertisements in local newspapers, on university campuses, and social media, participants were assessed and enrolled. Inclusion criteria were menstruating individuals aged 18–46 with regular menstrual cycles, with a confirmed PMDD diagnosis or suspecting PMDD, no ongoing pregnancy, breastfeeding, or pregnancy planning, no ongoing or recent psychotropic or hormonal medication use (≥3-month washout), and no regular use of nicotine (e.g., smoking or using snus). Participants fulfilling these criteria were invited to a screening interview. Exclusion criteria encompassed ongoing DSM-5 disorders assessed via the Mini International Neuropsychiatric Interview (MINI)^47^, past psychiatric hospitalization or suicide attempts, and severe medical conditions. Past depressive or anxiety disorders were permitted. The MINI interview, as well as a Case Report Form (CRF) including questions on demographic variables reported in Supplementary Table 1, were completed during the screening.

Biological sex was operationalized through the study eligibility criteria, which restricted inclusion to participants with regular menstrual cycles, that is, with an ovulatory reproductive physiology. Gender was not assessed. Included individuals reported their symptoms rated on a 6-point Likert scale (1 = not at all, 6 = extreme), along with menstrual bleeding reports for two consecutive menstrual cycles, using the Daily Report Severity of Problems (DRSP^29^) via a web-based electronic patient-reported outcome system (Hytton Technologies AB, Sweden)^7^. The DRSP is based on the DSM-5 definition of the PMDD diagnosis, using the following four symptom domains: core affective symptoms (e.g., depressed mood, anxiety, affective lability, and irritability or anger); secondary psychological symptoms (e.g., anhedonia, concentration difficulty, and feeling overwhelmed); vegetative symptoms (e.g., appetite- and sleep disturbances and energy loss); and physical symptoms (e.g., breast tenderness, breast swelling/bloating/weight gain, joint or muscle pain, and headache) (Supplementary Table 16; for brevity the labels defined in this table are used throughout the text). Recruitment of controls was performed similarly, through the same community-based channels, with the same inclusion and exclusion criteria, screening procedure, and daily diary, except the presence of PMDD symptoms.

PMDD was stringently defined as a >50% increase in at least five symptoms from the follicular (days 6–12) to luteal phase (days −7 to −1), including at least one core affective symptom (depressed mood, anxiety, mood swings, irritability or anger). Using a >50% increase of symptoms ensured that all participants were above the disorder threshold^30^. Percent change was calculated as (mean luteal score − mean follicular score)/mean follicular score × 100. Using a >50% increase of symptoms ensured that all participants were above the disorder threshold^30^. Symptoms had to be at least mild (mean luteal score >3.0) and minimal/absent in the follicular phase (mean follicular score <2.0). Additionally, the experienced symptoms should have caused significant suffering or impaired daily functioning. Exclusion criteria for all participants were irregular menstrual cycles, pregnancy, breastfeeding, hormonal contraceptive or antidepressant use during the past three months, or an ongoing psychiatric disorder (besides PMDD).

Out of 125 potential patients with PMDD, 35 (28%) did not provide sufficient ratings during the two screening months and were thus excluded, leaving a final PMDD sample of 90 patients. As the data from the DRSP items were the main interest of analysis, a maximum of 20 missing days in total was considered acceptable (although only one included patient had 20 missing days), as long as the missing days were spread across the two menstrual cycles, that there were no gaps larger than 4 days missing in a row in the luteal/perimenstrual phase (days −14 to +2), and no gaps larger than 10 days missing in a row in one of the follicular phases (days +3 to +13), as long as the other follicular phase was more complete and confirmed the low/no symptom state (only one included patient had 10 missing days in a row in the follicular phase). The mean and standard deviation of total missing days across the two cycles among the 90 included patients with PMDD were 2.5 (4.3) days (range 0–20 days). The mean and standard deviation of gaps of missing days in a row in the luteal/perimenstrual phase were 0.67 (1.1). In the follicular phase, the mean and standard deviation of gaps of missing days in a row was 1.1 (2.1).

Similarly, out of 51 potential controls, 7 (14 %) were excluded due to insufficient ratings, leaving a final control sample of 44 individuals with a mean and standard deviation of missing days across the two cycles of 0.89 (1.7) days (range 0–7 days). In total, the present study included 134 individuals, who all met the eligibility criteria during the screening phases of the two parent trials; no separate sample size calculation was performed  for this observational study.

### Procedures

The DRSP^29^ was completed by participants through an online diary system. Total DRSP score for each day was computed as the sum of the 21 DRSP items, all of which were scored using the same response scale ranging from 1 (not at all) to 6 (extreme). DRSP scores for each DSM-5 domain of PMDD were computed by calculating the mean of the DRSP items belonging to each domain (Supplementary Table 16).

### Statistical analyses

All statistical analyses were performed in RStudio with R version 4.3.0^48^. Missing data for each DRSP question were imputed using spline interpolation, which is an imputation method suitable for regular time series data that has the ability to capture trends. Analyses for specific research questions are described in detail below.

#### Exploring symptom variability

Variability in menstrual cycle-related changes of PMDD symptoms was tested on a group-level across patients with PMDD (*n* = 90) and controls (*n* = 44), and between individuals within each group. Multilevel cosine regressions, applying the cosine function to the menstrual cycle and providing coefficients describing both cyclical pattern and symptom severity, were used to quantify cyclical variation of each PMDD symptom across two menstrual cycles in each group, and the individual differences in these changes. This approach was guided by prior empirical research in the general population^21^. A proportional time variable scaled differently in the follicular and the luteal phase based on the lengths of the two phases was used in the models. As biological confirmation of ovulation was not available, ovulation was set to day -15 across participants^26^. One unit was added to the second cycle, so the time variable ranged from 0 to 2, with 0 corresponding to the first day of the first cycle, 1 to the last day of the first cycle, and 2 to the last day of the second cycle. As a secondary analysis, cosine regression models were estimated using a proportional time variable which was uniformly scaled across the cycle (Supplementary Note 1).

Cosine regression models were implemented separately for each group using both the total DRSP score and individual symptom scores, and included random intercepts as well as the fixed and random effects of the cosine effect of time (effect of the menstrual cycle). Thus, these analyses capture the average change across the menstrual cycle in each group and the individual differences in these changes. The cosine regressions provide a single regression coefficient (representing the cosine amplitude) for each participant, describing the direction and severity of change across two menstrual cycles. Positive values represent a premenstrual increase of symptom, while negative values represent a mid-cycle increase, with larger absolute values corresponding to higher levels of severity.

To better understand the sources of symptom variability across and within individuals, two additional models were implemented: One only including random intercepts (model 1), and one including only the fixed effect of the cosine effect of time (model 2). These additional models were used to calculate estimates of variance explained by individual differences in average levels of symptoms (random intercepts, given by the intraclass correlation (ICC)), variance explained by the average effect of the menstrual cycle within each group (given by the average effect of cosine function of time), and variance explained by individual differences in menstrual cycle-related changes in symptoms (given by the random slopes of the cosine function of time). The effect sizes for random intercepts (model 1) were computed using intraclass correlation (ICC). By comparing the residual sum of squares (RSS) between model 1 and model 2, and between model 1 and the full model, effect sizes of the fixed and random effects of the cosine effect of time, respectively, were computed. Bonferroni correction was applied separately to the estimated *p*-values of the fixed and random effects of the cosine regression models to adjust for multiple comparisons across symptoms.

Two-sided Pearson correlations were tested between the cosine regression coefficients, separately for patients with PMDD and controls, to examine co-expression of cyclical symptom variation. Benjamini-Hochberg correction was applied to the correlation analyses to adjust for the number of pairwise correlations tested across symptoms (91 comparisons for 14 symptoms).

Finally, separate cosine regression models were estimated for each participant and symptom for graphic presentation of the cosine regression slopes. Additional cosine regression models including the interaction between time and group were estimated to statistically test whether cyclical symptom patterns differed between groups for each symptom. Lastly, as a complement to the cosine regression models, GAMMs were estimated to examine the explained variability and the shapes of the observed average patterns of change across symptoms (Supplementary Note 1).

#### Investigating temporal patterns and clusters of PMDD

Temporal patterns of PMDD symptoms were investigated based on symptom timing and severity, with additional analyses testing whether these temporal patterns cluster together across patients and across symptoms. First, Latent Class Growth Analysis (LCGA)^49^ was performed on the raw DRSP data of patients with PMDD (*n* = 90) using the FlexMix package in R^49^. Menstrual cycle days −14 to +9 were included in the models, where day 0 was the menstruation onset, to capture the symptomatic time-frame. Menstrual cycle days were computed using a combination of forward and backward counting, where day 0 was the first day of menses, as done in ref. ^24^. The number of temporal patterns was determined based on model fit criteria (the Bayesian Information Criterion (BIC) and the Akaike Information Criterion (AIC)) and temporal pattern percentages (>5%).

For these analyses, one model was estimated on the total DRSP score using a normal distribution, as well as a second set of models on each individual symptom of PMDD using Poisson distributions. Poisson distributions were used for the individual symptoms as these distributions are typically highly skewed in patients with PMDD. The analyses of temporal patterns were run in a stepwise manner, enabling the Expectation-Maximization (EM) algorithm to converge at the global maximum. The models included a mix of linear, quadratic, and cubic effects of time, and they were run for *k* = 1:4 number of temporal patterns. The BIC and AIC were used together with temporal pattern percentages (>5%) to evaluate models with different numbers of temporal patterns. Based on these criteria, *k* = 3 was chosen as the most appropriate number of temporal patterns for the total DRSP score and all individual symptoms. The average posterior probabilities, reflecting classification accuracy, of each temporal pattern in each model were controlled to be over the recommended threshold of 0.7^50^. Entropy was computed for each model as an additional measure of classification.

Secondly, the resulting temporal patterns for each PMDD symptom were clustered across PMDD symptoms and across patients with PMDD, using a two-dimensional hierarchical clustering. This was done to investigate how symptom-specific temporal patterns cluster together, and how patients cluster together based on their symptom-specific temporal patterns. LCGA analyses of the total DRSP score and the core PMDD symptoms were guided by prior empirical research^24^, whereas the hierarchical clustering of temporal pattern membership across symptoms was exploratory in nature.

#### Exploring PMDD symptom dynamics and predictive ability

Lastly, autoregressive (i.e., the association of each symptom with its own rate of change; e.g., depression → depression) and cross-symptom (i.e., each symptom’s association with the other symptom’s rate of change; e.g., depression → irritability) temporal dynamics were examined for the core PMDD symptoms (depression, anxiety, affective lability, and irritability). For this exploratory analysis, a multivariate CT-VAR model was implemented using the R package ctsem^51^, estimated with Maximum A Posteriori optimization and default regularizing priors. In CT-VAR, stochastic differential equations are used to estimate an underlying continuous and dynamic process that could provide meaningful interpretation at any moment of time in the timeframe^52^. The low/no-symptom state in the follicular phase for each individual was defined as the equilibrium. The model was limited to the core symptoms as these represent the primary defining features of PMDD, and including all symptoms would substantially increase the model complexity and number of parameters to be estimated, risking unreliable estimates given the sample size.

Raw DRSP scores for depression, anxiety, affective lability, and irritability for each day from the most symptomatic cycle (the cycle with the highest mean sum score of the four core symptoms over five consecutive days in the luteal phase), for each patient, were used in this model to estimate temporal dynamics. Each individual’s low/no-symptom state was defined as the equilibrium (defined as the days when each participant had the lowest scores), which the model strives towards after an increase of a symptom. The model provides a drift matrix that includes parameters describing the dynamic behavior between the variables included in the model. When interpreting the parameters, significant autoregressive and cross-symptom effects were considered simultaneously because of the multivariate nature of the model. These drift parameters were interpreted both directly and as lagged effects estimated from the drift matrix, both explained below. The analysis output indicates how the level of severity of one symptom is associated with the rate of change of another symptom (or itself) and how the level of severity of one symptom predicts the severity of another symptom (or itself) at a later specific point in time (lagged effect).

A sensitivity analysis using the less symptomatic cycle was performed to assess the robustness of the findings, as well as two extra CT-VAR models to assess the temporal relationship between anxiety and the secondary psychological symptoms and the vegetative symptoms of PMDD, respectively (Supplementary Note 1).

#### Interpreting drift parameters

Drift parameters were interpreted to examine daily dynamics in the system of the four core symptoms. The drift matrix includes both estimates of autoregressive effects (diagonal elements), which represent each symptom’s association with its own rate of change, and cross-symptom effects (off-diagonal elements), which represent each symptom’s association with the other symptom’s rate of change. Positive estimates indicate that increases of one symptom tend to co-occur with future increases in the connected symptom, while negative estimates indicate a co-occurrence of increases in one symptom with future decreases of the connected symptom. Significance of the drift parameters was determined from the 95% CIs. Benjamini-Hochberg correction was applied to the drift parameters to adjust for multiple comparisons across the autoregressive and cross-symptom effects.

#### Interpreting the lagged effects

Lagged effects at various time intervals between measurements (∆t) across the underlying continuous and dynamic process were calculated using the following equation: ${{\rm{Lagged\; effects}}}=\,{e}^{{{\bf{A}}}\Delta t}$. The lagged effects were calculated across a timeframe of ∆t = 0-28 days, discretized into steps of 0.1. Both lagged autoregressive and cross-symptom effects were estimated. Lagged autoregressive effects give an indication of ∆ts at which each symptom is predictive of itself. Lagged cross-symptom effects give an indication of the ∆ts at which each symptom is associated with changes in the other symptoms, and thus at which ∆ts each symptom is predictive of the other symptoms. The peaks in cross-symptom effect sizes over time indicate the ∆t where one symptom is most strongly associated with changes in the other symptom, and the predictive ability is thus highest at that ∆t. The decline in autoregressive effects indicate how fast the self-predictive effect of each symptom levels off.

### Reporting summary

Further information on research design is available in the Nature Portfolio Reporting Summary linked to this article.

## Supplementary information

Supplement

Reporting Summary

Transparent Peer Review file

## Source data

Source Data

## Data Availability

All data used to reach the conclusions presented in this study are presented in the main text or the supplement. The raw data collected and analyzed during the current study are only available under restricted access due to participant privacy and ethical restrictions, access can be obtained for research purposes by contacting the corresponding author, with expected timeframe for response to access requests of ninety days, subject to appropriate ethical approval and data-sharing agreement that will specify for how long the data will be available once access has been granted. The generated data underlying the figures in this study are provided in the Source Data file. Source data are provided with this paper.

## Code availability

Custom code is publicly available at https://doi.org/10.5281/zenodo.22300503.
