# Supplementary material for: Temporal dynamics of premenstrual dysphoric disorder revealed through predictive symptom networks
Source: Nat Commun. 2026 Nov 10;17:11165. doi: 10.1038/s41467-026-78590-x (PMC13751610; doi:10.1038/s41467-026-78590-x)
Supplement: Supplementary file 1 [file 41467_2026_78590_MOESM1_ESM.pdf]

SUPPLEMENTARY MATERIAL

Temporal Dynamics of Premenstrual Dysphoric Disorder Revealed Through Predictive Symptom Networks

Ella Schleimann-Jensen, Inger Sundström-Poromaa, Johanna M. Hoppe, Sinha Engel, Jeff Kiesner, Erika Comasco

Supplementary Note 1 (Extra analyses): Page 2-7

Supplementary Tables: Page 8-34

Supplementary Figures: Page 35-43

SUPPLEMENTARY NOTE 1

Extra analyses

Cosine regression (proportional time variable scaled uniformly across the cycle)

Main cosine regression analyses were estimated using a proportional time variable that was scaled differently in the follicular and luteal phase depending on the lengths of the two phases, consistent with the approach of Phase-Aligned Cycle Time Scaling (PACTS) [1]. As biological confirmation of ovulation was not available, ovulation was set to day -15 across individuals. Moreover, as a secondary analysis, the cosine regression models were estimated using a proportional time variable computed by dividing cycle day by the full cycle length, representing the proportion of each cycle passed for each day of measurement [2]. As for the PACTS proportional time variable, one unit was added to the second cycle, so the time variable ranged from 0 to 2, with 0 corresponding to the first day of the first cycle, 1 to the last day of the first cycle, and 2 to the last day of the second cycle. This scaling approach yielded virtually identical results as when using a PACTS scaled time variable (Supplementary Figure 2, Supplementary Table 6-7), indicating that the results of cosine regressions were robust to the method of cycle scaling in this sample.

Cosine regression – Theoretical background

Cosine regression is a particular form of regression including a cosine effect of time in a generalized linear model (GLM). The cosine effect forces the data to follow a cosine curve which can be suitable when working with periodic data, like the menstrual cycle. A cosine function of the time T with period 2π, no shift, amplitude A, and intercept D, will look like $f(t) = A*\cos(2\pi*T) + D$. A linear model will estimate intercept and the slope (β) for each input, which means that in cosine regression β will equal the estimated value of the cosine amplitude. The cosine amplitude is the distance between the center of the cosine wave, to the top of the first peak/through, where a positive value of the cosine amplitude corresponds to a cosine wave with a through first, and a negative value corresponds to a cosine wave with a peak first. A premenstrual increase of symptom, will lead to a W shaped curve, i.e., a positive value of the cosine amplitude, which is the typical pattern for an individual with PMDD. A mid-cycle increase of symptom, would lead to an M shaped curve, i.e., a negative value of the cosine amplitude.

GAMM

Generalized Additive Mixed Models (GAMM) are flexible, data-driven regression models that can capture the average non-linear patterns of change across time using smooth functions, while accounting for the nested structure of the data. This analysis was performed as a complementary analysis to the cosine regressions, which forces the data into a sinusoidal shape, to capture the observed average pattern of change across individuals.

GAMM models were estimated using two cyclic proportional time variables consistent with the PACTS approach [1] scaled differently in the follicular and luteal phase based on the lengths of the two phases, ranging from -1 to 1, one centered around menstruation onset and one around ovulation (set to day -15 across individuals). Supplementary Figures 4 and 5 show the GAMM-estimated population average symptom trajectories across the standardized menstrual cycle centered around menstruation onset and ovulation, respectively, together with ±1SD of raw DRSP scores indicating variability around the average. Supplementary Table 9 and 10 include the total explained variances (R^2) for each symptom for patients with PMDD and controls, as well as the hierarchical partitioning of the proportions of fixed and random effects of the total explained variance, for the GAMM models centered around menstruation onset and ovulation, respectively. The total explained variances for the models estimated with time centered around menstruation onset was virtually identical to the total explained variances for the models estimated with time centered around ovulation across symptoms. That said, the proportions of fixed effects were consistently larger for the models centered around menstruation onset, meaning that the population average trajectory explains more variance when the cycle is centered around menses onset than around ovulation. On the other hand, the total explained variances were quite low across symptoms (range 0.15-0.32 for all models for patients with PMDD), indicating that there is substantial individual variability that the average symptom trajectories obtained with GAMM cannot explain. As for the cosine regressions, this confirmed that group-based modelling, such as performing LCGA analyses, is needed.

For controls, the GAMM models of the majority of symptoms showed ~0% fixed effects of the total explained variance, meaning that there was no consistent shared cyclical pattern across participants. This is expected as controls do not experience cyclical symptoms to the extent of

patients with PMDD. Only for appetite and the physical symptoms breast tenderness and breast swelling/bloating/weight gain, some of the variance could be explained by an average cyclical pattern, which is in agreement with the findings of the cosine regressions estimated with the PACTS scaled time variable (Supplementary Table 4-5).

Additionally, GAMM models were estimated using a proportional time variable scaled uniformly across each cycle, ranging from 0 to 2. The average pattern across the two cycles was similar to the average W-shape that was found with cosine regression estimated with the same time variable (Supplementary Figure 6), which suggests that cosine regression provides valid estimates of the shape of change across time, comparable to the ones of GAMM.

Additional CT-VAR analyses

The main CT-VAR model was estimated using the most symptomatic cycle for each individual. The rationale was to maximize signal-to-noise ratio and to ensure sufficient variability for dynamic modelling. However, as a sensitivity analysis, an additional CT-VAR model was estimated using the less symptomatic cycle for each individual. The less symptomatic cycle was the cycle with the lowest mean sum score of the four core symptoms over five consecutive days in the luteal phase. The key findings of the main CT-VAR analysis of the most symptomatic cycle were confirmed by the sensitivity analysis (Supplementary Figure 9): Depression showed a faster rate back to the low/no symptom state compared to the other symptoms (more negative drift parameter), and was associated with a dampening effect on the other core symptoms' rate of change. Anxiety was significantly associated with increases in the rate of change of the other three core symptoms, and together with irritability formed a feedback loop in which both symptoms were associated with increases in the other symptom's rate of change. This suggests that the key CT-VAR findings are robust across menstrual cycles.

To further examine whether the associations of anxiety extended beyond the core symptoms of PMDD, two complementary CT-VAR models were estimated. The first model included anxiety and the three secondary psychological symptoms included in the PMDD diagnosis (anhedonia, concentration difficulties, and feeling overwhelmed), and the second model included anxiety and the three vegetative symptoms included in the PMDD diagnosis (energy loss, appetite, and sleep).

Anxiety was significantly associated with increases in the rate of change of anhedonia, concentration difficulties, and all vegetative symptoms (Supplementary Figure 8), further supporting the central role of anxiety in the temporal dynamics of PMDD symptoms.

Sensitivity analyses adjusting for age

Cosine regression analyses

For the cosine regression analyses, it was initially tested if the output from the original cosine regression models correlated with age by running Spearman correlations between the individual cosine amplitudes of each symptom from the original models and age, both for patients with PMDD and controls. No cosine amplitudes of any symptom except "Joint or muscle pain" for controls correlated significantly with age (ρ=-0.38, p=0.01), indicating that older controls have less cyclical variation for joint or muscle pain across the menstrual cycle. This may reflect age-related increases in baseline pain levels, resulting in less pronounced cyclical variation. Cosine amplitudes of all other symptoms did not correlate with age and therefore indicated that the cyclical variation did not differ across age.

Furthermore, all cosine regression models were estimated again adjusting for age, which showed that the results were very robust. Age was a significant covariate only for "Irritability" in the PMDD group (p=0.004), with older patients with PMDD showing slightly higher levels of irritability, however the effect size was negligible (Estimate=0.013, cosine amplitude range for irritability=-0.38 to 2.02). Distributions of cosine amplitudes yielded from the original model and the age adjusted model were nearly identical (comparing Figure 2 with Supplementary Figure 3), confirming that the results of the cosine regressions are robust to age adjustment.

LCGA analyses

For the LCGA analyses, Kruskal Wallis analyses for age across the three classes for each LCGA model were conducted, to test if age differed significantly between the original classes. No significant differences were found between the classes in each symptom and the total DRSP score. Sensitivity analyses were further conducted by including age as a covariate in all LCGA models, to see if the shapes of the temporal patterns changed when adjusting for age, and if class

membership differed between unadjusted and adjusted models. One patient was missing information on age, and therefore these comparisons include 89 patients instead of 90. Supplementary Table 12 shows that class assignments were highly consistent between original and age-adjusted models across most symptoms, with 14 out of 15 models exceeding 90% agreement (or close to as in the case of energy loss with 86.5% agreement). However, concentration difficulty showed lower agreement (58.4%), suggesting that age may have had a greater influence on temporal pattern solutions for this symptom specifically. Shapes of temporal patterns were virtually identical for all classes in 14 out of 15 models (comparing Figure 4 with Supplementary Figure 7), except for 'concentration difficulty' in which the age-adjusted model found a temporal pattern with a later offset of symptoms that was not found in the original solution (although including only 9% of participants).

CT-VAR analyses

For the CT-VAR analyses, scaled age was included as a time-independent predictor and the model was re-estimated with the four core symptoms of PMDD. From this model drift parameters estimated when keeping age constant at the mean (~35 years) were extracted. The original dynamic network and the age adjusted network followed the same key patterns (comparing Figure 6 with Supplementary Figure 10);

As in the original results, Depression went faster back to a low/no-symptom state than the other three core symptoms (more negative drift parameter estimate), and it had a dampening effect on the other symptoms, anxiety was a driver of the rate of change of all the other core symptoms with similar positive cross-symptom effects as in the original model. One difference noted between the age-adjusted model and the original model was that when adjusting for age, affective lability had a significant cross-symptom effect on the rate of change of depression and irritability, and irritability similarly had a significant cross-symptom effect on the rate of change of affective lability. Thus, affective lability was slightly more involved in the dynamic process compared to the original unadjusted model. Supplementary Table 14 further includes significant moderations of age, which should be interpreted as effects were age was a significant moderator of the effect. The positive effect of both anxiety and irritability on the rate of change of depression was estimated to be larger than the average estimate for older participants. On the contrary, the positive effect of

irritability on the rate of change of affective lability was estimated to be smaller than the average estimate for older participants. Finally, the rate of which affective lability and irritability returns to equilibrium was found to be slower for older participants.

SUPPLEMENTARY TABLES

Supplementary Table 1. Descriptive characteristics. The descriptive characteristics of patients with PMDD and controls. Most characteristics had zero, but maximum three missing values, except number of children that had six missing values for the PMDD group. Comparisons between patients with PMDD and controls on demographic variables were conducted using two-sided independent samples t-tests for continuous variables. For categorical variables two-sided χ2-tests were used, with two-sided Fisher's exact test applied for categorical variables with cell counts below 5. Patients with PMDD were significantly older and were more likely to have children than controls. Being married or cohabiting was significantly more common among patients with PMDD than controls, as well as being more likely to work full time, while controls were significantly more likely to be studying. Among patients with PMDD, there was a significantly higher proportion that did not attend university or college compared to controls.

	**PMDD**<br>**N = 90**	**Control**<br>**N = 44**	**p**
**Age (years)**			< 0.001
Mean (SD)	35 (6.0)	29 (7.0)	
Range	21, 46	20, 44	
**Ovulatory reproductive physiology**			-
Presence	100%	100%	
**Relationship status**			< 0.001
Married/Cohabiting	66 (73%)	11 (26%)	
Partner but living alone	10 (11%)	12 (28%)	
Single	14 (16%)	20 (47%)	
**Education**			< 0.001
Elementary school	3 (3.3%)	0 (0.0%)	
Gymnasium	21 (23%)	0 (0.0%)	
University/College	66 (73%)	43 (100%)	
**Employment**			< 0.001
Unemployed	2 (2.2%)	3 (7.0%)	
Parental leave	2 (2.2%)	0 (0.0%)	
Studying	7 (7.9%)	20 (47%)	
Working full time	60 (67%)	13 (30%)	
Working part time	18 (20%)	7 (16%)	
**Parity**			< 0.001
Nulliparous	35	40	
Parous	49	2	
**Menarche (years)**			0.48
Mean (SD)	13 (1.5)	13 (1.5)	
Range	8.0, 17	9.0, 15	
**Cycle length (days)**			0.28
Mean (SD)	28 (2.0)	29 (2.4)	
Range	24, 35	23, 35	
**Age of onset PMDD (years)**			-
Mean (SD)	24 (6.2)	-	

Range	12, 38	-	
**Past PMDD treatment**	71 (79%)	-	-
Hormonal	22 (24%)	-	
Hormonal pill	22 (24%)	-	
Other hormonal contraceptives	11 (12%)	-	
Progesterone cream	4 (4.4%)	-	
Homeopathy	13 (14%)	-	
Psychologist	8 (8.9%)	-	
SSRIs	58 (65%)	-	
Other	7 (7.8%)	-	

Supplementary Table 2. Average scores and standard deviations for the total DRSP score and each symptom. The average scores along with the standard deviation for the total DRSP score and for each PMDD symptom are presented separately for patients with PMDD and controls for the mid-follicular phase (day +7 to +11) and the premenstrual phase (day -5 to -1).

**Symptom**	**PMDD (m*ean (SD)*)**		**Controls (m*ean (SD)*)**	
	**Mid-follicular**	**Premenstrual**	**Mid-follicular**	**Premenstrual**
Total DRSP	29 (8.6)	69 (19)	26 (6.2)	28 (6.7)
Depression	1.4 (0.44)	3.3 (1.1)	1.2 (0.36)	1.2 (0.36)
Anxiety	1.4 (0.49)	3.5 (1.2)	1.4 (0.57)	1.4 (0.57)
Affective lability	1.4 (0.50)	3.7 (1.1)	1.2 (0.28)	1.3 (0.37)
Irritability	1.4 (0.52)	3.5 (1.1)	1.1 (0.20)	1.3 (0.38)
Anhedonia	1.4 (0.52)	3.7 (1.1)	1.3 (0.38)	1.3 (0.45)
Concentration difficulties	1.5 (0.63)	3.4 (1.2)	1.3 (0.46)	1.4 (0.51)
Energy loss	1.8 (0.79)	3.9 (1.1)	1.7 (0.67)	1.7 (0.65)
Appetite disturbances	1.3 (0.42)	3.1 (1.3)	1.2 (0.38)	1.4 (0.55)
Sleep disturbances	1.5 (0.57)	3.1 (1.1)	1.4 (0.54)	1.4 (0.52)
Overwhelmed	1.3 (0.45)	3.0 (1.2)	1.2 (0.40)	1.2 (0.39)
Breast tenderness	1.1 (0.22)	2.9 (1.4)	1.1 (0.30)	1.3 (0.76)
Breast swelling /bloating/weight gain	1.2 (0.44)	3.5 (1.4)	1.1 (0.33)	1.3 (0.61)
Joint or muscle pain	1.4 (0.78)	2.6 (1.3)	1.2 (0.55)	1.2 (0.53)
Headache	1.4 (0.63)	2.2 (1.1)	1.3 (0.58)	1.2 (0.40)

Supplementary Table 3. Correlation of cosine amplitudes. The table includes correlation coefficients along with degrees of freedom (df), Benjamini-Hochberg-adjusted p-values, and 95% confidence intervals for all pairwise correlations between the cosine amplitudes for each PMDD symptom, in both patients with PMDD and controls. P-values marked in bold are significant.

	PMDD			Controls		
	r(df)	p (adjusted)	95% CI [lower, upper]	r(df)	p (adjusted)	95% CI [lower, upper]
Depression-Anxiety	0.90(88)	< **0.001**	[0.81, 0.95]	0.52(42)	**0.002**	[0.03, 0.81]
Depression-Affective lability	0.90(88)	< **0.001**	[0.79, 0.95]	0.43(42)	**0.013**	[-0.08, 0.76]
Depression-Irritability	0.75(88)	< **0.001**	[0.54, 0.87]	0.34(42)	0.070	[-0.18, 0.71]
Depression-Anhedonia	0.83(88)	< **0.001**	[0.68, 0.92]	0.29(42)	0.140	[-0.24, 0.68]
Depression-Concentration	0.85(88)	< **0.001**	[0.72, 0.93]	0.30(42)	0.122	[-0.23, 0.69]
Depression-Energy loss	0.73(88)	< **0.001**	[0.50, 0.86]	0.08(42)	0.717	[-0.43, 0.55]
Depression-Appetite	0.57(88)	< **0.001**	[0.28, 0.77]	0.14(42)	0.549	[-0.38, 0.59]
Depression-Sleep	0.68(88)	< **0.001**	[0.43, 0.84]	0.39(42)	**0.034**	[-0.13, 0.74]
Depression-Overwhelmed	0.87(88)	< **0.001**	[0.74, 0.93]	0.22(42)	0.310	[-0.31, 0.64]
Depression-Breast tenderness	0.37(88)	< **0.001**	[0.02, 0.64]	0.21(42)	0.310	[-0.31, 0.64]
Depression-Breast swelling/bloating/weight gain	0.54(88)	< **0.001**	[0.23, 0.75]	0.13(42)	0.560	[-0.39, 0.59]
Depression-Joint or muscle pain	0.50(88)	< **0.001**	[0.18, 0.72]	0.27(42)	0.170	[-0.25, 0.67]
Depression-Headache	0.45(88)	< **0.001**	[0.12, 0.70]	0.04(42)	0.891	[-0.46, 0.52]
Anxiety-Affective lability	0.88(88)	< **0.001**	[0.77, 0.94]	0.48(42)	**0.005**	[-0.02, 0.79]
Anxiety-Irritability	0.73(88)	< **0.001**	[0.50, 0.86]	0.31(42)	0.108	[-0.22, 0.70]
Anxiety-Anhedonia	0.81(88)	< **0.001**	[0.64, 0.90]	0.43(42)	**0.013**	[-0.08, 0.76]
Anxiety-Concentration	0.81(88)	< **0.001**	[0.64, 0.90]	-0.03(42)	0.900	[-0.52, 0.47]
Anxiety-Energy loss	0.71(88)	< **0.001**	[0.48, 0.85]	0.16(42)	0.490	[-0.36, 0.61]

Anxiety-Appetite	0.44(88)	**< 0.001**	[0.11, 0.69]	0.09(42)	0.694	[-0.42, 0.56]
Anxiety-Sleep	0.67(88)	**< 0.001**	[0.42, 0.83]	0.09(42)	0.694	[-0.42, 0.56]
Anxiety-Overwhelmed	0.81(88)	**< 0.001**	[0.64, 0.90]	0.59(42)	**< 0.001**	[0.14, 0.84]
Anxiety-Breast tenderness	0.40(88)	**< 0.001**	[0.06, 0.66]	0.04(42)	0.891	[-0.46, 0.52]
Anxiety-Breast swelling/bloating/weight gain	0.53(88)	**< 0.001**	[0.21, 0.74]	0.09(42)	0.694	[-0.42, 0.56]
Anxiety-Joint or muscle pain	0.51(88)	**< 0.001**	[0.19, 0.73]	-0.13(42)	0.560	[-0.59, 0.39]
Anxiety-Headache	0.42(88)	**< 0.001**	[0.07, 0.67]	-0.02(42)	0.925	[-0.51, 0.48]
Affective lability-Irritability	0.79(88)	**< 0.001**	[0.60, 0.89]	0.68(42)	**< 0.001**	[0.28, 0.88]
Affective lability-Anhedonia	0.81(88)	**< 0.001**	[0.64, 0.90]	0.73(42)	**< 0.001**	[0.37, 0.90]
Affective lability-Concentration	0.85(88)	**< 0.001**	[0.71, 0.93]	0.12(42)	0.592	[-0.40, 0.58]
Affective lability-Energy loss	0.71(88)	**< 0.001**	[0.47, 0.85]	0.58(42)	**< 0.001**	[0.13, 0.84]
Affective lability-Appetite	0.57(88)	**< 0.001**	[0.26, 0.77]	0.45(42)	**0.009**	[-0.05, 0.77]
Affective lability-Sleep	0.70(88)	**< 0.001**	[0.46, 0.85]	-0.03(42)	0.900	[-0.52, 0.47]
Affective lability-Overwhelmed	0.80(88)	**< 0.001**	[0.62, 0.90]	0.26(42)	0.190	[-0.26, 0.67]
Affective lability-Breast tenderness	0.44(88)	**< 0.001**	[0.10, 0.69]	0.35(42)	0.065	[-0.17, 0.72]
Affective lability-Breast swelling/Bloating/Weight gain	0.55(88)	**< 0.001**	[0.24, 0.76]	0.48(42)	**0.005**	[-0.02, 0.79]
Affective lability-Joint or muscle pain	0.54(88)	**< 0.001**	[0.22, 0.75]	0.39(42)	**0.030**	[-0.12, 0.74]
Affective lability-Headache	0.46(88)	**< 0.001**	[0.13, 0.70]	0.04(42)	0.891	[-0.46, 0.52]
Irritability-Anhedonia	0.83(88)	**< 0.001**	[0.68, 0.92]	0.66(42)	**< 0.001**	[0.25, 0.87]
Irritability-Concentration	0.76(88)	**< 0.001**	[0.55, 0.88]	0.15(42)	0.522	[-0.37, 0.60]
Irritability-Energy loss	0.66(88)	**< 0.001**	[0.39, 0.82]	0.56(42)	**0.001**	[0.10, 0.83]
Irritability-Appetite	0.54(88)	**< 0.001**	[0.23, 0.75]	0.46(42)	**0.008**	[-0.05, 0.78]

Irritability-Sleep	0.58(88)	**< 0.001**	[0.28, 0.77]	0.15(42)	0.529	[-0.37, 0.60]
Irritability-Overwhelmed	0.75(88)	**< 0.001**	[0.54, 0.87]	0.07(42)	0.789	[-0.44, 0.54]
Irritability-Breast tenderness	0.41(88)	**< 0.001**	[0.06, 0.67]	0.14(42)	0.548	[-0.37, 0.59]
Irritability-Breast swelling/Bloating/Weight gain	0.46(88)	**< 0.001**	[0.13, 0.70]	0.33(42)	0.085	[-0.20, 0.71]
Irritability-Joint or muscle pain	0.48(88)	**< 0.001**	[0.16, 0.72]	0.57(42)	**< 0.001**	[0.11, 0.83]
Irritability-Headache	0.49(88)	**< 0.001**	[0.16, 0.72]	0.21(42)	0.310	[-0.31, 0.64]
Anhedonia-Concentration	0.83(88)	**< 0.001**	[0.68, 0.92]	0.13(42)	0.560	[-0.38, 0.59]
Anhedonia-Energy loss	0.77(88)	**< 0.001**	[0.58, 0.88]	0.74(42)	**< 0.001**	[0.39, 0.90]
Anhedonia-Appetite	0.56(88)	**< 0.001**	[0.26, 0.77]	0.52(42)	**0.002**	[0.03, 0.80]
Anhedonia-Sleep	0.65(88)	**< 0.001**	[0.38, 0.81]	-0.07(42)	0.785	[-0.54, 0.44]
Anhedonia-Overwhelmed	0.79(88)	**< 0.001**	[0.61, 0.90]	0.37(42)	**0.049**	[-0.15, 0.73]
Anhedonia-Breast tenderness	0.43(88)	**< 0.001**	[0.09, 0.68]	0.27(42)	0.170	[-0.25, 0.68]
Anhedonia-Breast swelling/Bloating/Weight gain	0.56(88)	**< 0.001**	[0.26, 0.76]	0.48(42)	**0.005**	[-0.01, 0.79]
Anhedonia-Joint or muscle pain	0.46(88)	**< 0.001**	[0.13, 0.70]	0.25(42)	0.213	[-0.28, 0.66]
Anhedonia-Headache	0.50(88)	**< 0.001**	[0.18, 0.73]	-0.03(42)	0.900	[-0.52, 0.47]
Concentration-Energy loss	0.73(88)	**< 0.001**	[0.51, 0.86]	0.20(42)	0.335	[-0.32, 0.63]
Concentration-Appetite	0.65(88)	**< 0.001**	[0.39, 0.82]	-0.04(42)	0.900	[-0.52, 0.47]
Concentration-Sleep	0.70(88)	**< 0.001**	[0.45, 0.84]	0.35(42)	0.064	[-0.17, 0.72]
Concentration-Overwhelmed	0.78(88)	**< 0.001**	[0.59, 0.89]	-0.01(42)	0.938	[-0.50, 0.48]
Concentration-Breast tenderness	0.47(88)	**< 0.001**	[0.13, 0.70]	0.11(42)	0.618	[-0.40, 0.57]
Concentration-Breast swelling/Bloating/Weight gain	0.59(88)	**< 0.001**	[0.30, 0.78]	-0.02(42)	0.911	[-0.51, 0.47]

Concentration-Joint or muscle pain	0.56(88)	**< 0.001**	[0.26, 0.76]	0.13(42)	0.581	[-0.39, 0.58]
Concentration-Headache	0.51(88)	**< 0.001**	[0.19, 0.73]	0.20(42)	0.347	[-0.33, 0.63]
Energy loss-Appetite	0.57(88)	**< 0.001**	[0.27, 0.77]	0.71(42)	**< 0.001**	[0.33, 0.89]
Energy loss-Sleep	0.78(88)	**< 0.001**	[0.59, 0.89]	-0.12(42)	0.581	[-0.58, 0.39]
Energy loss-Overwhelmed	0.74(88)	**< 0.001**	[0.52, 0.87]	0.23(42)	0.269	[-0.30, 0.65]
Energy loss-Breast tenderness	0.40(88)	**< 0.001**	[0.05, 0.66]	0.49(42)	**0.004**	[-0.01, 0.79]
Energy loss-Breast swelling/Bloating/Weight gain	0.56(88)	**< 0.001**	[0.26, 0.76]	0.55(42)	**0.001**	[0.08, 0.82]
Energy loss-Joint or muscle pain	0.54(88)	**< 0.001**	[0.23, 0.75]	0.37(42)	**0.049**	[-0.15, 0.73]
Energy loss-Headache	0.54(88)	**< 0.001**	[0.23, 0.75]	0.06(42)	0.794	[-0.44, 0.54]
Appetite-Sleep	0.64(88)	**< 0.001**	[0.37, 0.81]	-0.25(42)	0.213	[-0.66, 0.28]
Appetite-Overwhelmed	0.53(88)	**< 0.001**	[0.22, 0.75]	0.13(42)	0.560	[-0.39, 0.59]
Appetite-Breast tenderness	0.43(88)	**< 0.001**	[0.09, 0.68]	0.34(42)	0.069	[-0.18, 0.71]
Appetite-Breast swelling/Bloating/Weight gain	0.65(88)	**< 0.001**	[0.38, 0.81]	0.50(42)	**0.003**	[0.01, 0.80]
Appetite-Joint or muscle pain	0.47(88)	**< 0.001**	[0.14, 0.71]	0.54(42)	**0.001**	[0.06, 0.82]
Appetite-Headache	0.49(88)	**< 0.001**	[0.17, 0.72]	-0.24(42)	0.227	[-0.66, 0.28]
Sleep-Overwhelmed	0.69(88)	**< 0.001**	[0.44, 0.84]	-0.21(42)	0.310	[-0.64, 0.31]
Sleep-Breast tenderness	0.50(88)	**< 0.001**	[0.17, 0.72]	0.10(42)	0.665	[-0.41, 0.57]
Sleep-Breast swelling/Bloating/Weight gain	0.60(88)	**< 0.001**	[0.31, 0.79]	0.00(42)	0.990	[-0.49, 0.49]
Sleep-Joint or muscle pain	0.64(88)	**< 0.001**	[0.37, 0.81]	0.02(42)	0.911	[-0.47, 0.51]
Sleep-Headache	0.57(88)	**< 0.001**	[0.28, 0.77]	0.32(42)	0.097	[-0.21, 0.70]
Overwhelmed-Breast tenderness	0.42(88)	**< 0.001**	[0.07, 0.67]	0.02(42)	0.911	[-0.47, 0.51]

Overwhelmed-Breast swelling/Bloating/Weight gain	0.51(88)	**< 0.001**	[0.18, 0.73]	0.18(42)	0.426	[-0.35, 0.62]
Overwhelmed-Joint or muscle pain	0.55(88)	**< 0.001**	[0.25, 0.76]	-0.16(42)	0.484	[-0.61, 0.36]
Overwhelmed-Headache	0.60(88)	**< 0.001**	[0.32, 0.79]	0.11(42)	0.618	[-0.40, 0.57]
Breast tenderness-Breast swelling/Bloating/Weight gain	0.74(88)	**< 0.001**	[0.52, 0.86]	0.81(42)	**< 0.001**	[0.53, 0.93]
Breast tenderness-Joint or muscle pain	0.48(88)	**< 0.001**	[0.15, 0.71]	0.18(42)	0.426	[-0.34, 0.62]
Breast tenderness-Headache	0.38(88)	**< 0.001**	[0.03, 0.65]	0.25(42)	0.213	[-0.27, 0.66]
Breast swelling/Bloating/Weight gain-Joint or muscle pain	0.54(88)	**< 0.001**	[0.23, 0.75]	0.32(42)	0.091	[-0.20, 0.70]
Breast swelling/Bloating/Weight gain-Headache	0.40(88)	**< 0.001**	[0.05, 0.66]	0.10(42)	0.683	[-0.42, 0.56]
Joint or muscle pain-Headache	0.51(88)	**< 0.001**	[0.19, 0.73]	-0.14(42)	0.560	[-0.59, 0.38]

Supplementary Table 4. Effect sizes explaining the variance between and within patients with PMDD. Effect sizes of fixed and random effects of the cosine effect of time for each symptom are presented for patients with PMDD across three Linear Mixed Effects models estimated with the PACTS scaled time variable. Overall variance is attributed to individual differences in mean level (model 1), average menstrual cycle effect (model 2), and individual differences in menstrual cycle effect (final model). Model 1 included only random intercepts and effect sizes were computed using the intraclass correlation (ICC). These effects were significant for all symptoms in patients with PMDD, reflecting variance explained by average symptom levels across individuals. Values closer to zero indicate greater within-person variance in average symptom levels. Model 2 included fixed effects of the cosine function applied on the menstrual cycle, and was significant for all symptoms in patients with PMDD. Effect sizes for model 2 were defined as the percentual residual change between model 1 and 2, and should be interpreted as how much of the variance that is explained by the average effect of the cosine function applied on the menstrual cycle. The final model included both fixed and random effects of the cosine function applied on the menstrual cycle, and the random effects were significant for all symptoms in patients with PMDD. The effect sizes for the final model were defined as the percentual residual change between model 1 and the final model, and should be interpreted as how much of the variance that is explained by individual differences in the cosine function applied on the menstrual cycle. For example, in patients with PMDD, variance explained by average levels of symptoms for depression was 0.17, variance explained by the cosine effect on the menstrual cycle was 18%, and variance explained by individual differences in the cosine function applied on the menstrual cycle was 25%. P-values for the ICC (model 1) and the random effects (final model) were derived using one-sided likelihood ratio tests, while p-values for the fixed effects (model 2) were derived using two-sided t-tests with Satterthwaite's method. P-values for ICC values, and fixed and random effects were corrected for multiple testing using Bonferroni correction. 95% confidence intervals for fixed and random effects were obtained using non-parametric bootstrapping with 1000 iterations.

**Symptom**	**Random intercepts (Model 1)**		**Fixed effects of cycle (Model 2)**		**Random effects of cycle (Final model)**	
	**ICC (p adjusted)**	**95% CI [lower, higher]**	**% variance explained (p adjusted)**	**95% CI [lower, higher]**	**% variance explained (p adjusted)**	**95% CI [lower, higher]**
Depression	0.17 ($p < 0.001$)	[0.12, 0.23]	18 % ($p < 0.001$)	[14%, 22%]	25 % ($p < 0.001$)	[21%, 30%]
Anxiety	0.17 ($p < 0.001$)	[0.12, 0.23]	13 % ($p < 0.001$)	[9.6%, 17%]	21 % ($p < 0.001$)	[17%, 26%]
Affective lability	0.15 ($p < 0.001$)	[0.09, 0.20]	17 % ($p < 0.001$)	[14%, 21%]	24 % ($p < 0.001$)	[20%, 28%]
Irritability	0.15 ($p < 0.001$)	[0.11, 0.21]	14 % ($p < 0.001$)	[9.5%, 18%]	21 % ($p < 0.001$)	[17%, 25%]
Anhedonia	0.13 ($p < 0.001$)	[0.09, 0.18]	16 % ($p < 0.001$)	[12%, 21%]	23 % ($p < 0.001$)	[19%, 27%]
Concentration	0.20 ($p < 0.001$)	[0.14, 0.26]	14 % ($p < 0.001$)	[11%, 18%]	21 % ($p < 0.001$)	[17%, 26%]
Energy loss	0.16 ($p < 0.001$)	[0.12, 0.21]	15 % ($p < 0.001$)	[12%, 19%]	21 % ($p < 0.001$)	[17%, 26%]

Appetite	0.25 (p < 0.001)	[0.18, 0.33]	16 % (p < 0.001)	[12%, 20%]	24 % (p < 0.001)	[19%, 29%]
Sleep	0.24 (p < 0.001)	[0.18, 0.32]	15 % (p < 0.001)	[11%, 18%]	21 % (p < 0.001)	[16%, 27%]
Overwhelmed	0.20 (p < 0.001)	[0.15, 0.28]	16 % (p < 0.001)	[12%, 20%]	24 % (p < 0.001)	[20%, 29%]
Breast tenderness	0.22 (p < 0.001)	[0.16, 0.27]	18 % (p < 0.001)	[14%, 23%]	32 % (p < 0.001)	[26%, 38%]
Breast swelling/ Bloating/ Weight gain	0.19 (p < 0.001)	[0.12, 0.25]	23 % (p < 0.001)	[18%, 28%]	33 % (p < 0.001)	[29%, 39%]
Joint or muscle pain	0.36 (p < 0.001)	[0.27, 0.42]	9.5 % (p < 0.001)	[6.1%, 13%]	22 % (p < 0.001)	[17%, 28%]
Headache	0.27 (p < 0.001)	[0.19, 0.34]	6.3 % (p < 0.001)	[4.3%, 8.9%]	12 % (p < 0.001)	[9.2%, 16%]

Supplementary Table 5. Effect sizes explaining the variance between and within controls. Effect sizes of fixed and random effects of the cosine effect of time for each symptom are presented for controls across three Linear Mixed Effects models estimated with the PACTS scaled time variable. Overall variance is attributed to individual differences in mean level (model 1), average menstrual cycle effect (model 2), and individual differences in menstrual cycle effect (final model). Model 1 included only random intercepts and effect sizes were computed using the intraclass correlation (ICC). These effects were significant for all symptoms in controls, reflecting variance explained by average symptom levels across individuals. Values closer to zero indicate greater within-person variance in average symptom levels. Model 2 included fixed effects of the cosine function applied on the menstrual cycle, and was significant for about half of the symptoms in controls, although the effects were minor. Effect sizes for model 2 were defined as the percentual residual change between model 1 and 2, and should be interpreted as how much of the variance that is explained by the average effect of the cosine function applied on the menstrual cycle. The final model included both fixed and random effects of the cosine function applied on the menstrual cycle, and the random effects were significant for the majority of symptoms for controls, although the effects were considerably small. The effect sizes for the final model were defined as the percentual residual change between model 1 and the final model, and should be interpreted as how much of the variance that is explained by individual differences in the cosine function applied on the menstrual cycle. P-values for the ICC (model 1) and the random effects (final model) were derived using one-sided likelihood ratio tests, while p-values for the fixed effects (model 2) were derived using two-sided t-tests with Satterthwaite's method. P-values for ICC values, and fixed and random effects were corrected for multiple testing using Bonferroni correction. 95% confidence intervals for fixed and random effects were obtained using non-parametric bootstrapping with 1000 iterations.

**Symptom**	**Random intercepts (Model 1)**		**Fixed effects of cycle (Model 2)**		**Random effects of cycle (Final model)**	
	**ICC (p adjusted)**	**95% CI [lower, higher]**	**% variance explained (p adjusted)**	**95% CI [lower, higher]**	**% variance explained (p adjusted)**	**95% CI [lower, higher]**
Depression	0.38 (p < 0.001)	[0.24, 0.50]	0.04 % (p = 1.00)	[0.00001%, 0.69%]	3.0 % (p = 0.039)	[0.05%, 6.3%]
Anxiety	0.41 (p < 0.001)	[0.29, 0.54]	0.11 % (p = 1.00)	[0.0002%, 0.80%]	1.3 % (p = 1.00)	[0.01%, 3.3%]
Affective lability	0.22 (p < 0.001)	[0.14, 0.31]	0.89 % (p < 0.001)	[0.11%, 2.4%]	4.9 % (p < 0.001)	[1.6%, 7.6%]
Irritability	0.21 (p < 0.001)	[0.14, 0.33]	0.70 % (p = 0.002)	[0.04%, 2.0%]	5.0 % (p < 0.001)	[2.4%, 6.7%]
Anhedonia	0.25 (p < 0.001)	[0.14, 0.40]	0.18 % (p = 0.809)	[0.0007%, 0.91%]	2.0 % (p = 0.038)	[0.11%, 4,1%]
Concentration	0.36 (p < 0.001)	[0.19, 0.46]	0.22 % (p = 0.503)	[0.002%, 1.1%]	2.3 % (p = 0.478)	[0.26%, 4.5%]
Energy loss	0.28 (p < 0.001)	[0.20, 0.39]	0.61 % (p = 0.006)	[0.09%, 1.6%]	1.8 % (p = 1.00)	[0.55%, 3.5%]
Appetite	0.37 (p < 0.001)	[0.22, 0.48]	3.6 % (p < 0.001)	[1.9%, 5.9%]	8.9 % (p < 0.001)	[4.0%, 14%]

Sleep	0.38 (p < 0.001)	[0.22, 0.50]	0.04 % (p = 1.00)	[0.0001%, 0.67%]	2.5 % (p = 0.025)	[0.77%, 3.8%]
Overwhelmed	0.29 (p < 0.001)	[0.16, 0.42]	0.09 % (p = 1.00)	[0.0004%, 0.64%]	1.4 % (p = 0.130)	[0.03%, 3.4%]
Breast tenderness	0.41 (p < 0.001)	[0.26, 0.52]	5.3 % (p < 0.001)	[2.2%, 9.3%]	24 % (p < 0.001)	[12%, 31%]
Breast swelling/ Bloating/ Weight gain	0.27 (p < 0.001)	[0.15, 0.36]	7.2 % (p < 0.001)	[4.0%, 11%]	19 % (p < 0.001)	[10%, 26%]
Joint or muscle pain	0.49 (p < 0.001)	[0.38, 0.64]	0.34 % (p = 0.125)	[0.005%, 1.5%]	4.4 % (p = 0.002)	[0.13%, 10%]
Headache	0.37 (p < 0.001)	[0.26, 0.50]	0.17 % (p = 0.847)	[0.0006%, 1.1%]	4.0 % (p = 0.004)	[0.91%, 6.2%]

Supplementary Table 6. Effect sizes explaining the variance between and within patients with PMDD (uniformly scaled time variable). Effect sizes of the fixed and random effects of the cosine effect of time for each symptom are presented for patients with PMDD across the three Linear Mixed Effects models estimated with the uniformly scaled time variable. Overall variance is attributed to individual differences in mean level (model 1), average menstrual cycle effect (model 2), and individual differences in menstrual cycle effect (final model). Model 1 included only random intercepts and effect sizes for this model were computed using the intraclass correlation (ICC). These effects were significant for all symptoms in patients with PMDD, reflecting variance explained by average symptom levels across individuals. Values closer to zero indicate greater within-person variance in average symptom levels. Model 2 included fixed effects of the cosine function applied on the menstrual cycle, and was significant for all symptoms in patients with PMDD. Effect sizes for model 2 were defined as the percentual residual change between model 1 and 2, and should be interpreted as how much of the variance that is explained by the average effect of the cosine function applied on the menstrual cycle. The final model included both fixed and random effects of the cosine function applied on the menstrual cycle, and the random effects were significant for all symptoms in patients with PMDD. The effect sizes for the final model were defined as the percentual residual change between model 1 and the final model, and should be interpreted as how much of the variance that is explained by individual differences in the cosine function applied on the menstrual cycle. P-values for the ICC (model 1) and the random effects (final model) were derived using one-sided likelihood ratio tests, while p-values for the fixed effects (model 2) were derived using two-sided t-tests with Satterthwaite's method. P-values for ICC values, and fixed and random effects were corrected for multiple testing using Bonferroni correction. 95% confidence intervals for fixed and random effects were obtained using non-parametric bootstrapping with 1000 iterations.

**Symptom**	**Random intercepts (Model 1)**		**Fixed effects of cycle (Model 2)**		**Random effects of cycle (Final model)**	
	**ICC (p adjusted)**	**95% CI [lower, higher]**	**% variance explained (p adjusted)**	**95% CI [lower, higher]**	**% variance explained (p adjusted)**	**95% CI [lower, higher]**
Depression	0.17 ($p < 0.001$)	[0.11, 0.23]	18% ($p < 0.001$)	[14%, 22%]	25% ($p < 0.001$)	[20%, 30%]
Anxiety	0.17 ($p < 0.001$)	[0.12, 0.23]	13% ($p < 0.001$)	[9.4%, 17%]	21% ($p < 0.001$)	[17%, 26%]
Affective lability	0.15 ($p < 0.001$)	[0.11, 0.20]	17% ($p < 0.001$)	[14%, 21%]	23% ($p < 0.001$)	[20%, 28%]
Irritability	0.15 ($p < 0.001$)	[0.10, 0.21]	14% ($p < 0.001$)	[10%, 18%]	21% ($p < 0.001$)	[17%, 25%]
Anhedonia	0.13 ($p < 0.001$)	[0.09, 0.18]	16% ($p < 0.001$)	[13%, 20%]	23% ($p < 0.001$)	[19%, 27%]
Concentration	0.20 ($p < 0.001$)	[0.15, 0.26]	14% ($p < 0.001$)	[11%, 18%]	21% ($p < 0.001$)	[17%, 25%]
Energy loss	0.16 ($p < 0.001$)	[0.12, 0.23]	15% ($p < 0.001$)	[12%, 19%]	22% ($p < 0.001$)	[18%, 26%]
Appetite	0.25 ($p < 0.001$)	[0.19, 0.34]	16% ($p < 0.001$)	[13%, 20%]	24% ($p < 0.001$)	[20%, 30%]

Sleep	0.24 (p < 0.001)	[0.16, 0.30]	14% (p < 0.001)	[11%, 18%]	22% (p < 0.001)	[17%, 28%]
Overwhelmed	0.20 (p < 0.001)	[0.12, 0.26]	15% (p < 0.001)	[12%, 19%]	24% (p < 0.001)	[19%, 29%]
Breast tenderness	0.22 (p < 0.001)	[0.16, 0.29]	19% (p < 0.001)	[14%, 23%]	32% (p < 0.001)	[26%, 38%]
Breast swelling/ Bloating/ Weight gain	0.19 (p < 0.001)	[0.12, 0.25]	24% (p < 0.001)	[19%, 29%]	34% (p < 0.001)	[29%, 39%]
Joint or muscle pain	0.36 (p < 0.001)	[0.25, 0.46]	9.8% (p < 0.001)	[6.1%, 14%]	23% (p < 0.001)	[17%, 29%]
Headache	0.27 (p < 0.001)	[0.20, 0.34]	6.6% (p < 0.001)	[4.4%, 9.0%]	12% (p < 0.001)	[9.4%, 16%]

Supplementary Table 7. Effect sizes explaining the variance between and within controls (uniformly scaled time variable). Effect sizes of the fixed and random effects of the cosine effect of time for each symptom are presented for controls across the three Linear Mixed Effects models estimated with the uniformly scaled time variable. Overall variance is attributed to individual differences in mean level (model 1), average menstrual cycle effect (model 2), and individual differences in menstrual cycle effect (final model). Model 1 included only random intercepts and effect sizes for this model were computed using the intraclass correlation (ICC). These effects were significant for all symptoms in controls, reflecting variance explained by average symptom levels across individuals. Values closer to zero indicate greater within-person variance in average symptom levels. Model 2 included fixed effects of the cosine function applied on the menstrual cycle, and was significant for about half of the symptoms in controls, although the effects were minor. Effect sizes for model 2 were defined as the percentual residual change between model 1 and 2, and should be interpreted as how much of the variance that is explained by the average effect of the cosine function applied on the menstrual cycle. The final model included both fixed and random effects of the cosine function applied on the menstrual cycle, and the random effects were significant for the majority of symptoms for the models based on controls, although considerably small. The effect sizes for the final model were defined as the percentual residual change between model 1 and the final model, and should be interpreted as how much of the variance that is explained by individual differences in the cosine function applied on the menstrual cycle. P-values for the ICC (model 1) and the random effects (final model) were derived using one-sided likelihood ratio tests, while p-values for the fixed effects (model 2) were derived using two-sided t-tests with Satterthwaite's method. P-values for ICC values, and fixed and random effects were corrected for multiple testing using Bonferroni correction. 95% confidence intervals for fixed and random effects were obtained using non-parametric bootstrapping with 1000 iterations.

**Symptom**	**Random intercepts (Model 1)**		**Fixed effects of cycle (Model 2)**		**Random effects of cycle (Final model)**	
	**ICC (p adjusted)**	**95% CI [lower, higher]**	**% variance explained (p adjusted)**	**95% CI [lower, higher]**	**% variance explained (p adjusted)**	**95% CI [lower, higher]**
Depression	0.38 (p < 0.001)	[0.24, 0.47]	0.03% (p = 1.00)	[0.00008 %, 0.68%]	3.1% (p = 0.020)	[0.26%, 7.4%]
Anxiety	0.41 (p < 0.001)	[0.29, 0.55]	0.14% (p = 1.00)	[0.0004% , 0.81%]	1.1% (p = 1.00)	[0.02%, 5.4%]
Affective lability	0.22 (p < 0.001)	[0.12, 0.34]	0.68% (p = 0.003)	[0.03%, 2.2%]	5.2% (p < 0.001)	[2.3%, 9.0%]
Irritability	0.21 (p < 0.001)	[0.12, 0.32]	0.67% (p = 0.003)	[0.04%, 1.9%]	4.8% (p < 0.001)	[3.2%, 7.6%]
Anhedonia	0.25 (p < 0.001)	[0.16, 0.37]	0.16% (p = 1.00)	[0.0003% , 0.86%]	2.1% (p = 0.017)	[0.82%, 5.4%]
Concentration	0.36 (p < 0.001)	[0.22, 0.48]	0.38% (p = 0.073)	[0.009%, 1.4%]	2.7% (p = 0.230)	[0.89%, 5.5%]

Energy loss	0.28 (p < 0.001)	[0.16, 0.38]	0.57% (p = 0.009)	[0.06%, 1.6%]	2.0% (p = 0.596)	[0.99%, 5.3%]
Appetite	0.37 (p < 0.001)	[0.23, 0.47]	3.9% (p < 0.001)	[2.1%, 6.2%]	9.6% (p < 0.001)	[5.0%, 17%]
Sleep	0.38 (p < 0.001)	[0.22, 0.46]	0.09% (p = 1.00)	[0.0002%, 0.79%]	2.4% (p = 0.071)	[1.6%, 4.7%]
Overwhelmed	0.29 (p < 0.001)	[0.15, 0.41]	0.07% (p = 1.00)	[0.00004%, 0.68%]	1.2% (p = 1.00)	[0.07%, 5.4%]
Breast tenderness	0.41 (p < 0.001)	[0.22, 0.52]	4.5% (p < 0.001)	[2.0%, 8.0%]	18% (p < 0.001)	[9.8%, 28%]
Breast swelling/ Bloating/ Weight gain	0.27 (p < 0.001)	[0.15, 0.37]	6.6% (p < 0.001)	[3.7%, 9.9%]	15% (p < 0.001)	[9.9%, 23%]
Joint or muscle pain	0.49 (p < 0.001)	[0.38, 0.65]	0.34% (p = 0.127)	[0.004%, 1.5%]	5.7% (p < 0.001)	[1.4%, 13%]
Headache	0.37 (p < 0.001)	[0.23, 0.54]	0.18% (p = 0.781)	[0.0009%, 1.1%]	3.8% (p = 0.010)	[1.6%, 7.7%]

Supplementary Table 8. Model output of cosine regression model including the interaction between time and group. The table presents the estimates, standard errors (SE), 95% confidence intervals (CI), and Bonferroni adjusted p-values for the Time by Group interactions for each symptom, reflecting whether cyclical symptom patterns differed significantly between patients with PMDD and controls for each symptom. P-values for the interaction effects were derived using two-sided t-tests with Satterthwaite's method, and 95% CIs were estimated using the Wald method based on Satterthwaite-approximated degrees of freedom. All interaction terms were statistically significant.

**Symptom**	**Estimate**	**SE**	**95% CI [lower, higher]**	p_{adj}
Depression	0.77	0.079	[0.61, 0.92]	<0.001
Anxiety	0.71	0.090	[0.53, 0.89]	<0.001
Affective lability	0.80	0.083	[0.63, 0.96]	<0.001
Irritability	0.69	0.086	[0.52, 0.86]	<0.001
Anhedonia	0.83	0.087	[0.65, 1.00]	<0.001
Concentration	0.73	0.085	[0.56, 0.89]	<0.001
Energy loss	0.75	0.087	[0.58, 0.92]	<0.001
Appetite	0.58	0.085	[0.41, 0.74]	<0.001
Sleep	0.62	0.072	[0.48, 0.77]	<0.001
Overwhelmed	0.67	0.081	[0.51, 0.83]	<0.001
Breast tenderness	0.56	0.10	[0.36, 0.76]	<0.001
Breast swelling/ Bloating/Weight gain	0.74	0.11	[0.53, 0.95]	<0.001
Joint or muscle pain	0.42	0.088	[0.25, 0.60]	<0.001
Headache	0.34	0.064	[0.21, 0.46]	<0.001

Supplementary Table 9. Total explained variance and proportions of fixed and random effects of cyclic time centered around menses onset (GAMM). The total explained variance (R^2 adj) of the GAMM models for each PMDD symptom estimated with PACTS scaled cyclic time centered around menses onset. The total explained variance is further partitioned into proportions of fixed and random effects, presented as percentages.

	**R^2 (adj)**		**Fixed effect (%)**		**Random effect (%)**	
**Symptom**	**PMDD**	**Controls**	**PMDD**	**Controls**	**PMDD**	**Controls**
Depression	0.29	0.35	79.1	0.0	20.9	100
Anxiety	0.28	0.32	74.1	0.30	25.9	99.7
Affective lability	0.32	0.18	78.8	5.40	21.2	94.6
Irritability	0.32	0.12	71.6	6.80	28.4	93.2
Anhedonia	0.28	0.19	78.3	0.70	21.7	99.3
Concentration	0.26	0.32	70.3	0.80	29.7	99.2
Energy loss	0.23	0.16	77.7	3.0	22.3	97.0
Appetite	0.25	0.27	69.8	9.30	30.2	90.7
Sleep	0.22	0.21	69.5	0.0	30.5	100
Overwhelmed	0.25	0.15	72.0	1.0	28.0	99.0
Breast tenderness	0.29	0.24	68.8	13.6	31.2	86.4
Breast swelling/ Bloating/Weight gain	0.31	0.25	80.6	24.0	19.4	76.0
Joint or muscle pain	0.19	0.46	41.5	0.50	58.5	99.5
Headache	0.15	0.33	37.2	2.0	62.8	98.0

Supplementary Table 10. Total explained variance and proportions of fixed and random effects of cyclic time centered around ovulation (GAMM). The total explained variance (R^2 adj) of the GAMM models for each PMDD symptom estimated with PACTS scaled cyclic time centered around ovulation. The total explained variance is further partitioned into proportions of fixed and random effects, presented as percentages.

	**R^2 (adj)**		**Fixed effect (%)**		**Random effect (%)**	
**Symptom**	**PMDD**	**Controls**	**PMDD**	**Controls**	**PMDD**	**Controls**
Depression	0.29	0.35	65.6	0.0	34.4	100
Anxiety	0.29	0.32	58.1	0.10	41.9	99.9
Affective lability	0.32	0.18	64.5	4.90	35.5	95.1
Irritability	0.32	0.12	58.2	5.20	41.8	94.8
Anhedonia	0.29	0.19	65.7	0.70	34.3	99.3
Concentration	0.27	0.31	58.8	1.0	41.2	99.0
Energy loss	0.23	0.16	67.8	3.20	32.2	96.8
Appetite	0.25	0.26	61.5	9.0	38.5	90.1
Sleep	0.23	0.21	59.7	0.10	40.3	99.9
Overwhelmed	0.26	0.16	60.0	0.70	40.0	99.3
Breast tenderness	0.30	0.26	57.7	12.0	42.3	88.0
Breast swelling/ Bloating/Weight gain	0.31	0.26	73.6	23.9	26.4	76.1
Joint or muscle pain	0.20	0.46	35.9	0.50	64.1	99.5
Headache	0.15	0.33	33.2	1.60	66.8	98.4

Supplementary Table 11. Model selection criteria for the temporal pattern analysis. BIC (Bayesian Information Criterion) and AIC (Akaike Information Criterion) values for each model with k=1:4 number of clusters, as well as the number of individuals and the percentage in temporal pattern, for the total DRSP score and each individual PMDD symptom. Entropy values, computed based on number of classes, sample size, and posterior probabilities, for models with k=2:4 are included as a measure of class separation. Model selection was primarily based on a compromise between BIC, AIC, both with as low values as possible, and temporal pattern percentage, which needed to be at least > 5%, but preferably higher, for each temporal pattern. The most appropriate model for the majority of symptoms was with k=3 number of clusters. Because of the moderate sample size, k=4 number of clusters was dismissed for all models and k=3 number of clusters was chosen for the total DRSP score and all symptoms, also to increase comparability between models. Entropy for models with k=3 ranged between 0.82-0.94 for individual PMDD symptom models, and was 0.99 for total DRSP score, indicating good class separation for all models.

**Model**	**BIC**	**AIC**	**Temporal pattern size (N (%))**				**Entropy**
**Total DRSP score**							
k=1	19628.73	19600.34	(1) 90 (100%)				
k=2	19015.08	18958.30	(1) 55 (61.1%)	(2) 35 (38.9%)			0.97
k=3	18820.65	18735.48	(1) 28 (31.1%)	(2) 10 (11.1%)	(3) 52 (57.8%)		0.99
k=4	18707.57	18594.01	(1) 26 (28.9%)	(2) 13 (14.4%)	(3) 46 (51.1%)	(4) 5 (5.60%)	0.98
**Depression**							
k=1	7077.938	7055.226	(1) 90 (100%)				
k=2	6864.137	6813.036	(1) 42 (46.6%)	(2) 48 (53.3%)			0.87
k=3	6845.171	6765.681	(1) 38 (42.2%)	(2) 5 (5.60%)	(3) 47 (52.2%)		0.90
k=4	6843.201	6735.321	(1) 5 (5.60%)	(2) 10 (11.1%)	(3) 35 (38.9%)	(4) 40 (44.4%)	0.88
**Anxiety**							
k=1	7375.413	7352.702	(1) 90 (100%)				
k=2	7114.732	7063.631	(1) 40 (44.4%)	(2) 50 (55.6%)			0.91
k=3	7085.553	7006.063	(1) 22 (24.4%)	(2) 26 (28.9%)	(3) 42 (46.7%)		0.85
k=4	7083.642	6975.763	(1) 6 (6.70%)	(2) 39 (43.3%)	(3) 13 (14.4%)	(4) 32 (35.6%)	0.88
**Affective lability**							
k=1	7339.991	7317.279	(1) 90 (100%)				
k=2	7151.116	7100.015	(1) 49 (54.4%)	(2) 41 (45.6%)			0.84
k=3	7113.794	7034.304	(1) 37 (41.1%)	(2) 5 (5.60%)	(3) 48 (53.3%)		0.90
k=4	7114.582	7006.703	(1) 21 (23.3%)	(2) 29 (32.2%)	(3) 35 (38.9%)	(4) 5 (5.6%)	0.84

**Irritability**							
k=1	7305.113	7282.402	(1) 90 (100%)				
k=2	7144.024	7092.923	(1) 50 (55.6%)		(2) 40 (44.4%)		0.86
k=3	7110.961	7031.471	(1) 46 (51.1%)	(2) 7 (7.80%)	(3) 37 (41.1%)		0.89
k=4	7123.457	7015.578	(1) 38 (42.2%)	(2) 30 (33.3%)	(3) 7 (7.8%)	(4) 15 (16.7%)	0.83
**Anhedonia**							
k=1	7415.523	7392.812	(1) 90 (100%)				
k=2	7264.303	7213.202	(1) 42 (46.7%)		(2) 48 (53.3%)		0.81
k=3	7234.256	7154.766	(1) 34 (37.8%)	(2) 14 (15.6%)	(3) 42 (46.7%)		0.83
k=4	7234.712	7126.832	(1) 27 (30.0%)	(2) 8 (8.9%)	(3) 44 (48.9%)	(4) 11 (12.2%)	0.85
**Concentration**							
k=1	7279.932	7257.220	(1) 90 (100%)				
k=2	7039.625	6988.524	(1) 36 (40.0%)		(2) 54 (60.0%)		0.82
k=3	7003.503	6924.013	(1) 26 (28.9%)	(2) 45 (50.0%)	(3) 19 (21.1%)		0.84
k=4	6985.282	6877.402	(1) 21 (23.3%)	(2) 42 (46.7%)	(3) 20 (22.2%)	(4) 7 (7.80%)	0.85
**Energy loss**							
k=1	7795.049	7772.337	(1) 90 (100%)				
k=2	7635.610	7584.510	(1) 61 (67.8%)		(2) 29 (32.2%)		0.84
k=3	7591.997	7512.507	(1) 24 (26.7%)	(2) 27 (30.0%)	(3) 39 (43.3%)		0.82
k=4	7577.672	7469.792	(1) 33 (36.7%)	(2) 13 (14.4%)	(3) 35 (38.9%)	(4) 9 (10.0%)	0.84
**Appetite**							
k=1	7002.417	6979.705	(1) 90 (100%)				
k=2	6625.804	6574.703	(1) 49 (54.4%)		(2) 41 (45.6%)		0.90
k=3	6605.418	6525.928	(1) 9 (10.0%)	(2) 38 (42.2%)	(3) 43 (47.8%)		0.89
k=4	6619.372	6511.492	(1) 9 (10.0%)	(2) 27 (30.0%)	(3) 44 (48.9%)	(4) 10 (11.1%)	0.86
**Sleep**							
k=1	7098.139	7075.427	(1) 90 (100%)				
k=2	6825.129	6774.028	(1) 65 (72.2%)		(2) 25 (27.8%)		0.93
k=3	6816.674	6737.184	(1) 13 (14.4%)	(2) 13 (14.4%)	(3) 64 (71.1%)		0.91
k=4	6826.184	6718.304	(1) 12 (13.3%)	(2) 11 (12.2%)	(3) 15 (16.7%)	(4) 52 (57.8%)	0.83
**Overwhelmed**							
k=1	6966.254	6943.543	(1) 90 (100%)				
k=2	6718.375	6667.274	(1) 43 (47.8%)		(2) 47 (52.2%)		0.92

k=3	6700.836	6621.346	(1) 37 (41.1%)	(2) 8 (8.9%)	(3) 45 (50.0%)		0.93
k=4	6711.579	6603.700	(1) 2 (2.2%)	(2) 44 (48.9%)	(3) 36 (40.0%)	(4) 8 (8.9%)	0.94
**Breast tenderness**							
k=1	6669.530	6646.818	(1) 90 (100%)				
k=2	6237.236	6186.135	(1) 48 (53.3%)		(2) 42 (46.7%)		0.91
k=3	6141.029	6061.539	(1) 14 (15.6%)	(2) 41 (45.6%)	(3) 35 (38.9%)		0.94
k=4	6144.997	6037.118	(1) 35 (38.9%)	(2) 40 (44.4%)	(3) 10 (11.1%)	(4) 5 (5.60%)	0.94
**Breast swelling/ bloating/weight gain**							
k=1	7304.571	7281.859	(1) 90 (100%)				
k=2	6974.560	6923.459	(1) 38 (42.2%)		(2) 52 (57.8%)		0.90
k=3	6842.225	6762.735	(1) 21 (23.3%)	(2) 44 (48.9%)	(3) 25 (27.8%)		0.88
k=4	6815.312	6707.432	(1) 21 (23.3%)	(2) 23 (25.6%)	(3) 14 (15.6%)	(4) 32 (35.6%)	0.87
**Joint or muscle pain**							
k=1	7010.532	6987.820	(1) 90 (100%)				
k=2	6490.907	6439.807	(1) 33 (36.7%)		(2) 57 (63.3%)		0.93
k=3	6429.331	6349.841	(1) 15 (16.7%)	(2) 32 (35.6%)	(3) 43 (47.8%)		0.91
k=4	6429.845	6321.965	(1) 47 (52.2%)	(2) 15 (16.7%)	(3) 21 (23.3%)	(4) 7 (7.80%)	0.90
**Headache**							
k=1	6726.759	6704.048	(1) 90 (100%)				
k=2	6372.868	6321.767	(1) 71 (78.9%)		(2) 19 (21.1%)		0.95
k=3	6359.127	6279.637	(1) 15 (16.7%)	(2) 22 (24.4%)	(3) 53 (58.9%)		0.86
k=4	6372.181	6264.302	(1) 7 (7.80%)	(2) 8 (8.90%)	(3) 22 (24.4%)	(4) 53 (58.9%)	0.87

Supplementary Table 12. Agreement of participants in the same LCGA class between the original model and the age-adjusted model. The table includes how many participants that belonged to the same class in the original LCGA models for each symptom and total DRSP score as after adjusting for age vs. belonged to a new class after adjustment.

**Symptom**	**N same class**	**N changed class**	**% Agreement between models**
Total DRSP score	88	1	98.8%
Depression	88	1	98.8%
Anxiety	87	2	97.8%
Affective lability	88	1	98.8%
Irritability	83	6	93.3%
Anhedonia	86	3	96.6%
Concentration	51	38	58.4%
Energy loss	77	12	86.5%
Appetite	81	8	91.0%
Sleep	84	5	94.3%
Overwhelmed	87	2	97.8%
Breast tenderness	89	0	100%
Breast swelling/Bloat/Weight gain	86	3	96.6%
Joint or muscle pain	85	4	95.5%
Headache	86	3	96.6%

Supplementary Table 13. Autoregressive and cross-symptom effects of the core PMDD symptoms. The table include autoregressive and cross-symptom effects estimated with CT-VAR on the core PMDD symptoms, together with their 95 % confidence intervals, and Benjamini-Hochberg adjusted p-values.

Symptom 1 → Symptom 2	Autoregressive / Cross-symptom effect	P adjusted	95% CI [lower, higher]
Depression → Depression	-0.65	< 0.001	[-0.85, -0.49]
Depression → Anxiety	-0.18	0.069	[-0.36, 0.003]
Depression → Affective lability	-0.30	0.001	[-0.46, -0.13]
Depression → Irritability	-0.34	< 0.001	[-0.49, -0.18]
Anxiety → Depression	0.26	< 0.001	[0.12, 0.38]
Anxiety → Anxiety	-0.30	0.011	[-0.57, -0.14]
Anxiety → Affective lability	0.24	< 0.001	[0.12, 0.38]
Anxiety → Irritability	0.26	< 0.001	[0.13, 0.40]
Affective lability → Depression	0.14	0.134	[-0.03, 0.32]
Affective lability → Anxiety	0.11	0.178	[-0.05, 0.27]
Affective lability → Affective lability	-0.25	0.036	[-0.53, -0.10]
Affective lability → Irritability	0.17	0.128	[-0.04, 0.37]
Irritability → Depression	0.08	0.178	[-0.04, 0.19]
Irritability → Anxiety	0.20	0.005	[0.07, 0.33]
Irritability → Affective lability	0.13	0.069	[-0.0004, 0.26]
Irritability → Irritability	-0.28	0.005	[-0.50, -0.14]

Supplementary Table 14. Effect of age on drift parameters estimated in the age-adjusted CT-VAR model.

**Symptom 1 → Symptom 2**	**Age moderation estimate**	**95% CI [lower, higher]**	**Interpretation**
Anxiety → Depression	0.12	[0.02, 0.23]	The significant positive effect of anxiety on the rate of change of depression is larger for older participants
Affective lability → Affective lability	0.10	[0.04, 0.18]	The rate of which affective lability returns to equilibrium is slower for older participants
Irritability → Depression	0.16	[0.03, 0.29]	The effect of irritability on the rate of change of depression is larger for older participants
Irritability → Affective lability	-0.17	[-0.31, -0.06]	The effect of irritability on the rate of change of affective lability is smaller for older participants
Irritability → Irritability	0.13	[0.07, 0.22]	The rate of which irritability returns to equilibrium is slower for older participants

Supplementary Table 15. Cross-symptom lagged effects. Maximum values of cross-lagged effects at the corresponding Δt (time between measurement) for each pair of symptoms, derived from the estimated lagged effects based on the CT-VAR model.

**Cross-symptom effect**	**Maximum effect size**	**Δt (days)**
Depression → Anxiety	-0.31	4.4
Depression → Affective lability	-0.38	3.7
Depression → Irritability	-0.41	3.4
Anxiety → Depression	0.27	3.1
Anxiety → Affective lability	0.27	3.1
Anxiety → Irritability	0.27	2.9
Affective lability → Depression	N.S.	-
Affective lability → Anxiety	N.S.	-
Affective lability → Irritability	N.S.	-
Irritability → Depression	0.20	5.0
Irritability → Anxiety	0.27	3.8
Irritability → Affective lability	0.24	4.4

Supplementary Table 16. DSM-5 domains for the PMDD diagnosis. The core and secondary symptoms of PMDD, in relation with the DSM-5 domains, the domain labels used in this analysis, and the corresponding items in the DRSP questionnaire.

	DSM-5 domain	Label	DRSP item
Core symptoms	Markedly depressed mood	Depression	Felt depressed, sad, “down” or blue
			Felt hopeless
			Felt worthless or guilty
	Marked anxiety and tension	Anxiety	Felt anxious, “keyed up” or “on edge”
	Marked affective lability	Affective lability	Had mood swings
			Was more sensitive to rejection or easily hurt
	Marked irritability or anger	Irritability	Felt angry, irritable
			Had conflicts or problems with people
Secondary symptoms	Decreased interest in usual activities	Anhedonia	Had less interest in usual activities
	Difficulty in concentration	Concentration	Had difficulty concentrating
	Lethargy and marked lack of energy	Energy loss	Felt lethargic, tired, fatigued, or had a lack of energy
	Marked change in appetite	Appetite	Had increased appetite or overate
			Had specific food craving
	Hypersomnia or insomnia	Sleep	Slept more, took naps, found it hard to get up
			Had trouble getting to sleep or staying asleep
	Feeling overwhelmed or out of control	Overwhelmed	Felt overwhelmed, that I couldn’t cope
			Felt out of control
	Physical symptoms	Breast tenderness	Had breast tenderness
		Breast swelling/ bloating/weight gain	Had breast swelling, felt bloated, or had weight gain
		Joint or muscle pain	Had joint or muscle pain
	-	Headache	Had headache

SUPPLEMENTARY FIGURES

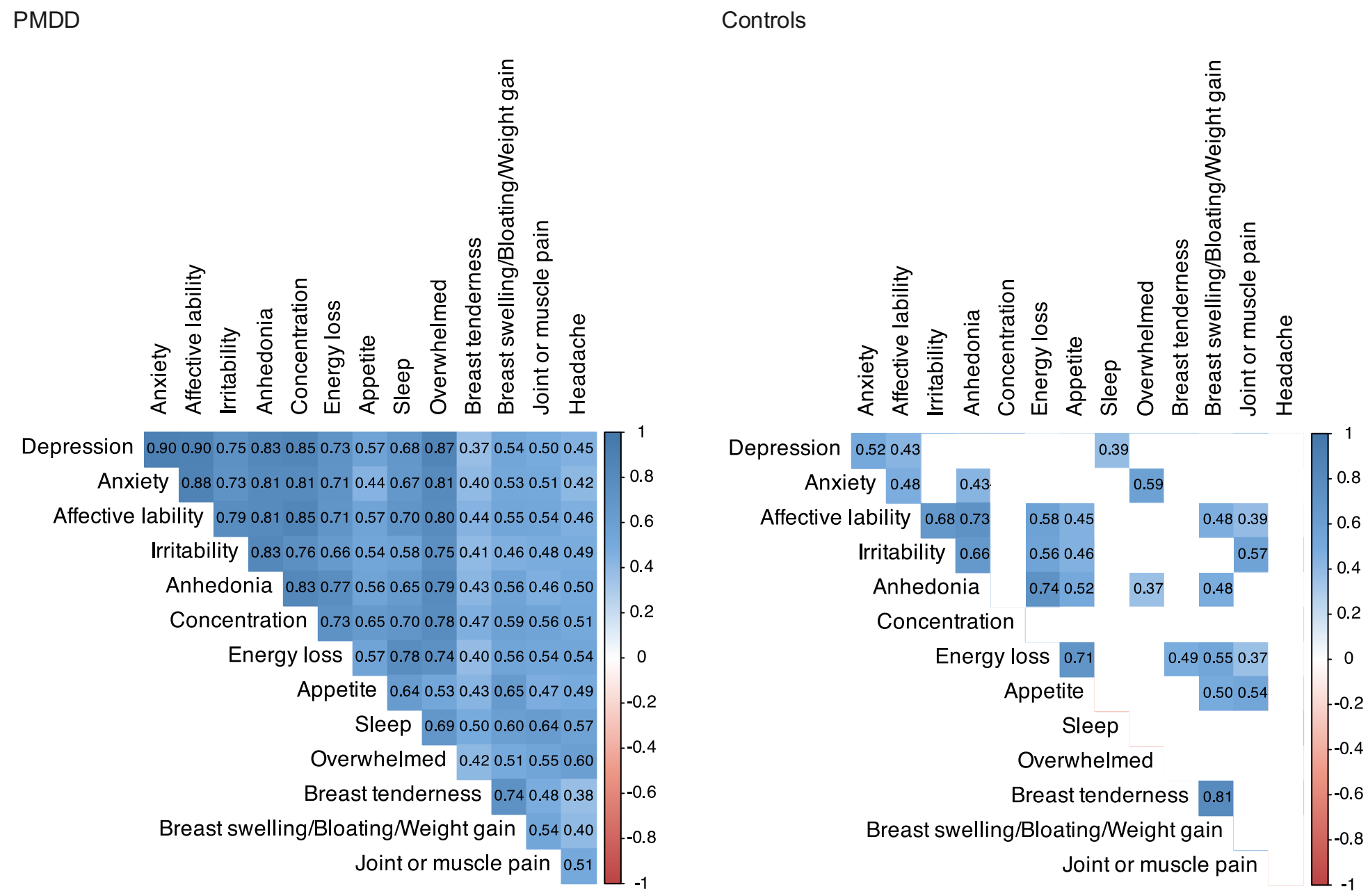

Supplementary Figure 1. Relationships between variation of PMDD symptoms over the menstrual cycle. The correlation matrices show the significant correlations between each PMDD symptom separately for patients with PMDD (n=90) and controls (n=44), based on the cosine amplitudes estimated from the multilevel cosine regression models estimated with the PACTS scaled time variable. Benjamini-Hochberg correction was applied to adjust for multiple comparisons. See Supplementary Table 3 for adjusted p-values and 95% CI.

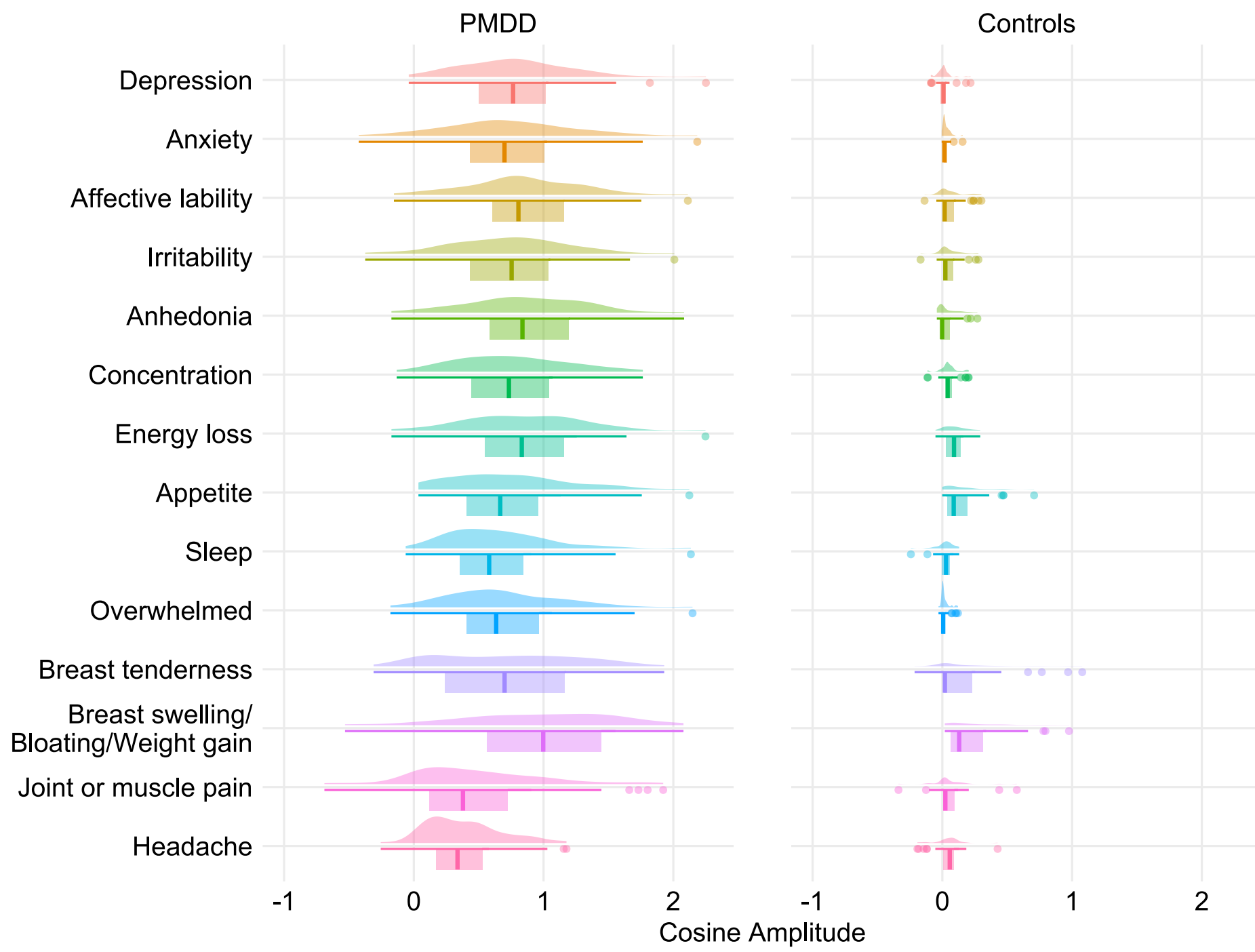

Supplementary Figure 2. Variability and severity of premenstrual symptoms across the menstrual cycle in patients with PMDD and controls (proportional time variable scaled uniformly). Visualized are the estimated values of cosine amplitudes (x-axis) for patients with PMDD (n=90) and controls (n=44) yielded from the final multilevel cosine regression models for each individual symptom (y-axis). The cosine amplitudes correspond both to the timing and severity of symptoms across the menstrual cycle. Timing is indicated by the sign of the value, where positive values indicate a premenstrual increase of symptoms, negative values correspond to a mid-cycle increase of symptoms, and values of zero indicate no variation in symptomatology across the menstrual cycle. Severity is indicated by the magnitude of the cosine amplitude value, where larger/more negative values correspond to a higher severity of symptom premenstrually/mid-cycle, respectively. Each box represents 50% of the data, with the median line in bold. The whiskers extend to the minimum and maximum of the data, except for those with outliers which are represented by the dots. The violin plot illustrates the density distribution of the cosine amplitudes (i.e., a flatter curve indicates that the values are more spread along the values on the x-axis). The cosine regression models that the cosine amplitudes stem from are estimated using a proportional time variable across two menstrual cycles, that is scaled uniformly based on the length of each cycle.

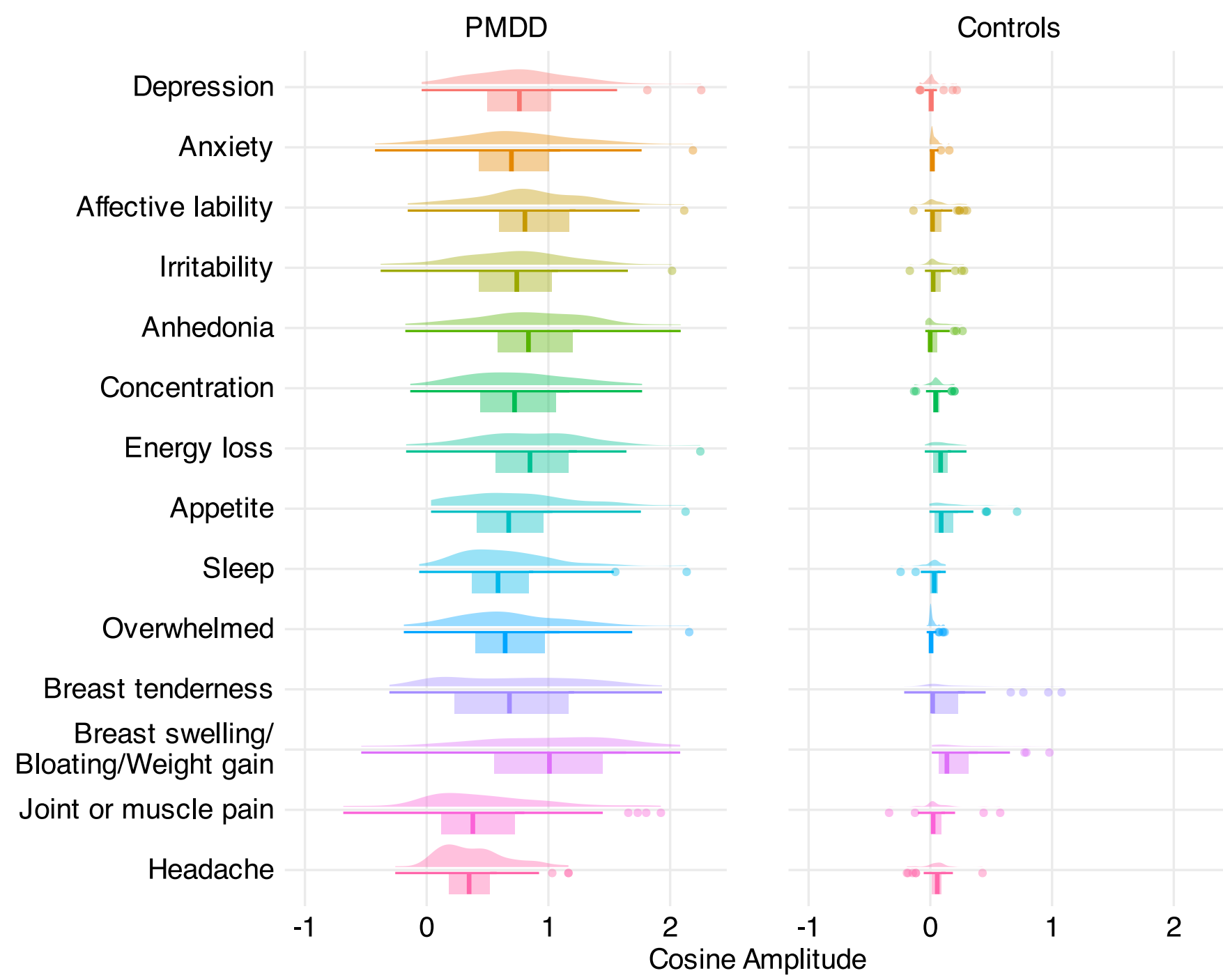

Supplementary Figure 3. Distribution of cosine amplitudes adjusting for age. The figure shows the distribution of the individual cosine amplitudes from the separate cosine regression models for each PMDD symptom for patients with PMDD (n=90) and controls (n=44), when adjusting for age in the models. Timing is indicated by the sign of the value, where positive values indicate a premenstrual increase of symptoms, negative values correspond to a mid-cycle increase of symptoms, and values of zero indicate no variation in symptomatology across the menstrual cycle. Severity is indicated by the magnitude of the cosine amplitude value, where larger/more negative values correspond to a higher severity of symptom premenstrually/mid-cycle, respectively. Each box represents 50% of the data, with the median line in bold. The whiskers extend to the minimum and maximum of the data, except for those with outliers which are represented by the dots. The violin plot illustrates the density distribution of the cosine amplitudes (i.e., a flatter curve indicates that the values are more spread along the values on the x-axis).

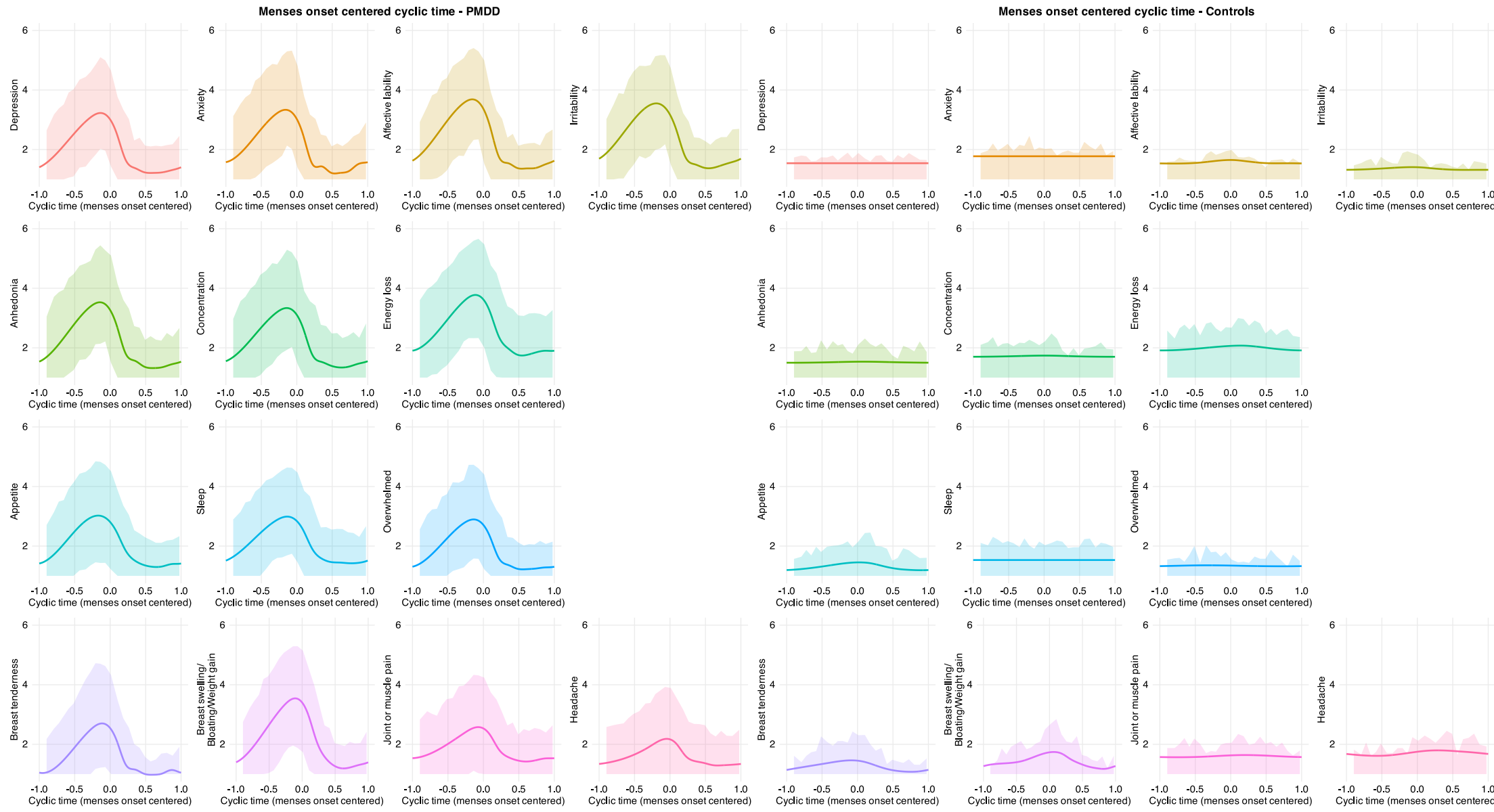

Supplementary Figure 4. GAMM estimated with PACTS scaled cyclic time centered on menses onset (Left: PMDD; Right: Controls). The figure shows the average symptom trajectories for each symptom estimated with GAMM based on n=90 patients with PMDD and n=44 controls, with PACTS scaled cyclic time centered around menses onset. The shaded area around the average is the ±1SD of raw DRSP scores.

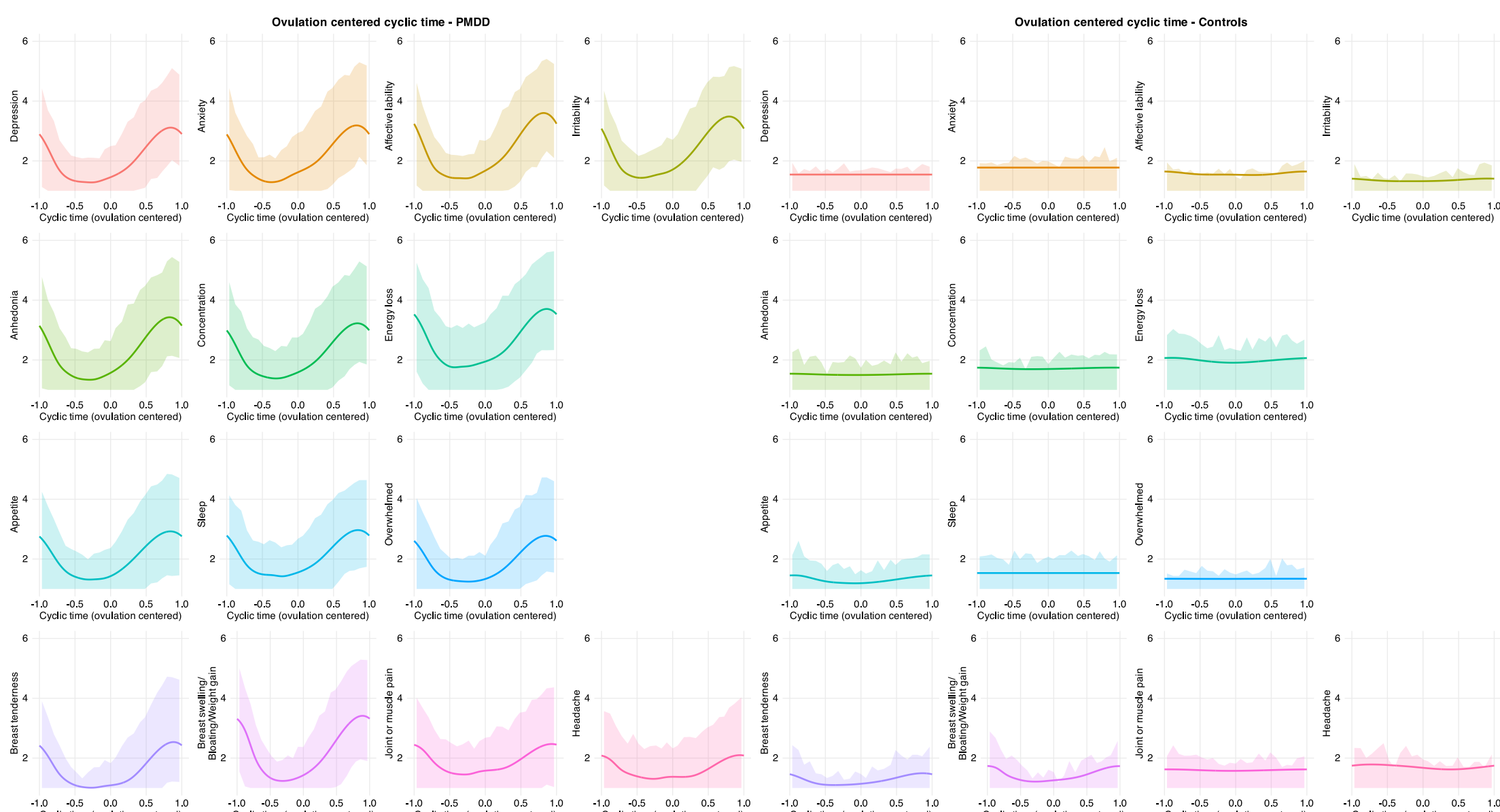

Supplementary Figure 5. GAMM estimated with PACTS scaled cyclic time centered on ovulation (Left: PMDD; Right: Controls). The figure shows the average symptom trajectories for each symptom estimated with GAMM based on n=90 patients with PMDD and n=44 controls, with PACTS scaled cyclic time centered around ovulation. The shaded area around the average is the ±1SD of raw DRSP scores.

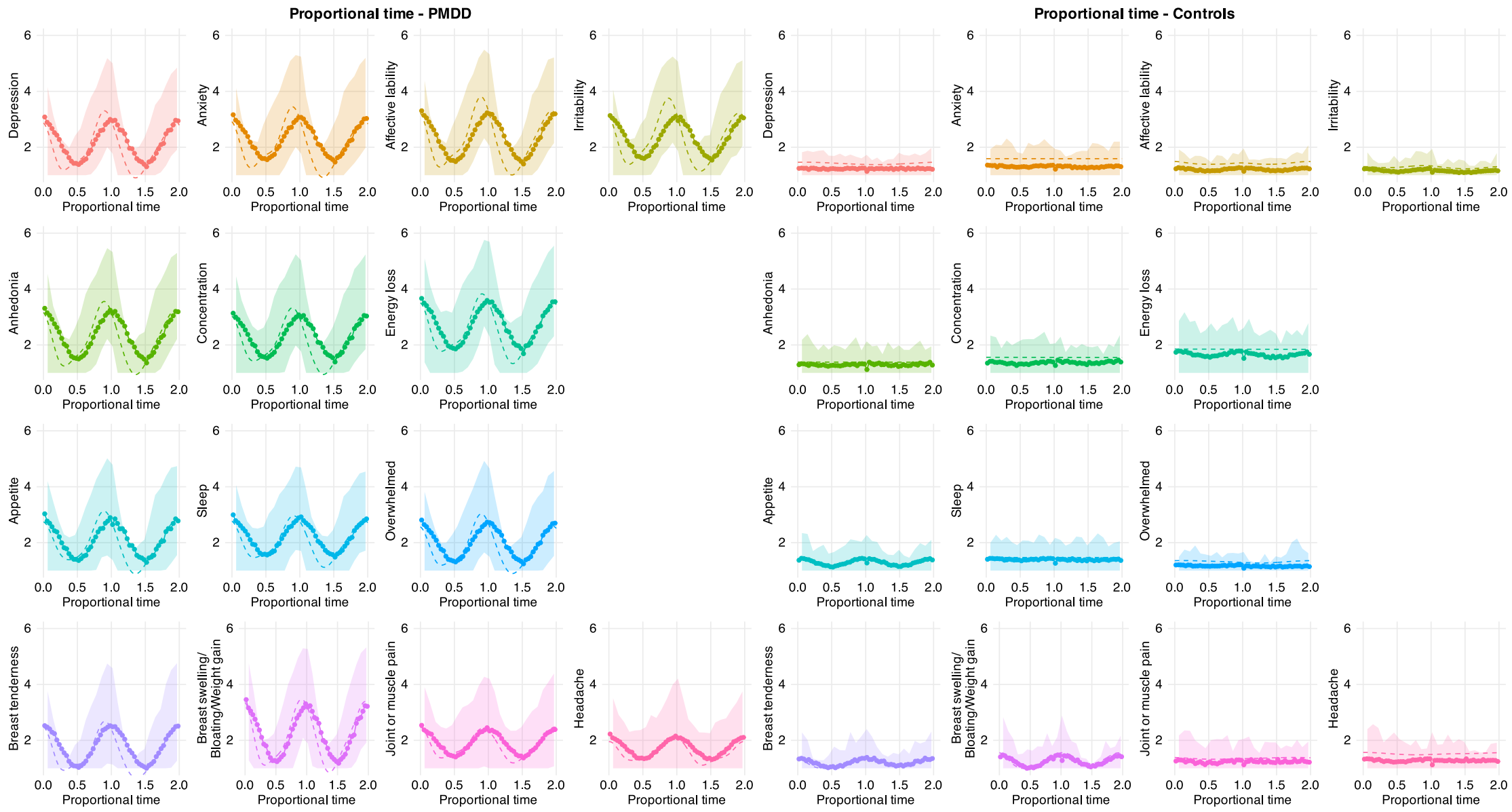

Supplementary Figure 6. Superimposed results of the two statistical approaches to facilitate comparison and illustrate their degree of overlap for the average GAMM and Cosine regression curves (Left: PMDD; Right: controls). The figure shows the average symptom trajectories for each symptom estimated with GAMM (dashed lines) and cosine regression (solid line with dots), both based on n=90 patients with PMDD and n=44 controls, with a proportional time variable anchored to menses onset. The shaded area around the average is the ±1SD of raw DRSP scores.

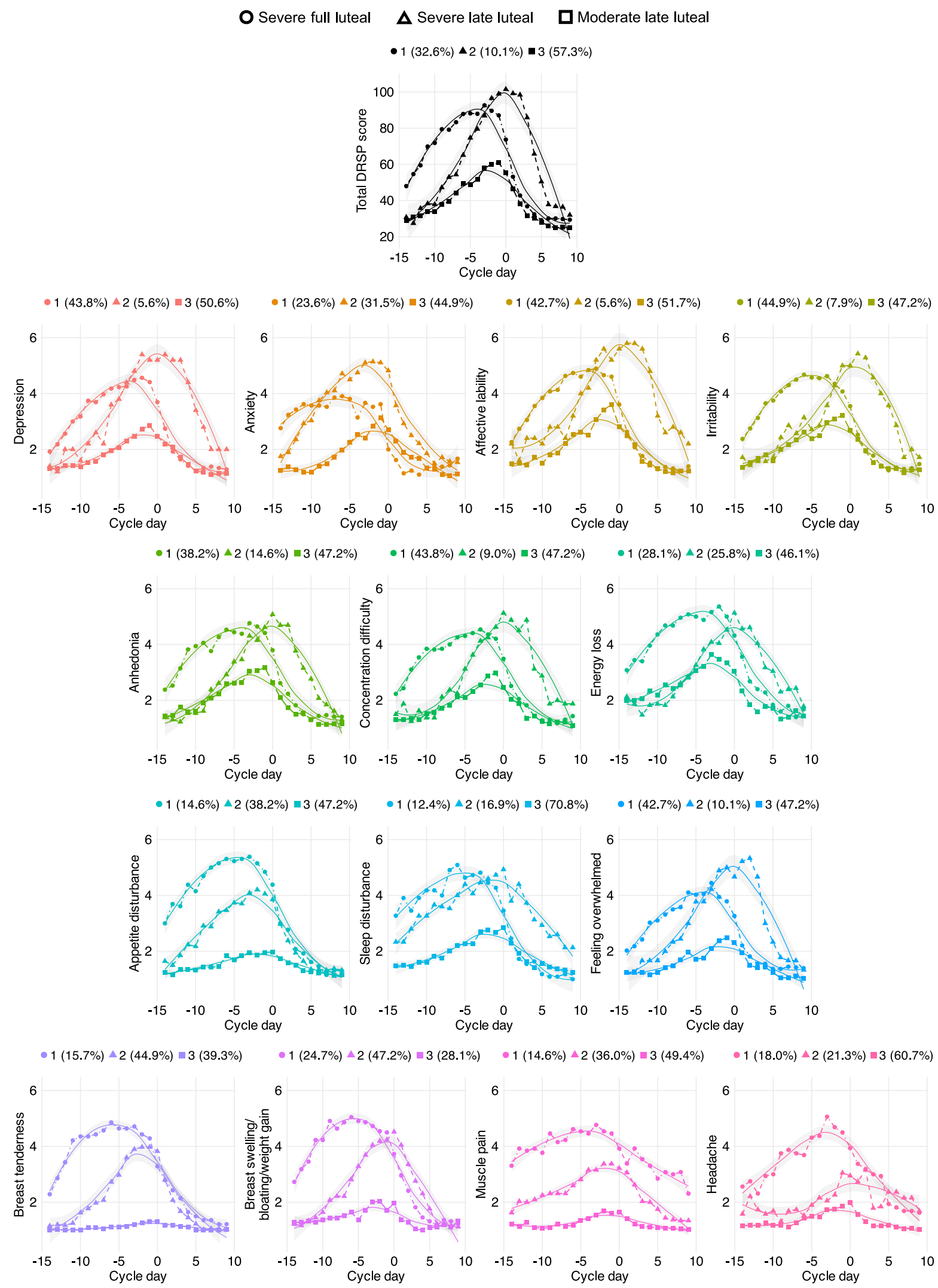

Supplementary Figure 7. Temporal patterns of premenstrual symptoms (age adjusted). The graphs show the result of the LCGA models adjusted for age with the k=3 solution for the total DRSP score and each individual symptom. All models are based on n=90 patients with PMDD. Lines with circles/triangles/squares show the average score for menstrual cycle day -14 to +9 of participants included in each pattern, while solid lines show a smoothed curve with a shaded 95% confidence interval.

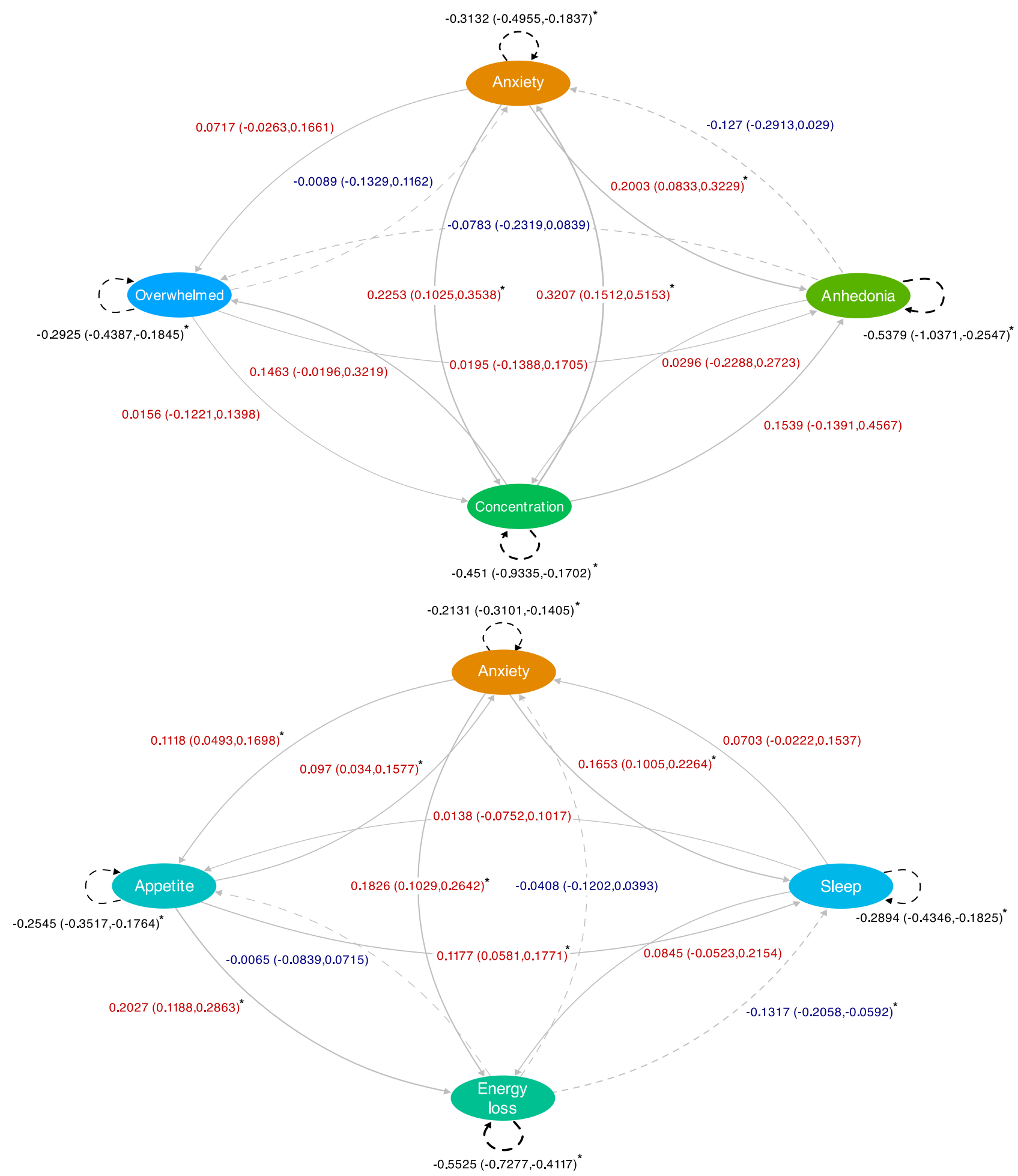

Supplementary Figure 8. Dynamic networks of additional CT-VAR models. The top network shows the cross-symptom and autoregressive effects of the CT-VAR model including anxiety together with the secondary psychological symptoms of PMDD; anhedonia, concentration, and feeling overwhelmed. The bottom network shows the cross-symptom and autoregressive effects of the CT-VAR model including anxiety together with the vegetative symptoms of PMDD; energy loss, appetite, and sleep. Both CT-VAR models are based on n=90 patients with PMDD. * Indicate effects that were significant after correction for multiple testing using the Benjamini-Hochberg correction. See Figure 6 for a full description of model parameters and interpretation. Exact adjusted p-values for each effect (top network): Anxiety → Anxiety ($p < 0.001$); Anxiety → Anhedonia ($p = 0.003$); Anxiety → Concentration ($p = 0.002$); Anxiety → Overwhelmed ($p = 0.231$); Anhedonia → Anxiety ($p = 0.214$); Anhedonia → Anhedonia ($p = 0.019$); Anhedonia → Concentration ($p = 0.871$); Anhedonia → Overwhelmed (0.441); Concentration → Anxiety ($p = 0.002$); Concentration → Anhedonia ($p = 0.441$); Concentration → Concentration ($p = 0.047$); Concentration → Overwhelmed ($p = 0.186$). Exact p-values for each effect (bottom network): Anxiety → Anxiety ($p < 0.001$); Anxiety → Sleep ($p < 0.001$); Anxiety → Energy loss ($p < 0.001$); Anxiety → Appetite ($p < 0.001$); Sleep → Anxiety ($p = 0.156$); Sleep → Sleep ($p < 0.001$); Sleep → Energy loss ($p = 0.266$); Sleep → Appetite ($p = 0.810$); Energy loss → Anxiety ($p = 0.361$); Energy loss → Sleep ($p < 0.001$); Energy loss → Energy loss ($p < 0.001$); Energy loss → Appetite ($p = 0.870$); Appetite → Anxiety ($p = 0.003$); Appetite → Sleep ($p < 0.001$); Appetite → Energy loss ($p < 0.001$); Appetite → Appetite ($p < 0.001$).

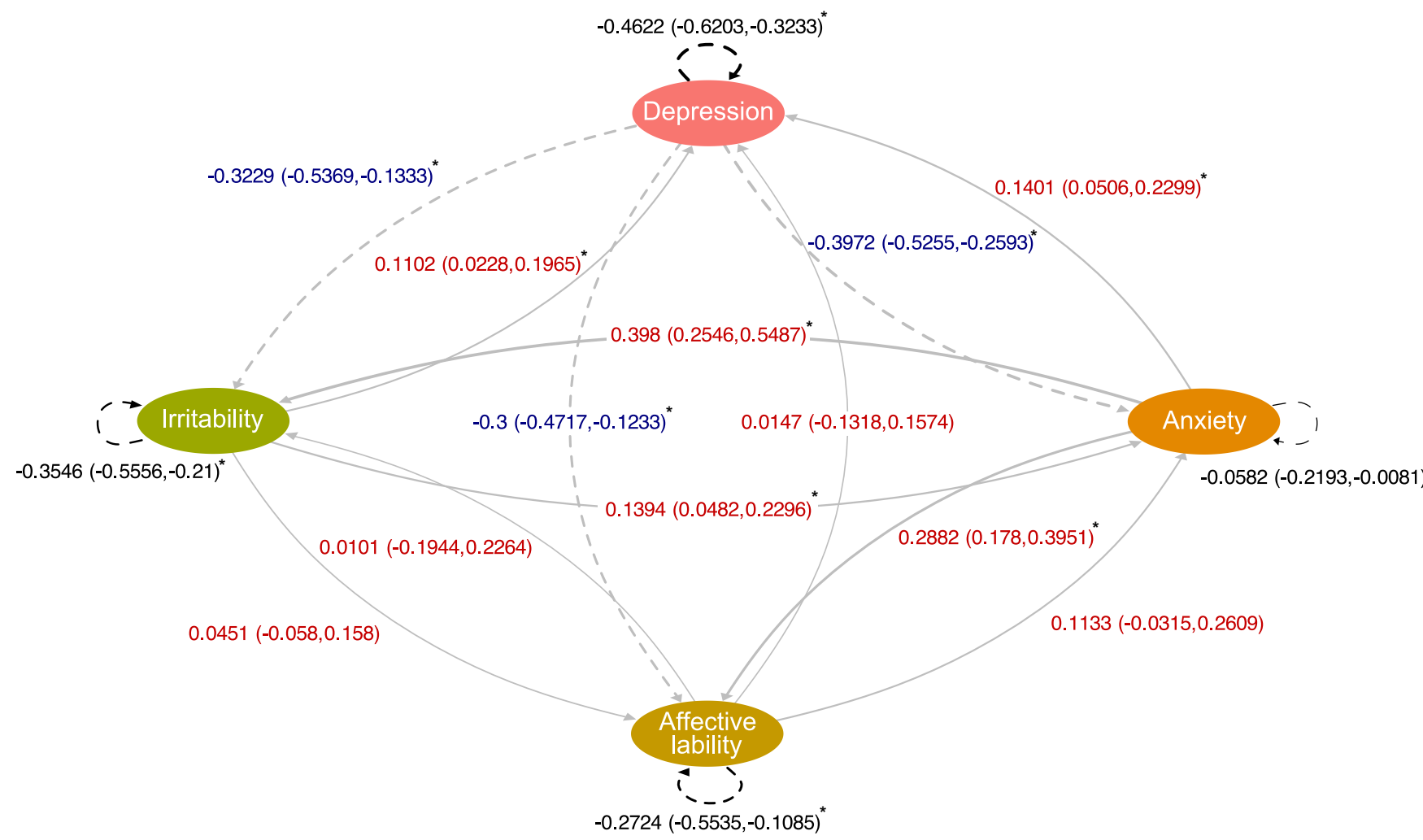

Supplementary Figure 9. Dynamic network of the core symptoms estimated with the less symptomatic cycle. Solid lines represent positive effects, and dashed lines represent negative effects. The network is derived from one CT-VAR model including all four core symptoms of PMDD, that estimated temporal dynamics considering all days from the less severe cycle for each patient with PMDD. The CT-VAR model is based on n=90 patients with PMDD. * Indicate effects that were significant after correction for multiple testing using the Benjamini-Hochberg correction. See Figure 6 for a full description of model parameters and interpretation. Exact adjusted p-values for each effect: Depression → Depression ($p < 0.001$); Depression → Anxiety ($p < 0.001$); Depression → Affective lability ($p = 0.002$); Depression → Irritability ($p = 0.004$); Anxiety → Depression ($p = 0.004$); Anxiety → Anxiety ($p = 0.345$); Anxiety → Affective lability ($p < 0.001$); Anxiety → Irritability ($p < 0.001$); Affective lability → Depression ($p = 0.898$); Affective lability → Anxiety ($p = 0.172$); Affective lability → Affective lability ($p = 0.024$); Affective lability → Irritability ($p = 0.925$); Irritability → Depression ($p = 0.021$); Irritability → Anxiety ($p = 0.005$); Irritability → Affective lability ($p = 0.472$); Irritability → Irritability ($p < 0.001$).

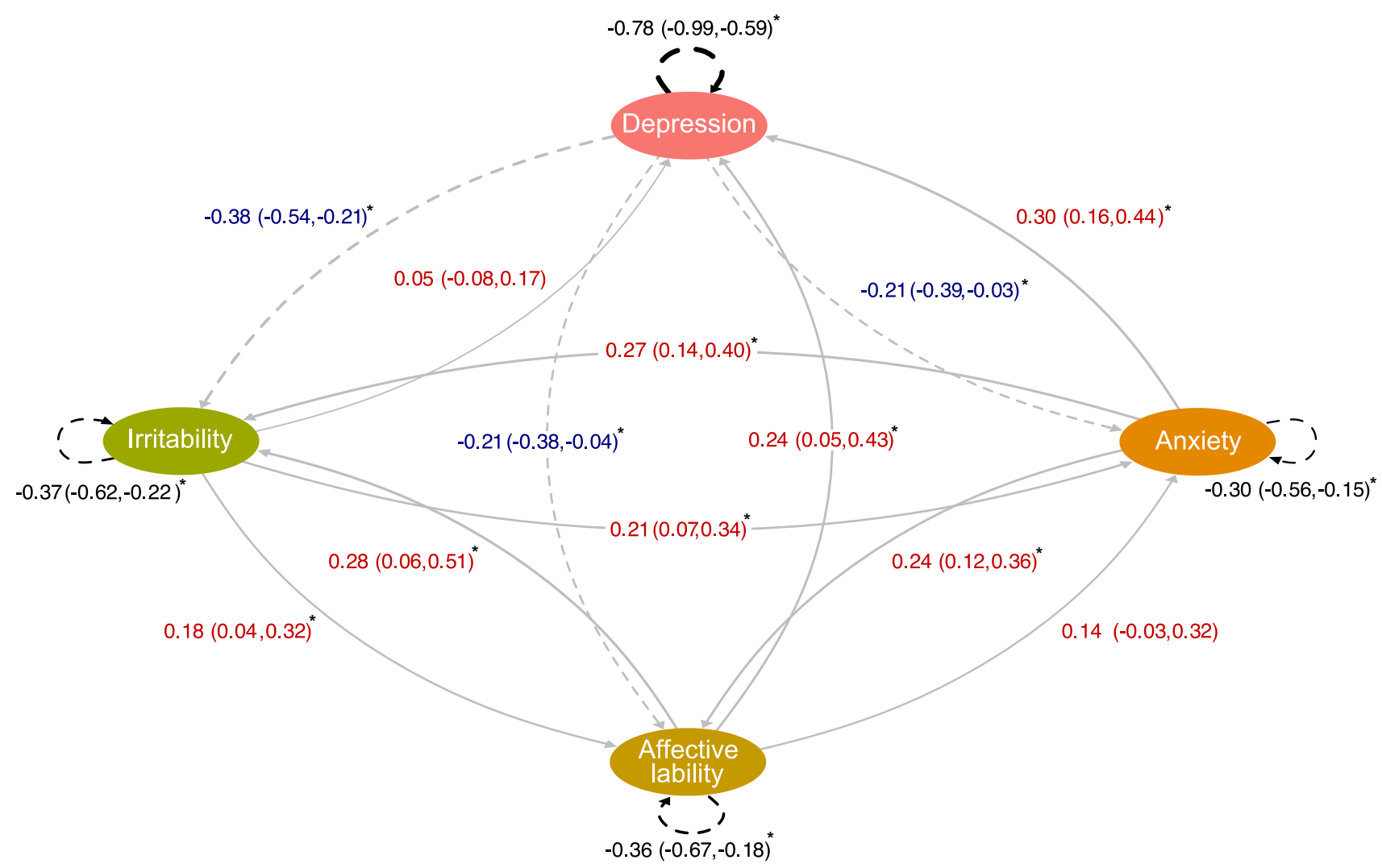

Supplementary Figure 10. Dynamic network of the core symptoms (age-adjusted). Solid lines represent positive effects, and dashed lines represent negative effects. The network is derived from one CT-VAR model including all four core symptoms of PMDD while adjusting for age. The CT-VAR model is based on n=90 patients with PMDD. * Indicate effects that were significant after correction for multiple testing using the Benjamini-Hochberg correction. See Figure 6 for a full description of model parameters and interpretation. Exact adjusted p-values for each effect: Depression → Depression ($p < 0.001$); Depression → Anxiety ($p = 0.018$); Depression → Affective lability ($p = 0.015$); Depression → Irritability ($p < 0.001$); Anxiety → Depression ($p < 0.001$); Anxiety → Anxiety ($p = 0.006$); Anxiety → Affective lability ($p < 0.001$); Anxiety → Irritability ($p < 0.001$); Affective lability → Depression ($p = 0.015$); Affective lability → Anxiety ($p = 0.120$); Affective lability → Affective lability ($p = 0.004$); Affective lability → Irritability ($p = 0.015$); Irritability → Depression ($p = 0.437$); Irritability → Anxiety ($p = 0.002$); Irritability → Affective lability ($p = 0.013$); Irritability → Irritability ($p < 0.001$).

SUPPLEMENTARY REFERENCES

1. Nagpal, A., *et al.* How to study the menstrual cycle as a continuous variable: Implementing phase-aligned cycle time scaling (PACTS) with the menstrualcycleR package. *Psychoneuroendocrinology* **181**, 107584 https://doi.org/10.1016/j.psyneuen.2025.107584 (2025).

2. Kiesner, J., Eisenlohr-Moul, T. A. & Vidotto, G. Affective Risk Associated With Menstrual Cycle Symptom Change. *Front Glob Womens Health* **3**, 896924 https://doi.org/10.3389/fgwh.2022.896924 (2022).
