# Supplementary material for: Temporal dynamics of premenstrual dysphoric disorder revealed through predictive symptom networks
Source: Nat Commun. 2026 Nov 10;17:11165. doi: 10.1038/s41467-026-78590-x (PMC13751610; doi:10.1038/s41467-026-78590-x)
Supplement: Supplementary file 2 [file 41467_2026_78590_MOESM2_ESM.pdf]

nature portfolio

Corresponding author(s): Erika Comasco

Last updated by author(s): Sep 22, 2026

Reporting Summary

Nature Portfolio wishes to improve the reproducibility of the work that we publish. This form provides structure for consistency and transparency in reporting. For further information on Nature Portfolio policies, see our Editorial Policies and the Editorial Policy Checklist.

Statistics

For all statistical analyses, confirm that the following items are present in the figure legend, table legend, main text, or Methods section.

n/a	Confirmed	
☐	☒	The exact sample size (n) for each experimental group/condition, given as a discrete number and unit of measurement
☐	☒	A statement on whether measurements were taken from distinct samples or whether the same sample was measured repeatedly
☐	☒	The statistical test(s) used AND whether they are one- or two-sided<br>*Only common tests should be described solely by name; describe more complex techniques in the Methods section.*
☐	☒	A description of all covariates tested
☐	☒	A description of any assumptions or corrections, such as tests of normality and adjustment for multiple comparisons
☐	☒	A full description of the statistical parameters including central tendency (e.g. means) or other basic estimates (e.g. regression coefficient) AND variation (e.g. standard deviation) or associated estimates of uncertainty (e.g. confidence intervals)
☐	☒	For null hypothesis testing, the test statistic (e.g. F, t, r) with confidence intervals, effect sizes, degrees of freedom and P value noted<br>*Give P values as exact values whenever suitable.*
☒	☐	For Bayesian analysis, information on the choice of priors and Markov chain Monte Carlo settings
☐	☒	For hierarchical and complex designs, identification of the appropriate level for tests and full reporting of outcomes
☐	☒	Estimates of effect sizes (e.g. Cohen's d, Pearson's r), indicating how they were calculated

Our web collection on statistics for biologists contains articles on many of the points above.

Software and code

Policy information about availability of computer code

Data collection	All analyses were performed in RStudio with R version 4.3.0, as described in the Methods.
Data analysis	Custom code is available at https://github.com/EllaS-J

For manuscripts utilizing custom algorithms or software that are central to the research but not yet described in published literature, software must be made available to editors and reviewers. We strongly encourage code deposition in a community repository (e.g. GitHub). See the Nature Portfolio guidelines for submitting code & software for further information.

Data

Policy information about availability of data

All manuscripts must include a data availability statement. This statement should provide the following information, where applicable:

- Accession codes, unique identifiers, or web links for publicly available datasets
- A description of any restrictions on data availability
- For clinical datasets or third party data, please ensure that the statement adheres to our policy

All data used to reach the conclusions presented in this study are presented in the main text or in the supplement. The raw data collected and analysed during the current study are only available under restricted access due to participant privacy and ethical restrictions, access can be obtained by contacting the corresponding author, subject to appropriate ethical approval and data-sharing agreements. The generated data underlying the figures in the study are provided in the Source Data file.

Research involving human participants, their data, or biological material

Policy information about studies with human participants or human data. See also policy information about sex, gender (identity/presentation), and sexual orientation and race, ethnicity and racism.

Reporting on sex and gender	Biological sex was operationalized through the study eligibility criteria, which restricted inclusion to participants with regular menstrual cycles, that is with an ovulatory reproductive physiology, as described in details in the Methods; gender was not assessed.
Reporting on race, ethnicity, or other socially relevant groupings	Race, ethnicity and other socially relevant groupings were not collected for this study and were therefore not analyzed.
Population characteristics	This prospective study included 134 individuals with regular menstrual cycles. Participants' characteristics are presented in Supplementary Table 1. Participants were recruited according to predefined inclusion and exclusion criteria, as described in details in the Methods.
Recruitment	Through advertisements in local newspapers, on university campuses, and social media, as described in details in the Methods.
Ethics oversight	The study was approved by the Regional Ethical Review Board in Uppsala (Dnr. 2016/184 and 2016/312), and written informed consent was obtained from all participants, as described in details in the Methods.

Note that full information on the approval of the study protocol must also be provided in the manuscript.

Field-specific reporting

Please select the one below that is the best fit for your research. If you are not sure, read the appropriate sections before making your selection.

☒ Life sciences ☐ Behavioural & social sciences ☐ Ecological, evolutionary & environmental sciences

For a reference copy of the document with all sections, see nature.com/documents/nr-reporting-summary-flat.pdf

Life sciences study design

All studies must disclose on these points even when the disclosure is negative.

Sample size	No statistical method was used to predetermine sample size. The sample size was based on the number of eligible participants available during the study period who met the inclusion criteria. This sample size is larger compared to those used in others studies in the field.
Data exclusions	Participants were excluded if they had missing key data,that is sufficient ratings during the two screening months, as described in details in the Methods.
Replication	The study was based on a single human cohort dataset, and no independent experimental replication was performed. All available eligible participants were included as described in the Methods.
Randomization	No randomization was performed. Participants were grouped according to clinical status / predefined study criteria, as described in details in the Methods.
Blinding	No blinding was performed. Participants were classified according to predefined criteria, and group allocation was known during data collection and analysis.

Reporting for specific materials, systems and methods

We require information from authors about some types of materials, experimental systems and methods used in many studies. Here, indicate whether each material, system or method listed is relevant to your study. If you are not sure if a list item applies to your research, read the appropriate section before selecting a response.

Materials & experimental systems

n/a	Involved in the study	
☒	☐	Antibodies
☒	☐	Eukaryotic cell lines
☒	☐	Palaeontology and archaeology
☒	☐	Animals and other organisms
☐	☒	Clinical data
☒	☐	Dual use research of concern
☒	☐	Plants

Methods

n/a	Involved in the study	
☒	☐	ChIP-seq
☒	☐	Flow cytometry
☒	☐	MRI-based neuroimaging

Clinical data

Policy information about clinical studies

All manuscripts should comply with the ICMJE guidelines for publication of clinical research and a completed CONSORT checklist must be included with all submissions.

Clinical trial registration

This observational, prospective study was performed as part of two double-blind randomized control trials, one performed in Uppsala, Stockholm, Danderyd, and Umeå starting 2016, and one performed in Uppsala between 2016-2024. The trials were registered as EUDRA-CT 2016-001217-25 and EUDRA-CT 2016-001719-19. Data presented in this study only relate to the initial screening/diagnostic phase for these studies.

Study protocol

The study was conducted according to a predefined protocol approved by the ethics committee and registered as EUDRA-CT 2016-001217-25 and EUDRA-CT 2016-001719-19.

Data collection

Clinical data were collected prospectively during the two-month screening phase, including daily records according to predefined study procedures, as described in details in the Methods.

Outcomes

The primary outcome was PMDD symptom severity assessed using the DRSP, as described in details in the Methods.

Plants

Seed stocks

Report on the source of all seed stocks or other plant material used. If applicable, state the seed stock centre and catalogue number. If plant specimens were collected from the field, describe the collection location, date and sampling procedures.

Novel plant genotypes

Describe the methods by which all novel plant genotypes were produced. This includes those generated by transgenic approaches, gene editing, chemical/radiation-based mutagenesis and hybridization. For transgenic lines, describe the transformation method, the number of independent lines analyzed and the generation upon which experiments were performed. For gene-edited lines, describe the editor used, the endogenous sequence targeted for editing, the targeting guide RNA sequence (if applicable) and how the editor was applied.

Authentication

Describe any authentication procedures for each seed stock used or novel genotype generated. Describe any experiments used to assess the effect of a mutation and, where applicable, how potential secondary effects (e.g. second site T-DNA insertions, mosiacism, off-target gene editing) were examined.
