# Supplementary material for: Temporal dynamics of premenstrual dysphoric disorder revealed through predictive symptom networks
Source: Nat Commun. 2026 Nov 10;17:11165. doi: 10.1038/s41467-026-78590-x (PMC13751610; doi:10.1038/s41467-026-78590-x)
Supplement: Supplementary file 3 [file 41467_2026_78590_MOESM3_ESM.pdf]

nature portfolio

Peer Review File

Temporal Dynamics of Premenstrual Dysphoric Disorder Revealed Through Predictive Symptom Networks

Corresponding Author: Professor Erika Comasco

This file contains all reviewer reports in order by version, followed by all author rebuttals in order by version.

Version 0:

Reviewer comments:

Reviewer #1

(Remarks to the Author)
Overall:

Thank you for the opportunity to review this manuscript, which I read with great interest. In this study, the authors apply innovative computational and statistical approaches to examine daily affective symptom dynamics in individuals with and without premenstrual dysphoric disorder (PMDD). The approaches used are in line with our evolving understanding of PMDD as a heterogeneous disorder with important individual differences in symptom timing, severity, and specific symptoms. Overall, this is a rigorous and potentially impactful manuscript; the findings have the potential to enhance diagnostic precision and PMDD treatment individualization.

The manuscript has numerous strengths. The prospective assessment of daily symptoms across multiple menstrual cycles and including both cases and controls strengthens the reliability by providing repeated within-person measurements and an appropriate comparison group. The approach to excluding participants due to the small number of data points is appropriate, reasonable, and transparently reported. Despite these strengths, I have several suggestions for improvement, outlined below.

Participants/ Study Procedure:

More detail about the two participant samples is needed (even if only in the supplement) as this may have implications for generalizability to the PMDD population as a whole. For example, what studies were participants enrolling in? Were they treatment trials? How were the studies advertised? Relatedly, how were control participants recruited? Also, how were psychiatric comorbidities assessed (to inform participant exclusion)?

It is unclear whether gender was assessed in the study. The manuscript uses "women" to refer to individuals experiencing menstrual cycles. Please clarify whether sex and gender were assessed and report this data in the sample characteristics Table if available. The authors may also consider revising the terminology throughout the manuscript to use more precise and inclusive language (e.g., "menstruating individuals").

The diagnostic threshold used for PMDD appears to require symptom increases exceeding 50%, rather than the commonly used threshold of 30% (e.g., Eisenlohr-Moul et al. Am J Psychiatry. 2017 Jan 1;174(1):51-59). The sample may therefore encompass patients with more severe symptom presentations than the general PMDD population, which may have implications for the study results. It is suggested that the authors discuss the implications of using this strict threshold.

The analyses align the menstrual cycle to the onset of menses, but no information is provided regarding the assessment of ovulation. Without biological confirmation of ovulation, there is a risk of misalignment for the periovulatory and luteal phase, particularly in shorter or longer cycles. Please clarify whether ovulation was assessed and, if appropriate, discuss the potential impact of this limitation on the interpretation of symptom timing and cycle phase alignment.

Statistical Approach:

It is not clear whether the research questions or the analytic plan was preregistered. Clarifying this would improve

transparency. If the analyses were not preregistered, it may be more appropriate to frame the findings as exploratory.

The purpose of the cosine analysis could be more clearly explained. Please also justify/explain the use of the cosine analysis as opposed to an approach such as PACTS (Nagpal et al. (2025). Psychoneuroendocrinology, 107584.). The cosine approach appears to assume relatively simple cyclical patterns, which may not (as demonstrated by this study's findings) fully capture the heterogeneity in symptom trajectories observed in PMDD.It is also highly unlikely that symptoms fit cleanly into an "M" or "W" pattern – some symptoms may be exacerbated during both the pre-ovulatory and perimenstrual phase. PACTS, on the other hand, detects more heterogeneous cyclical patterns.

If retaining the cosine analysis approach and if the aim of this analysis is to compare temporal patterns between groups, it is recommended that a combined model including group interaction terms be used as this would allow direct statistical testing of between-group differences in cyclical patterns.

For the analyses investigating temporal patterns of PMDD using LCGA and hierarchical clustering, it is stated that a Poisson distribution for individual PMDD symptoms was reported due to high skewness. However, some DRSP symptom domains appear to be computed as mean scores of multiple items (quasi-continuous variables), whereas others are single-item Likert ratings (ordinal). As these do not represent count data, it would be helpful to clarify the rationale for specifying a Poisson distribution.

Continuous-time vector autoregressive models were estimated exclusively for the most symptomatic cycle (defined as the cycle with the highest mean score). Please clarify the rationale for this decision. It would further strengthen the interpretation to include a sensitivity analysis assessing whether comparable results are obtained when the models are applied to the less symptomatic cycle, especially given that the PMDD sample consisted of participants with a >50% increase in premenstrual symptoms.

Please provide more detailed information regarding the correction for multiple testing applied for each research question independently, as information is only included in the Notes at the bottom of the Table S4 and Figure 6.

Results:

Please consider reporting the statistical test results for differences in sample characteristics to improve transparency in Table S1.

Figure 4: I notice there are no asymptomatic profiles, yet the discussion states that 48-59% of PMDD patients didn't report any joint, muscle pain and headache. Would it not be appropriate to exclude anyone who didn't endorse that symptom from the analysis (e.g. never higher than 2) and report what % that was? Otherwise, the results as presented suggest that all participants endorsed all symptoms.
The purpose of Table S3 is unclear and its content is somewhat misleading (e.g., suggests a high prevalence of symptom cyclicity in the control participants?). My understanding is that the percentages reported reflect the proportion of participants from each group that displayed a W pattern – is that correct? However, the amplitude of the pattern is not taken into account so the significance of these percentages is unclear. Please consider deleting or clarifying the meaning/ importance of the information presented.

Figure 5 and its connection to the results presented in Figure 4 was a little unclear on first pass. Consider adding a sentence or two at the beginning of the Figure note to explain the overarching purpose of this figure. Also, I found the two panels at the bottom to be somewhat confusing as they appear to be unrelated to the figure above – I would suggest removing them.

Table S4: Please clarify how to interpret negative effect sizes (% explained variance).

Line 808: The lagged effects were calculated across a timeframe of Δt=0-30 days. Please clarify the rationale for choosing this window given that average menstrual cycle lengths were 28 days.

Discussion:

For the temporal dynamics analyses, the results are framed as high levels of one symptom driving change in others but isn't another interpretation that underlying hormonal/ neurosteroid changes have differing lag times depending on the symptom? So it may not be symptom severity per se that is driving change in others – rather they may all be driven by the same neuroendocrine process but at different rates? Please consider using less causal language and consider discussing this possibility in the discussion section.

More comparison with Schmalenberger's PMDD subtypes paper (cited in the introduction) is warranted: though the results presented herein are quite comparable with respect to total DRSP score, the manuscript by Schmalenberger finds a higher proportion of PMDD patients reporting no anxiety or irritability; as well, Schmalenberger finds a higher proportion showing a late luteal flare in depression relative to these findings.

Lines 285-286: Headache was referred to as a diagnostic criterion for PMDD. However, it is not listed among the diagnostic criteria in the DSM-5, which should be clarified in more detail, especially in light of the conclusion of revising PMDD criteria.

The authors should acknowledge and consider discussing the Dimensional Affective Sensitivity to Hormones across the

Menstrual Cycle (DASH-MC) framework (Peters et al. Mol Psychiatry. 2025 Jan;30(1):251-262). The reported symptom trajectories appear to be mostly consistent with this framework (perhaps with the exception of depressive symptoms flaring less in the late luteal phase in the current study than the DASH framework would predict). Introducing the framework in the Introduction, and discussing these connections in the Discussion, would further strengthen the theoretical interpretation of the results.

Reviewer #2

(Remarks to the Author)
I co-reviewed this manuscript with one of the reviewers who provided the listed reports. This is part of the Nature Communications initiative to facilitate training in peer review and to provide appropriate recognition for Early Career Researchers who co-review manuscripts.

Reviewer #3

(Remarks to the Author)
Thank you for the opportunity to read and assess this important addition to the literature. There are very few well done studies on PMDD and these particular analyses are set to be a pivotal and seminal work. Indeed, PMDD and any menstrual related mood disorder has been brushed off in the medical community for decades so righting this wrong is very important by papers of this nature. Their findings of a potential for anxiety to be a driver of influence will be crucial for investigating new therapeutics. The idea that PMDD should be thought of "as a spectrum of temporally patterned symptom profiles, rather than a single homogeneous condition" needs to be acknowledged in the medical community.

Regarding methods:
• Although appropriate to use, a major limitation that should be acknowledge is using counting methods to determine/assume phase timing + no hormone measures.
• On that note, the follicular phase is highly variable, and they define it as days 6-12 for PMDD determination > were differences in length of cycle taken into consideration?
• Age range is 20-46 and there are changes in cycle structure across that timespan so was age considered in the analyses. Given this, it might be wise to do a sensitivity analyses? In addition, no mention is made of history of contraceptives or pregnancy which could also impact cycles. Was any history taken? Did it differ among groups. This is more out of curiousity.

Regarding stats:
• The use of the scaled cyclepoint variable is appreciated. And while it makes good sense to use a cosine regression for cyclical effects, were alternative models/functions considered. Please take a look at this paper and the use of GAM(M) modeling which may be a better approach as it is data-driven and works well with standardized cycle variables (Perovic et al. 2023 Scientific Reports 13, 21999 https://www.nature.com/articles/s41598-023-48628-x)
• It would be good to include the number of people in each of the clusters they identified in Table S5, where there any demographic differences between people in clusters? Similarly please give us % in lines 183-189 – not "some" "others"
• Instead of "moment-to-moment dynamics" wouldn't daily dynamics be appropriate?
• P values or effect sizes in the results would be welcome.
• Was cycle phase accounted for in the CT-VAR models? IF this is an average ("all days from the most severe cycle for each patient with PMDD was used") this may be potentially underestimating the symptom relationships during the high-symptom parts of the cycle and overestimating during the low-symptom parts (especially if more severe cycles are selected, so the differences between no-symptom and symptom parts of cycle are larger).
• Table S3 is interesting given the larger discrepancy between certain symptoms in PMDD and controls. Of course some are expected given the population but it is worth noting these for those not as familiar with PMDD (which will be a lot of people). Many of the somatic symptoms are seen in both populations but some of the others symptoms do have a perhaps notable difference (headaches, sleep, overwhelmed) which is imp to note in the discussion at least.
• IN addition to the point above, maybe it is my mistake but it appears that other symptoms in S4 show great disparities between variance accounted for in PMDD and controls that are worth mentioning: joint pain, overwhelmed, sleep, energy, maybe even appetite. But these are not mentioned in the discussion or results when talking about the data in Table S4.
• In addition to the core symptoms of PMDD and using them for drivers of change in symptoms.. I am curious were other symptoms tested? Given that there are large changes for sleep and energy maybe these symptoms are also drivers along with anxiety? Or alone? Lines 204-216. Similar question for Figure 7 – why only the core symptoms?
• Although PMDD proportions varied lines 283-286, was it also not true that the % variance accounted for with random effects was higher in PMDD for these symptoms? Joint, headache? How do you reconcile those findings?
Minor
Line 97-99.. sentence structure not quite right

Version 1:

Reviewer comments:

Reviewer #1

(Remarks to the Author)
The authors have made substantial improvements to their manuscript based on the reviewers' feedback. I find it to be much

improved and appropriate for publication.

(Remarks on code availability)

Reviewer #2

(Remarks to the Author)
I co-reviewed this manuscript with one of the reviewers who provided the listed reports. This is part of the Nature Communications initiative to facilitate training in peer review and to provide appropriate recognition for Early Career Researchers who co-review manuscripts.

(Remarks on code availability)

Reviewer #3

(Remarks to the Author)
The authors have addressed all my comments. I actually would not have changed "women" to "menstruating individuals" but would have added a definition of the term "women" used in the paper. But I am also fine with the change as well.

(Remarks on code availability)

Reviewer #4

(Remarks to the Author)
Thank you to the authors for adequately addressing all comments.

(Remarks on code availability)
Code not available

Open Access This Peer Review File is licensed under a Creative Commons Attribution 4.0 International License, which permits use, sharing, adaptation, distribution and reproduction in any medium or format, as long as you give appropriate credit to the original author(s) and the source, provide a link to the Creative Commons license, and indicate if changes were made.
In cases where reviewers are anonymous, credit should be given to 'Anonymous Referee' and the source.
The images or other third party material in this Peer Review File are included in the article’s Creative Commons license, unless indicated otherwise in a credit line to the material. If material is not included in the article’s Creative Commons license and your intended use is not permitted by statutory regulation or exceeds the permitted use, you will need to obtain permission directly from the copyright holder.
To view a copy of this license, visit https://creativecommons.org/licenses/by/4.0/

Manuscript ID: NCOMMS-25-98035

Manuscript Title: "Dynamic Psychoneuroendocrine Modeling Reveals Predictive Symptom Networks in Premenstrual Dysphoric Disorder"

Authors' response to all reviewers

Dear Referees,

We are grateful for the encouraging and constructive comments on our manuscript entitled "*Dynamic Psychoneuroendocrine Modeling Reveals Predictive Symptom Networks in Premenstrual Dysphoric Disorder*". We have revised the manuscript accordingly and provided a detailed, point-by-point response highlighted in blue. The revised-manuscript page and line numbers are given for every change; all changes are highlighted in yellow in the revised version of the manuscript.

Briefly, we have clarified and strengthened the methodological choices, incorporated the requested complementary (Phase-Aligned Cycle Time Scaled cosine regression and Generalized Additive Mixed Models) and sensitivity analyses (CT-VAR on less symptomatic cycle and secondary symptoms, and age-adjusted analyses), and expanded the discussion to better situate our findings within the literature. These revisions improve transparency and robustness, while the results and the overall conclusions remain unchanged.

The authors

REVIEWERS´ COMMENTS

Reviewer #1 (Remarks to the Author):

Overall:

Thank you for the opportunity to review this manuscript, which I read with great interest. In this study, the authors apply innovative computational and statistical approaches to examine daily affective symptom dynamics in individuals with and without premenstrual dysphoric disorder (PMDD). The approaches used are in line with our evolving understanding of PMDD as a heterogeneous disorder with important individual differences in symptom timing, severity, and specific symptoms. Overall, this is a rigorous and potentially impactful manuscript; the findings have the potential to enhance diagnostic precision and PMDD treatment individualization.

The manuscript has numerous strengths. The prospective assessment of daily symptoms across multiple menstrual cycles and including both cases and controls strengthens the reliability by providing repeated within-person measurements and an appropriate comparison group. The approach to excluding participants due to the small number of data points is appropriate, reasonable, and transparently reported. Despite these strengths, I have several suggestions for improvement, outlined below.

Authors' response: We thank Reviewer 1 for the positive assessment of the methodological rigor and clinical relevance of our study, as well as for the constructive suggestions.

Participants/ Study Procedure:

1. More detail about the two participant samples is needed (even if only in the supplement) as this may have implications for generalizability to the PMDD population as a whole. For example, what studies were participants enrolling in? Were they treatment trials? How were the studies advertised? Relatedly, how were

control participants recruited? Also, how were psychiatric comorbidities assessed (to inform participant exclusion)?

Authors' response: We thank the reviewer for this comment and agree that additional detail improves transparency and generalizability. We have expanded the Extended Methods included in the Supplement to clarify the studies from which participants were drawn, the recruitment strategies and the assessment of psychiatric comorbidities. We also revised the Supplementary Methods in the Supplementary materials on page 1-2:

"This observational, prospective study was performed as part of two double-blind randomized control trials, one on escitalopram or placebo and one on selective progesterone receptor modulator (SPRM) or placebo, conducted at Uppsala University. All procedures were approved by the Regional Ethical Review board in Uppsala (Dnr. 2016/184 and 2016/312) and the Swedish Medical Products Agency. The trials were registered as EDURA-CT 2016-001217-25 and EDURA-CT 2016-001719-19, respectively. All participants in both trials provided written informed consent. Data presented in this study only relate to the initial, screening phase of these studies. The study was not preregistered.

Upon recruitment through advertisements in local newspapers, on university campuses, and social media, participants were assessed and enrolled. Inclusion criteria were menstruating individuals aged 18-46 with regular menstrual cycles, with a confirmed PMDD diagnosis or suspecting PMDD, no ongoing pregnancy, breastfeeding or pregnancy planning, no ongoing or recent psychotropic or hormonal medication use (≥ 3-month washout), and no regular use of nicotine (e.g., smoking or using snus). Participants fulfilling these criteria were invited to a screening visit. Exclusion criteria encompassed ongoing DSM-5 disorders assessed via the Mini International Neuropsychiatric Interview (MINI) [1], past psychiatric hospitalization or suicide attempts, and severe medical conditions. Past depressive or anxiety disorders were permitted. The MINI interview as well as a Case Report Form (CRF) including questions on demographic variables reported in Supplementary Table 1, were completed during the screening." **(Supplementary materials: Lines 16-35)**

And further in the Supplementary Methods in the Supplementary materials on page 2:

"Recruitment of controls was performed similarly, through the same community-based channels, with the same inclusion and exclusion criteria, screening procedure and daily diary, except the presence of PMDD symptoms." **(Supplementary materials: Lines 49-51)**

2. It is unclear whether gender was assessed in the study. The manuscript uses "women" to refer to individuals experiencing menstrual cycles. Please clarify whether sex and gender were assessed and report this data in the sample characteristics Table if available. The authors may also consider revising the terminology throughout the manuscript to use more precise and inclusive language (e.g., "menstruating individuals").

Authors' response: Thank you for this comment. We agree with the reviewer that more inclusive language should be used. We have replaced the word "women" with "menstruating individuals" throughout the text, or sometimes just "individuals/participants" if menstruating was not needed. Biological sex was operationalized through the study eligibility criteria, which restricted inclusion to participants with regular menstrual cycles, that is with an ovulatory reproductive physiology. Gender identity was not assessed as it was beyond the scope of this study. In accordance with the journal guidelines, we have also clarified the assessment of sex on page 17 in the Methods section and in the Supplementary Methods section on page 2 of the Supplementary materials.

"Biological sex was operationalized through the study eligibility criteria, which restricted inclusion to participants with regular menstrual cycles, that is with an ovulatory reproductive physiology." **(Manuscript: Lines 472-473) (Supplementary materials: Lines 37-39)**

We further report this in Supplementary Table 1 on page 14-15 in the Supplementary materials.

3. The diagnostic threshold used for PMDD appears to require symptom increases exceeding 50%, rather than the commonly used threshold of 30% (e.g., Eisenlohr-Moul et al. Am J Psychiatry. 2017 Jan 1;174(1):51-59). The

sample may therefore encompass patients with more severe symptom presentations than the general PMDD population, which may have implications for the study results. It is suggested that the authors discuss the implications of using this strict threshold.

Authors' response: We thank the reviewer for this comment and we agree this is an important point. The strict threshold was chosen to ensure high diagnostic specificity and reduce false positives in a research context. Notably, Halbreich et al. [1] mentioned that, if the baseline is zero (or the lowest possible score, which was one in the present study), a 30% increase will still be below a disorder threshold. Using a 50% increase thus ensures that all participants are above the disorder threshold. In the revised version, we acknowledge this. In the Methods section on page 17 and in the Supplementary Methods in the Supplementary materials on page 2, we have now added the following sentence:

"Using a >50% increase of symptoms ensured that all participants were above the disorder threshold" **(Manuscript: Lines 465-466) (Supplementary materials: Lines 56-57)**

In line, we have added the following info on page 12 of the Discussion section:

"The present findings apply to a prospectively confirmed PMDD sample, using a threshold of >50% symptom increase from the follicular to the luteal phase, which may limit generalizability to less severe PMDD presentations but enhances diagnostic specificity, and reduces false positives [27]." **(Lines: 339-342)**

4. The analyses align the menstrual cycle to the onset of menses, but no information is provided regarding the assessment of ovulation. Without biological confirmation of ovulation, there is a risk of misalignment for the periovulatory and luteal phase, particularly in shorter or longer cycles. Please clarify whether ovulation was assessed and, if appropriate, discuss the potential impact of this limitation on the interpretation of symptom timing and cycle phase alignment.

Authors' response: We apologize for the lack of clarity regarding menstrual cycle tracking and thank the reviewer for pointing this out. Ovulation was not assessed and this information has now been added on page 18 of the Methods section and on page 4 and 9 of the Supplementary materials:

"biological confirmation of ovulation was not available" **(Manuscript: Line 49) (Supplementary materials: Lines 118-119 + 252-253)**

Studies have shown that the luteal phase length is relatively stable on 14 days across subjects [2, 3], and it is the follicular phase that contributes most to cycle length variability [4]. In the present study, average menstrual cycle length was 28 days (SD 2.0) and the premenstrual phase was assessed through daily reporting of first day of menstrual bleeding, and backward counting. Specifically, cycle phase was verified using bleeding records of current and subsequent cycles.

Importantly, no analysis was centered around ovulation and possible misalignment of the periovulatory phase and ovulation was thus unlikely to affect the results, as also confirmed by the virtually similar results of the complementary analyses that have been added.

Here follows a reflection regarding eventual impact of cycle misalignment for the each of the three research questions/aims:

The multilevel cosine regression analysis used as the main analysis to test research question 1 was designed to detect both premenstrual and mid-cycle symptom increases using the positive (W-shape) / negative (M-shape) cosine amplitudes. A proportional time variable was used, anchoring the cycle at the onset of menses in cycle 1 and 2, meaning that the premenstrual increases of symptoms are aligned. Nevertheless, to address the concern of possible misalignment of the periovulatory phase, we have re-estimated the cosine analysis scaling the time variable consistently with the approach of PACTS. Ovulation was set to day -15 as suggested in [5] when biological assessment of ovulation is not available. This resulted in virtually identical results as those obtained with the original approach for testing research question 1 (Figure RL1). The approach of research question 1 was therefore not affected by this limitation. In line with the other comment on PACTS, the cosine regression

models estimated with the proportional time variable presented in the original manuscript has been replaced with the new cosine regression models estimated with the PACTS time variable, and the original moved to the Supplementary materials.

The LCGA analysis used as the main analysis to test research question 2 was restricted to the symptomatic window of day -14 to day +9, where day 0 is the onset of menses, and is therefore not affected by this limitation. This approach is also consistent with the one used in Eisenlohr-Moul et al. [6].

Finally, the CT-VAR analysis used as the main analysis in research question 3 is unlikely to have been affected by the potential ovulation misalignment. This analysis models temporal dynamics between symptoms by capturing deviations from each individual's baseline (the low/no symptom state in the follicular phase). Because the model tracks day-to-day symptom changes relative to this baseline, it will detect premenstrual symptom elevation when it occurs, and is therefore not dependent on precise ovulation or cycle phase alignment.

We now explicitly acknowledge in the Discussion of each of the three main analyses, how the potential misalignment of the periovulatory phase and ovulation likely had a minor impact on the results of the cosine regression as the main results was on premenstrual dynamics. The discussion regarding the cosine regression results on page 10 of the revised manuscript now reads:

"In addition to the W-shaped pattern, the cosine approach also allowed detection of mid-cycle M-shaped symptom increases, which reflect periovulatory symptom changes. As ovulation was not biologically confirmed in the present study, cosine regression models were estimated using a phase-aligned cycle time scaled (PACTS) variable assuming ovulation at day -15 across individuals [23-25]. Because the vast majority of cosine amplitudes were positive for all symptoms in patients with PMDD (94-100%; Figure 2), reflecting predominant W-shaped patterns, potential misalignment around the periovulatory phase likely had a minor, non-systematic, impact on the results." **(Manuscript: Lines 274-282)**

Further, the discussion regarding the results of the LCGA analyses on page 11 now reads:

"As the focus of this analysis was on premenstrual dynamics and the luteal phase is assumed to be relatively stable [26, 27], the results are likely less sensitive to ovulation timing. Furthermore, these results were consistent with those found in an independent sample for total DRSP scores [21]." **(Manuscript: Lines 296-299)**

And finally, the discussion regarding the CT-VAR results on page 13 now reads:

"CT-VAR modelling, capturing deviations during the premenstrual phase from each individual's low/no-symptom state in the follicular phase, revealed temporally ordered associations among the core PMDD symptoms across adjacent observations, whereby the worsening of one symptom was associated with subsequent worsening of other symptoms; as CT-VAR models within-person symptom dynamics rather than precise cycle transitions, these findings are likely less sensitive to ovulation misalignments (Figure 6)." **(Manuscript: Lines 364-369)**

Statistical Approach:

5. It is not clear whether the research questions or the analytic plan was preregistered. Clarifying this would improve transparency. If the analyses were not preregistered, it may be more appropriate to frame the findings as exploratory.

Authors' response: We thank the reviewer for this comment, which helps us to improve the clarity of the analytic framing in the revised manuscript. We confirm that the analytical plan was not preregistered, which is now explicitly stated on page 1 in the Supplementary materials:

"The study was not preregistered" **(Supplementary materials: Lines 22-23)**

We further agree that clarifying the exploratory versus confirmatory nature of analyses improves transparency. Accordingly, we have revised the manuscript to more clearly distinguish between analyses that were guided by prior empirical research in the general population (Cosine regressions and heterogeneity modelling of premenstrual symptoms in the general population [7]) and in subjects with a 30% threshold-based PMDD (Group-based trajectory modelling of total PMDD score for some symptoms [6]), and those that were clearly exploratory in nature. Specifically, clustering of LCGA groups across symptoms; CT-VAR models exploring how the level of severity of one symptom is associated with the rate of change of another symptom (or itself) and how the level of severity of one symptom predicts the severity of another symptom (or itself) at a later specific point in time, are now explicitly described as exploratory.

This distinction has been clarified in the revised manuscript in the Methods section on page 18 and 19, and in the Supplementary Methods section in the Supplementary materials on page 4 and 7:

"This approach was guided by prior empirical research in the general population" **(Manuscript: Lines 492-493) (Supplementary materials: Lines 116-117)**

"LCGA analyses of the total DRSP score and the core PMDD symptoms were guided by prior empirical research, whereas the hierarchical clustering of temporal pattern membership across symptoms was exploratory in nature." **(Manuscript: Lines 525-528) (Supplementary materials: Lines 186-188)**

"For this exploratory analysis, a multivariate continuous time vector autoregressive (CT-VAR) model was implemented" **(Manuscript: Lines 534-535) (Supplementary materials: Lines 195-197)**

6. The purpose of the cosine analysis could be more clearly explained. Please also justify/explain the use of the cosine analysis as opposed to an approach such as PACTS (Nagpal et al. (2025). Psychoneuroendocrinology, 107584.). The cosine approach appears to assume relatively simple cyclical patterns, which may not (as demonstrated by this study's findings) fully capture the heterogeneity in symptom trajectories observed in PMDD. It is also highly unlikely that symptoms fit cleanly into an "M" or "W" pattern – some symptoms may be exacerbated during both the pre-ovulatory and perimenstrual phase. PACTS, on the other hand, detects more heterogeneous cyclical patterns.

Authors' response: We thank the reviewer for this very valuable and relevant comment. We re-estimated the cosine regression models using a PACTS time-scaling approach anchored to both menstrual onset and ovulation, following Nagpal et al. [5]. Results were virtually identical to the original analyses, indicating that findings were robust to the cycle-scaling method.

As part of the revision, we present the models estimated on the PACTS-scaled time variable in the revised manuscript but, for transparency, include the original cosine regression analyses and complementary GAMM analyses in the supplementary material. The cosine regression approach was retained because it provides interpretable individual-level estimates of cyclical symptom timing and magnitude, directly addressing Research Question 1, while clustering of symptom trajectories is specifically examined through LCGA in Research Question 2. Below, we provide a detailed answer to the comment.

Specifically, we have performed PACTS scaling of the time variable anchored to both menstrual onset and ovulation, set to day -15 for all participants, as suggested in Nagpal et al. [5] when biological measurement of ovulation is not available. We scaled time differently in the follicular and luteal phase, based on the length of the two phases for each individual, consistent with the approach of PACTS, though using a 0 to 2 scale instead of the -1 to +1 scale. We then estimated new cosine regression models based on this PACTS time variable.

As illustrated in Figure RL1 (RL stands for Response Letter), a comparison of the new analyses with the original analyses confirmed that the distributions of the estimated cosine amplitudes were virtually similar across all cosine regression models estimated with the PACTS time variable and the proportional time variable used in the original manuscript, both for the patients with PMDD and controls. Moreover, the cosine amplitudes between the models were highly correlated on the individual level (PMDD: mean r=0.98, range 0.97-0.99 across all 14 symptoms; Controls: mean r=0.97, range 0.95-1.00 across all 14 symptoms). This indicates that the

results of the cosine regressions were robust to the method of cycle scaling, with amplitude estimates being virtually identical between cycles with separate follicular and luteal phase scaling with ovulation fixed at day -15 vs. cycles that were scaled uniformly.

Figure 2, 3 and Supplementary Figure 1, as well as Supplementary Table 3 of the revised manuscript are now based on the cosine regression models estimated with the PACTS scaled time variable, and Figure and Table text have been revised accordingly. In the Methods section of the revised manuscript on page 18, as well as in the Supplementary methods section of the Supplementary materials on page 4, the following text has been added:

"A proportional time variable scaled differently in the follicular and the luteal phase based on the lengths of the two phases was used in the models. As biological confirmation of ovulation was not available, ovulation was set to day -15 across participants as suggested in Nagpal et al. [7]. One unit was added to the second cycle, so the time ranged from 0 to 2." **(Manuscript: Lines 493-496) (Supplementary material: Lines 117-120)**

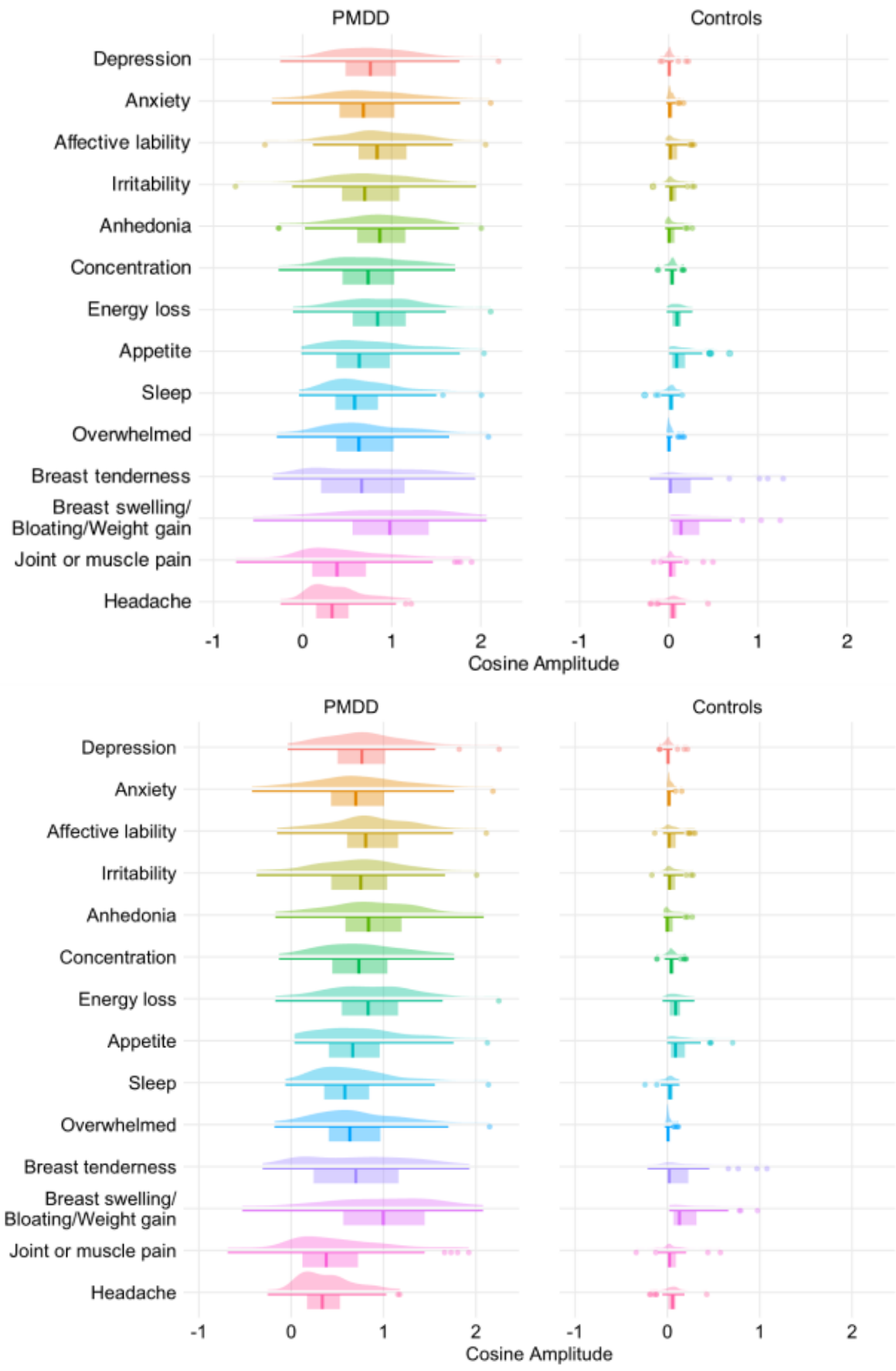

Figure RL1. Distribution of cosine amplitudes, illustrating the degree of convergence between the revised vs. the original statistical approaches.
Top: New analysis, Distribution of cosine amplitudes with PACTS scaling. The figure shows the distribution of the individual cosine amplitudes from the separate cosine regression models for each PMDD symptom for patients and controls, when estimating the models based on a PACTS scaled time variable, setting ovulation to day -15. The size of the cosine amplitude reflects the severity of the specific symptom, and the sign of the cosine amplitude reflects the timing of the symptom. **Bottom: Original analysis, Distribution of cosine amplitudes from original cosine regression models.** The figure shows the original figure of the distribution of cosine amplitudes from the cosine regression models estimated with time as a proportional variable computed based on the onset of menses and cycle length. The size of the cosine amplitude reflects the severity of the specific symptom, and the sign of the cosine amplitude reflects the timing of the symptom.

To be transparent, the original cosine regression analyses estimated with the uniformly scaled proportional time variable is now presented as an Extra analysis in Supplementary Note 1 in the Supplementary materials on page 9 (and the original figure as Supplementary Figure 2 on page 31):

"Cosine regression (proportional time variable scaled uniformly across the cycle)
Main cosine regression analyses were estimated using a proportional time variable that was scaled differently in the follicular and luteal phase depending on the lengths of the two phases, consistent with the approach of Phase-Aligned Cycle Time Scaling (PACTS) [7]. As biological confirmation of ovulation was missing, ovulation was set to day -15 across individuals. Moreover, as a secondary analysis, the cosine regression models were estimated using a proportional time variable computed by dividing cycle day by the full cycle length, representing the proportion of each cycle passed for each day of measurement [13]. As for the PACTS proportional time variable, one unit was added to the second cycle, so the time variable ranged from 0 to 2, with 0 corresponding to the first day of the first cycle, 1 to the last day of the first cycle, and 2 to the last day of the second cycle. This scaling approach yielded virtually identical results as when using a PACTS scaled time variable (Supplementary Figure 2; Supplementary Table 4), indicating that the results of cosine regressions were robust to the method of cycle scaling in this sample." **(Supplementary materials: Lines 249-261)**

The following text was added to the Methods section on page 18 of the revised manuscript:

"As a secondary analysis, cosine regression models were estimated using a proportional time variable, which was uniformly scaled across the cycle (Supplementary Note 1)." **(Lines: 497-498)**

And the following text was added to the Results section on page 6 of the revised manuscript:

"Cosine regression models estimated with a uniformly scaled proportional time variable yielded virtually identical results (Supplementary Note 1; Supplementary Figure 2; Supplementary Table 4)." **(Lines: 143-145)**

GAMM models versus cosine regression models

Regarding the use of cosine regression instead of a model like GAMM, the goal of research question 1 was to test for variability across individuals and symptoms. Specifically, cosine regressions provide coefficients indicating the direction and magnitude of cyclical effects, distributional information of cyclical change (mean level, variance), and information that can be used to test for associations in cyclical changes across variables/symptoms (e.g., are cyclical changes in mood associated with cyclical changes in somatic symptoms?). We performed cosine regression to approach this aim since it is an established method to model cyclical patterns in time-series data by fitting a sinusoidal wave, particularly suited for menstrual cycle research as it can estimate the cyclical variation for each individual [7].

Indeed, a central benefit of the cosine approach is the cosine coefficients that are generated for each individual. These can directly give us the direction and magnitude of symptom change for each individual, which can be useful to explore heterogeneity of PMDD symptoms within and across patients with PMDD and controls, as well as associations among cyclical changes across variables. In contrast, GAMM models that are used in Nagpal et al. [5], do not directly give any equivalent parameter estimates that provide this type of information or analytic opportunities. As the reviewer mentions, the cosine function does not fully capture the heterogeneity in symptom trajectory shape, as not all individuals do fit into a sinusoidal "M" or "W" pattern. However, the LCGA analyses presented in research question 2 directly address this limitation by allowing flexible, data-driven identification of distinct trajectory shapes across individuals. Unlike GAMM, which is a suitable model to fit data in a more flexible manner than cosine regression and can be used to find the average shape of symptom expression, LCGA identifies latent subgroups of individuals following similar trajectory patterns, providing both the flexibility of GAMM and the additional benefit of characterizing heterogeneity across individuals through class membership. We do not see the decision between the use of cosine-regression vs. GAMM as an either-or decision, but rather, two approaches which provide different types of information that could both be considered to gain the richest understanding possible.

As part of the revision, upon the reviewer's suggestion, we have estimated GAMM models after scaling the data according to PACTS using the R-package *menstrualcycleR,* and further estimating the hierarchical partitioning of the total explained variance (R^2) using the R-package *gam.hp* like in Nagpal et al. [5]. This was done as a complementary analysis to the cosine regression, and to see how well the average curve for each symptom fits the data, both when centering around menstrual onset and ovulation. Figure RL2 shows the

GAMM-estimated population average symptom trajectories across the standardized menstrual cycle centered on menses onset for patients with PMDD and controls, respectively. The solid line represents the population average smooth and the shaded area represents ±1SD of raw DRSP scores, reflecting individual variability around the average trajectory. Figure RL3 similarly shows the GAMM-estimated population average symptom trajectories together with ±1SD of raw DRSP scores, but with time instead centered around ovulation. Tables RL1 and RL2 include the total explained variance of each model, as well as the hierarchical partitioning of fixed and random effects. To strengthen transparency, the results of the GAMM analyses are now presented in Supplementary Note 1 of the Supplementary materials on page 9-10, Figures RL2 and RL3 have been added as Supplementary Figures 3 and 4 on page 32 of the Supplementary materials, and Tables RL1 and RL2 as Supplementary Tables 6 and 7 on page 21-22 of the Supplementary materials:

"GAMM
Generalized Additive Mixed Models (GAMM) are flexible, data-driven regression models that can capture the average non-linear patterns of change across time using smooth functions, while accounting for the nested structure of the data. This analysis was performed as a complementary analysis to the cosine regressions, which forces the data into a sinusoidal shape, to capture the observed average pattern of change across individuals.

GAMM models were estimated using two cyclic proportional time variables consistent with the PACTS approach [7] scaled differently in the follicular and luteal phase based on the lengths of the two phases, ranging from -1 to 1, one centered around menstruation onset and one around ovulation (set to day -15 across individuals). Supplementary Figures 3 and 4 show the GAMM-estimated population average symptom trajectories across the standardized menstrual cycle centered around menstruation onset and ovulation, respectively, together with ±1SD of raw DRSP scores indicating variability around the average. Supplementary Table 6 and 7 include the total explained variances (R2) for each symptom for patients with PMDD and controls, as well as the hierarchical partitioning of the proportions of fixed and random effects of the total explained variance, for the GAMM models centered around menstruation onset and ovulation, respectively. The total explained variances for the models estimated with time centered around menstruation onset was virtually identical to the total explained variances for the models estimated with time centered around ovulation across symptoms. That said, the proportions of fixed effects were consistently larger for the models centered around menstruation onset, meaning that the population average trajectory explains more variance when the cycle is centered around menses onset than around ovulation. On the other hand, the total explained variances were quite low across symptoms (range 0.15-0.32 for all models for patients with PMDD), indicating that there is substantial individual variability that the average symptom trajectories obtained with GAMM cannot explain. As for the cosine regressions, this confirmed that group-based modelling, such as performing LCGA analyses, is needed.

For controls, the GAMM models of the majority of symptoms showed ~0% fixed effects of the total explained variance, meaning that there was no consistent shared cyclical pattern across participants. This is expected as controls do not experience cyclical symptoms to the extent of patients with PMDD. Only for appetite and the physical symptoms breast tenderness and breast swelling/bloating/weight gain, some of the variance could be explained by an average cyclical pattern, which is in agreement with the findings of the cosine regressions estimated with the PACTS scaled time variable (Supplementary Table 3)." **(Supplementary materials: Lines 263-297)**

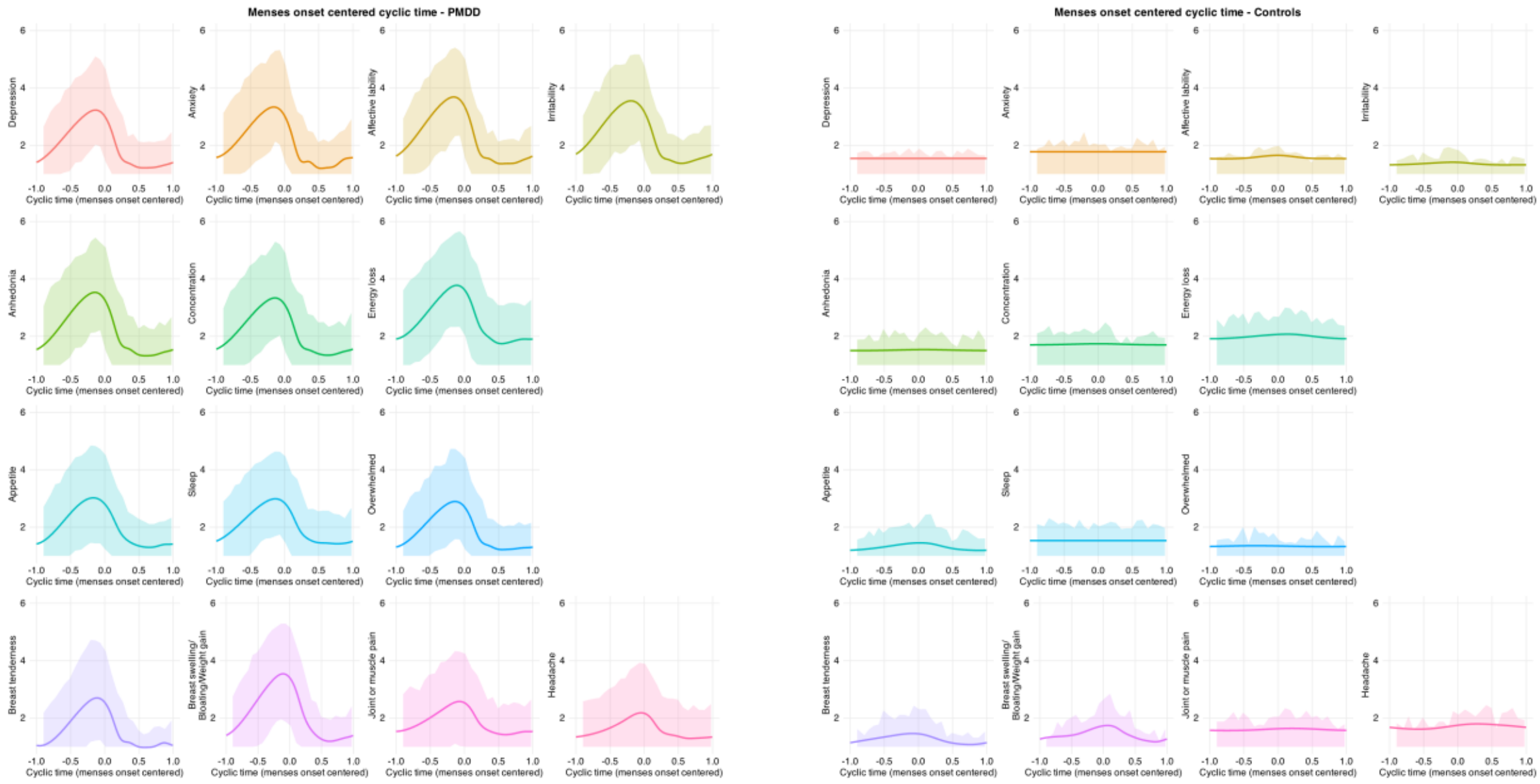

Figure RL2. GAMM estimated with PACTS scaled cyclic time centered on menses onset (Left: PMDD; Right: controls). The figure shows the average symptom trajectories for each symptom estimated with GAMM, with cyclic time centered around menses onset. The shaded area around the average is the ±1SD of raw DRSP scores.

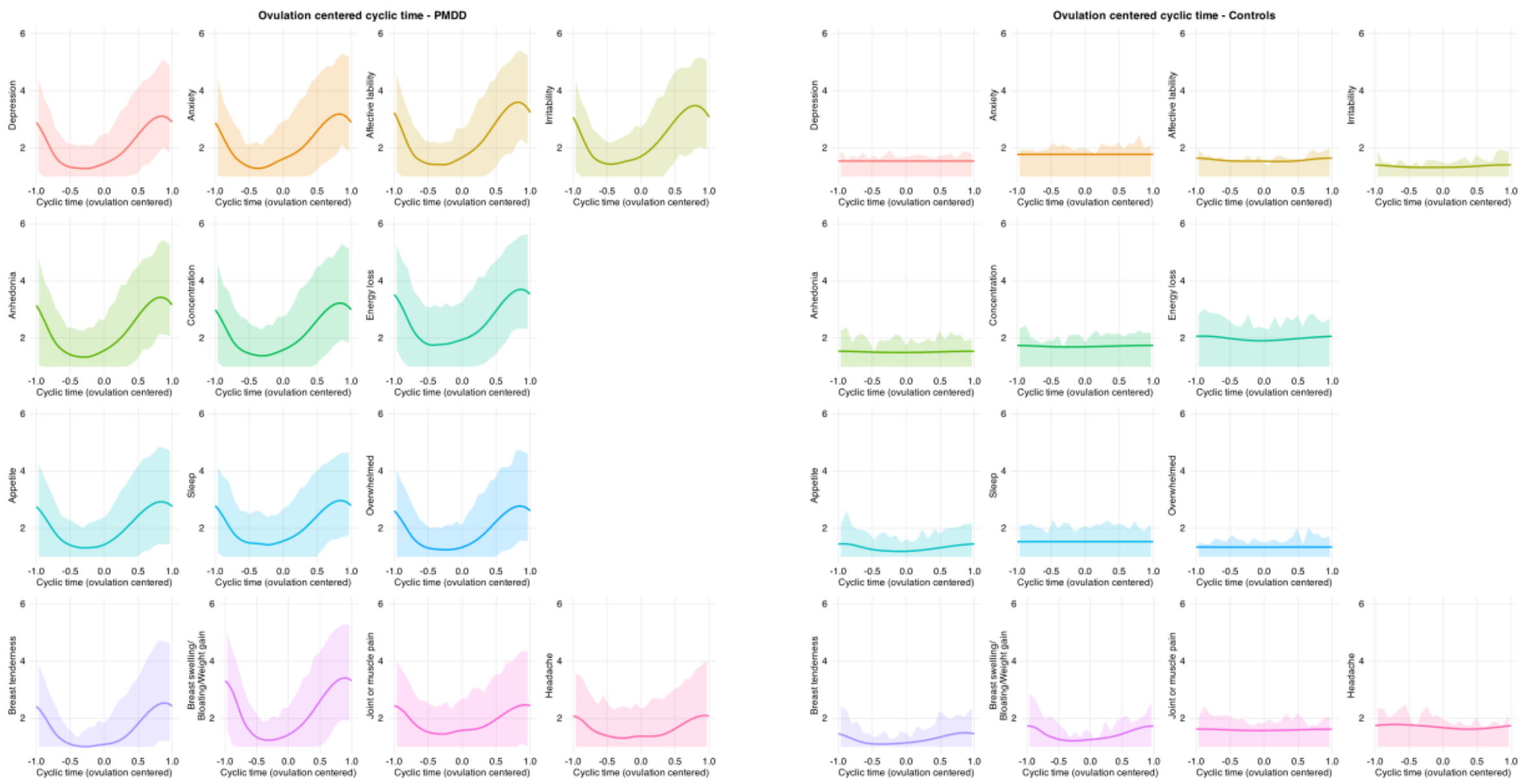

Figure RL3. GAMM estimated with PACTS scaled cyclic time centered on ovulation (Left: PMDD; Right: controls). The figure shows the average symptom trajectories for each symptom estimated with GAMM, with cyclic time centered around ovulation. The shaded area around the average is the ±1SD of raw DRSP scores.

Table RL1. Proportions of fixed and random effects of total explained variance of cyclic time centered around menses onset (GAMM).

	R^2 (adj)		Fixed effect (%)		Random effect (%)	
**Symptom**	**PMDD**	**Controls**	**PMDD**	**Controls**	**PMDD**	**Controls**
Depression	0.29	0.35	79.1	0.0	20.9	100
Anxiety	0.28	0.32	74.1	0.30	25.9	99.7
Affective lability	0.32	0.18	78.8	5.40	21.2	94.6
Irritability	0.32	0.12	71.6	6.80	28.4	93.2

	R^2 (adj)		Fixed effect (%)		Random effect (%)	
**Symptom**	**PMDD**	**Controls**	**PMDD**	**Controls**	**PMDD**	**Controls**
Anhedonia	0.28	0.19	78.3	0.70	21.7	99.3
Concentration	0.26	0.32	70.3	0.80	29.7	99.2
Energy loss	0.23	0.16	77.7	3.0	22.3	97.0
Appetite	0.25	0.27	69.8	9.30	30.2	90.7
Sleep	0.22	0.21	69.5	0.0	30.5	100
Overwhelmed	0.25	0.15	72.0	1.0	28.0	99.0
Breast tenderness	0.29	0.24	68.8	13.6	31.2	86.4
Breast swelling/ Bloating/Weight gain	0.31	0.25	80.6	24.0	19.4	76.0
Joint or muscle pain	0.19	0.46	41.5	0.50	58.5	99.5
Headache	0.15	0.33	37.2	2.0	62.8	98.0

Table RL2. Proportions of fixed and random effects of the total explained variance of cyclic time centered around ovulation (GAMM).

	R^2 (adj)		Fixed effect (%)		Random effect (%)	
**Symptom**	**PMDD**	**Controls**	**PMDD**	**Controls**	**PMDD**	**Controls**
Depression	0.29	0.35	65.6	0.0	34.4	100
Anxiety	0.29	0.32	58.1	0.10	41.9	99.9
Affective lability	0.32	0.18	64.5	4.90	35.5	95.1
Irritability	0.32	0.12	58.2	5.20	41.8	94.8
Anhedonia	0.29	0.19	65.7	0.70	34.3	99.3
Concentration	0.27	0.31	58.8	1.0	41.2	99.0
Energy loss	0.23	0.16	67.8	3.20	32.2	96.8
Appetite	0.25	0.26	61.5	9.0	38.5	90.1
Sleep	0.23	0.21	59.7	0.10	40.3	99.9
Overwhelmed	0.26	0.16	60.0	0.70	40.0	99.3
Breast tenderness	0.30	0.26	57.7	12.0	42.3	88.0
Breast swelling/ Bloating/Weight gain	0.31	0.26	73.6	23.9	26.4	76.1
Joint or muscle pain	0.20	0.46	35.9	0.50	64.1	99.5
Headache	0.15	0.33	33.2	1.60	66.8	98.4

Additionally, we estimated GAMM models with the time variable that was originally used in the cosine regressions in the original manuscript (proportional time only anchored to menses onset). The average symptom trajectories for patients with PMDD and controls estimated with GAMM are shown in Figure RL4, together with average cosine regression curves for each symptom. We clearly see the W-shape of the average symptom trajectory estimation of the GAMM models for all symptoms in the PMDD group (Figure RL4), which suggests that cosine regression provides valid estimates of the shape of change across time, comparable to the ones of GAMM, in addition to the estimates of the cosine coefficient. Thus, we believe that the Cosine regressions are a valid and meaningful way to address research question 1. Nevertheless, to strengthen transparency, the results of the GAMM analyses estimated with the proportional time only anchored to menses onset are presented in the supplement.

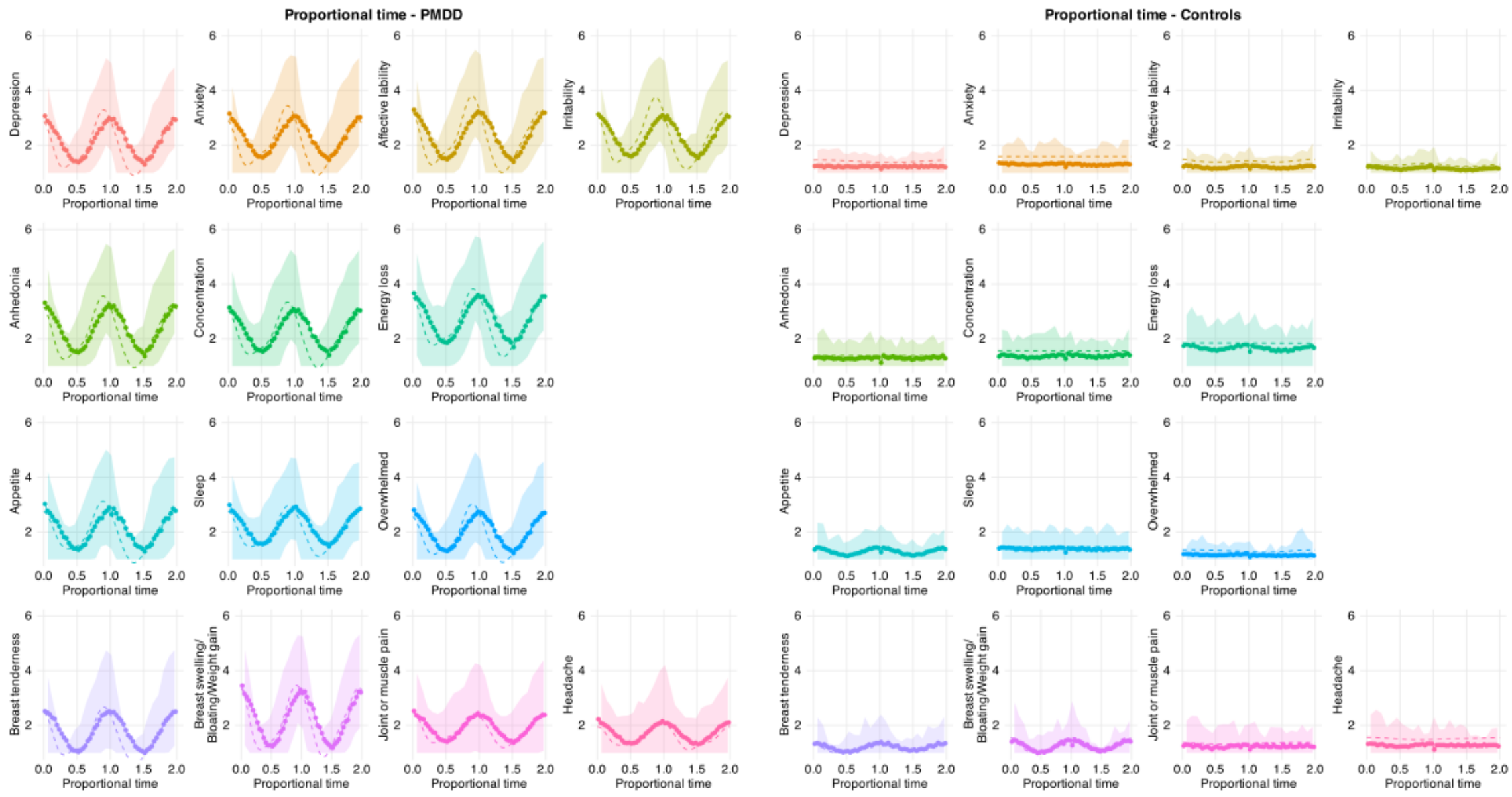

Figure RL4. Superimposed results of the two statistical approaches to facilitate comparison and illustrate their degree of overlap for the average GAMM and Cosine regression curves (Left: PMDD; Right: controls). The figure shows the average symptom trajectories for each symptom estimated with GAMM (dashed lines) and cosine regression (solid line with dots) with the original proportional time variable used in the original manuscript, anchored to menses onset only. The shaded area around the average is the ±1SD of raw DRSP scores.

Figure RL4 was added as Supplementary Figure 5 on page 33 of the Supplementary materials, and the following text was added to the Supplementary materials on page 10 under Supplementary Note 1:

"Additionally, GAMM models were estimated using a proportional time variable scaled uniformly across each cycle, ranging from 0 to 2. The average pattern across the two cycles was similar to the average W-shape that was found with cosine regression estimated with the same time variable (Supplementary Figure 5), which suggests that cosine regression provides valid estimates of the shape of change across time, comparable to the ones of GAMM." **(Supplementary materials: Lines 299-303)**

In the Methods section of the revised manuscript, the following addition was made on page 18:

"Lastly, as a complement to the cosine regression models, GAMMs were estimated to examine the explained variability and the shapes of the observed average patterns of change across symptoms (Supplementary Note 1)." **(Manuscript: Lines 510-512)**

In the Results section of the revised manuscript, the following addition was made on page 6:

"Complementary Generalized Additive Mixed Models (GAMM), using both PACTS-scaled and uniformly scaled proportional time variables, captured the average observed pattern of symptom change across the menstrual cycle and supported the robustness of the cosine regression findings (Supplementary Note 1; Supplementary Figure 3-5; Supplementary Table 6-7)." **(Manuscript: Lines 149-153)**

7. If retaining the cosine analysis approach and if the aim of this analysis is to compare temporal patterns between groups, it is recommended that a combined model including group interaction terms be used as this would allow direct statistical testing of between-group differences in cyclical patterns.

Authors' response: We thank the reviewer for this comment. We have conducted an additional cosine regression analysis, estimated with the PACTS scaled time variable, including group by cycle interaction terms to directly test between-group differences in cyclical patterns. The results statistically show that for each symptom the average cosine function is different for patients with PMDD vs. controls (Table RL3). This is now

mentioned in the Methods section on page 18 and in the Supplementary Methods on page 6 in the Supplementary materials:

"Additional cosine regression models including the interaction between time and group were estimated to statistically test whether cyclical symptom patterns differed between groups for each symptom." **(Manuscript: Lines 508-510) (Supplementary materials: Lines 154-156)**

It is now additionally mentioned in the Results section on page 6:

"Between-group differences in cyclical patterns were confirmed for all symptoms by a significant interaction between time and group (PMDD vs. control) (p<0.001 across symptoms; Supplementary Table 5)." **(Lines: 147-149)**

And table RL3 is presented as Supplementary Table 5 on page 20 of the Supplementary materials.

Table RL3. Model output of cosine regression model including the interaction between time and group. The table presents the estimates, standard errors (SE), and Bonferroni adjusted p-values for the Time*Group interactions for each symptom, reflecting whether cyclical symptom patterns differed significantly between patients with PMDD and controls for each symptom. All interaction terms were statistically significant.

Symptom	Estimate	SE	p_{adj}
Depression	0.77	0.079	<0.001***
Anxiety	0.71	0.090	<0.001***
Affective lability	0.80	0.083	<0.001***
Irritability	0.69	0.086	<0.001***
Anhedonia	0.83	0.087	<0.001***
Concentration	0.73	0.085	<0.001***
Energy loss	0.75	0.087	<0.001***
Appetite	0.58	0.085	<0.001***
Sleep	0.62	0.072	<0.001***
Overwhelmed	0.67	0.081	<0.001***
Breast tenderness	0.56	0.10	<0.001***
Breast swelling/ Bloating/Weight gain	0.74	0.11	<0.001***
Joint or muscle pain	0.42	0.088	<0.001***
Headache	0.34	0.064	<0.001***

P-values are Bonferroni adjusted for multiple comparisons.

8. For the analyses investigating temporal patterns of PMDD using LCGA and hierarchical clustering, it is stated that a Poisson distribution for individual PMDD symptoms was reported due to high skewness. However, some DRSP symptom domains appear to be computed as mean scores of multiple items (quasi-continuous variables), whereas others are single-item Likert ratings (ordinal). As these do not represent count data, it would be helpful to clarify the rationale for specifying a Poisson distribution.

Authors' response: We thank the reviewer for this valuable methodological comment. The Poisson distribution was selected based on the observed distributional properties of the symptom scores (Figure RL5). DRSP items were rated on a 1-6 scale, and showed marked right-skewed distributions with floor effects at 1, as many ratings reflect asymptomatic days during the follicular phase. Six out of fourteen symptom domains are computed as mean scores of multiple DRSP items and can thus be considered quasi-continuous variables. However, because the scores represent the average of only 2-3 DRSP items (Table S7 in the original manuscript, Supplementary Table 10 in the revised manuscript), they retained right-skewed, discrete-like properties similar to the single-item symptom ratings due to the limited number of possible values. For consistency, Poisson was therefore chosen as the distribution family for all symptoms, because it accommodates right-skewed, count-like, non-negative data.

This approach is also consistent with Eisenlohr-Moul et al. [6], who applied a count-family distribution (zero-inflated Poisson) to comparable PMDD symptom data. However, standard rather than zero-inflated Poisson was used here as scores ranged from 1-6 with no zero values. More broadly, trajectory modelling offers no single distributional family that perfectly accommodates this type of clinical data, however, Poisson is identified by Nagin & Odgers [8], as an appropriate option for non-normally distributed, count-like data in trajectory modelling. To further characterize model performance, we added entropy values for the LCGA models in Supplementary Table 8 of the revised manuscript (Table S5 in the original manuscript). Entropy, which reflects class separation based on sample size, number of classes and posterior probabilities [9], ranged between 0.82-0.94 for the symptom specific LCGA models (k=3), and was 0.99 for the total DRSP score model, indicating good class separation. This was added in the Results section on page 6:

"and entropy ranging between 0.82 and 0.99 (Supplementary Table 8), which indicates good classification" **(Lines: 161-162)**

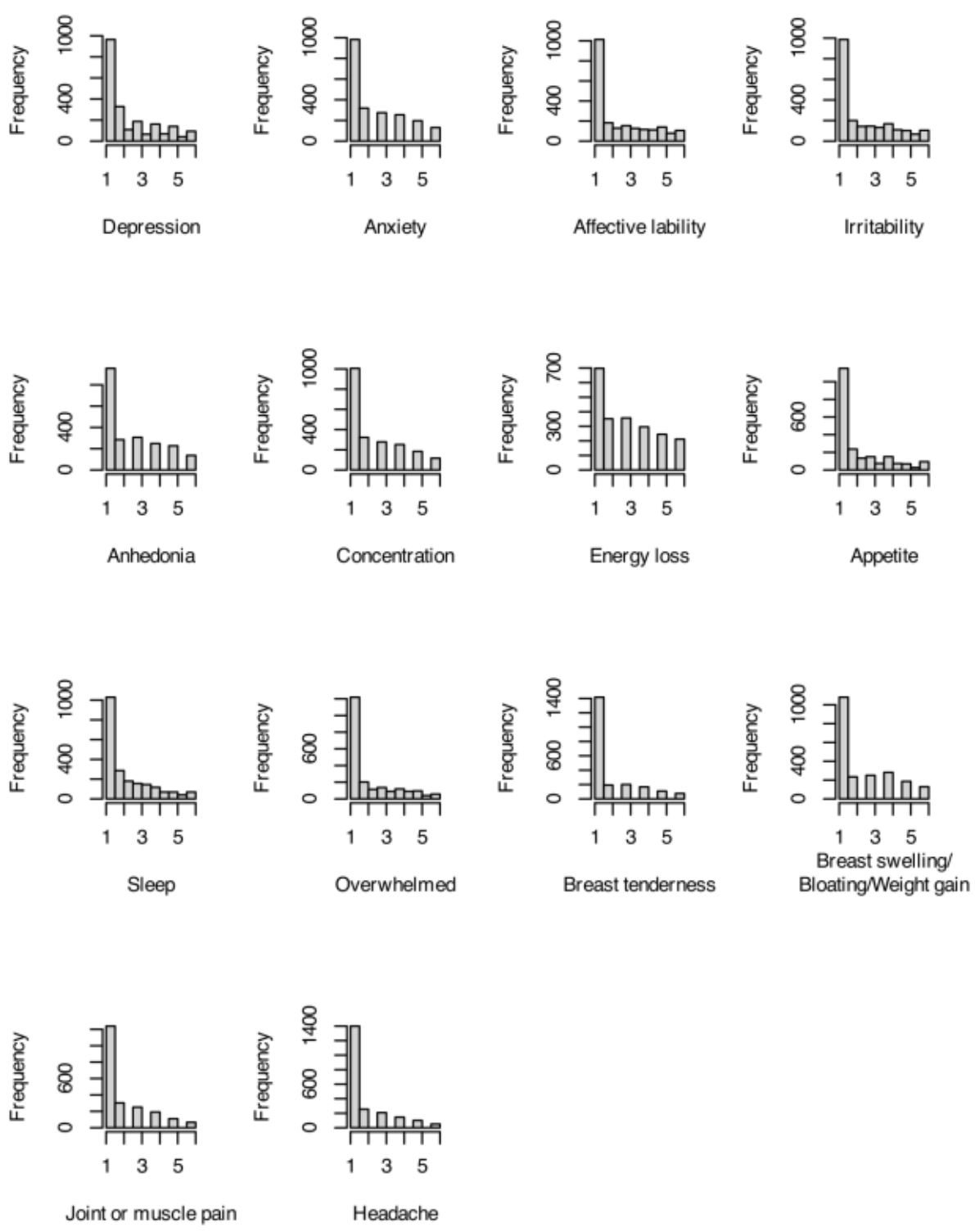

Figure RL5. Distributions of symptom scores.

9. Continuous-time vector autoregressive models were estimated exclusively for the most symptomatic cycle (defined as the cycle with the highest mean score). Please clarify the rationale for this decision. It would further strengthen the interpretation to include a sensitivity analysis assessing whether comparable results are obtained when the models are applied to the less symptomatic cycle, especially given that the PMDD sample consisted of participants with a >50% increase in premenstrual symptoms.

Authors' response: We appreciate the reviewers' suggestion. The rationale was to maximize signal-to-noise ratio and ensure sufficient variability for dynamic modeling. We have, upon the suggestion from the reviewer, estimated a continuous time vector autoregressive model using the less symptomatic cycle. The key findings remained consistent with the original CT-VAR model (Figure RL6), thus suggesting that the original CT-VAR estimation of symptom dynamics are robust across menstrual cycles.

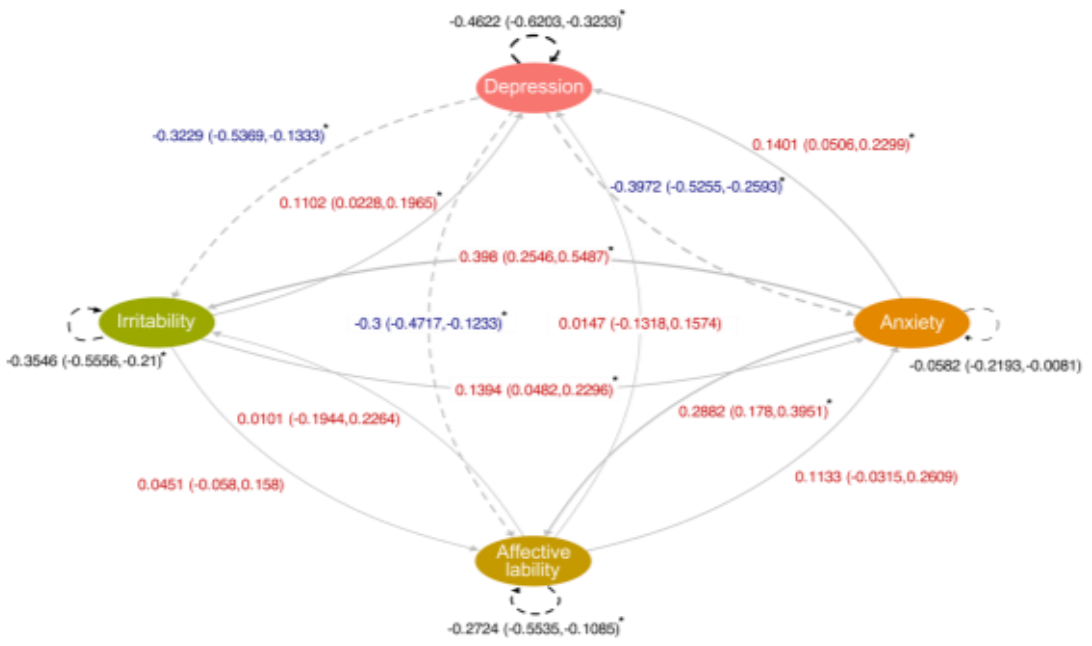

Figure RL6. Dynamic network of the core symptoms estimated with the less severe cycle. Solid lines represent positive effects, and dashed lines represent negative effects. The network is derived from one CT-VAR model including all four core symptoms of PMDD, in which temporal dynamics between all days from the less severe cycle for each patient with PMDD was estimated. * Indicates effects that were significant after correction for multiple testing using the Benjamini-Hochberg correction. See Figure 6 of the revised manuscript for a full description of model parameters and interpretation.

This sensitivity analysis has now been included in Supplementary Note 1 of the Supplementary materials on page 11:

"The main CT-VAR model was estimated using the most symptomatic cycle for each individual. The rationale was to maximize signal-to-noise ratio and to ensure sufficient variability for dynamic modelling. However, as a sensitivity analysis, an additional CT-VAR model was estimated using the less symptomatic cycle for each individual. The less symptomatic cycle was the cycle with the lowest mean sum score of the four core symptoms over five consecutive days in the luteal phase. The key findings of the main CT-VAR analysis of the most symptomatic cycle were confirmed by the sensitivity analysis (Supplementary Figure 6): Depression showed a faster rate back to the low/no symptom state compared to the other symptoms (more negative drift parameter), and was associated with a dampening effect on the other core symptoms' rate of change. Anxiety was significantly associated with increases in the rate of change of the other three core symptoms, and together with irritability formed a feedback loop in which both symptoms were associated with increases in the other symptom's rate of change. This suggests that the key CT-VAR findings are robust across menstrual cycles." **(Lines: 306-318)**

The Methods section on page 19 was further revised and now reads:

"For this exploratory analysis, a multivariate continuous time vector autoregressive (CT-VAR) model was implemented [45], using the most symptomatic cycle, and each individual's low/no-symptom state in the follicular phase as the equilibrium. A sensitivity analysis using the less symptomatic cycle was performed to assess the robustness of the findings" **(Lines: 534-539)**

Lastly, it has been mentioned in the Results section of the revised manuscript on page 9:

"A sensitivity analysis using the less symptomatic cycle yielded similar findings, suggesting robustness of the estimation of symptom dynamics across menstrual cycles (Supplementary Note 1; Supplementary Figure 7)." **(Lines: 236-238)**

Figure RL6 has been included in the Supplementary materials as Supplementary Figure 7 on page 35.

10. Please provide more detailed information regarding the correction for multiple testing applied for each

research question independently, as information is only included in the Notes at the bottom of the Table S4 and Figure 6.

Authors' response: We fully agree with the reviewer that this is an important point that should be more explicitly presented. In research question 1, correction for multiple comparisons using Bonferroni correction was performed on the p-values of the fixed and random effects of the cosine regression models, and Benjamini-Hochberg correction was further used for correction for multiple testing of the correlation analysis of cosine amplitudes. In research question 2 correction for multiple testing was not applicable, as LCGA does not rely on null hypothesis significance testing but rather on model fit criteria such as BIC and AIC for model selection. In research question 3 correction for multiple testing using Benjamini-Hochberg correction was performed on the generated p-values for the drift parameters, presented in Figure 6 of the original manuscript. We have now explicitly described and clarified the correction procedures for each research question separately in the Methods section:

For cosine regressions on page 18:
"Bonferroni correction was applied to the fixed and random effects of the cosine regression models, and Benjamini-Hochberg correction was applied to the correlation analyses, to adjust for multiple comparisons across symptoms." **(Lines: 504-507)**

For LCGA, model selection was clarified on page 19:
"The number of temporal patterns was determined based on model fit criteria (BIC and AIC) and temporal pattern percentages (>5%)." **(Lines: 520-521)**

For CT-VAR models on page 20:
"Significance of the drift parameters was determined from the 95% confidence intervals and corrected for multiple comparisons using Benjamini-Hochberg correction." **(Lines: 551-553)**

And further added in the Supplementary Methods in the Supplementary materials:

For cosine regressions on page 5:
"Bonferroni correction was applied separately to the estimated p-values of the fixed and random effects of the cosine regression models to adjust for multiple comparisons across symptoms." **(Lines: 145-147)**
and
"Benjamini-Hochberg correction for multiple testing was applied to adjust for the number of pairwise correlations tested across symptoms (91 comparisons for 14 symptoms)." **(Lines: 151-152)**

And for CT-VAR models on page 8 it now reads:
"The drift parameters and their 95% confidence intervals were visualized as a dynamic network. Significance was determined from the 95% confidence intervals. Benjamini-Hochberg correction was applied to the drift parameters to adjust for multiple comparisons across the autoregressive and cross-symptom effects." **(Lines: 226-229)**

Results:

11. Please consider reporting the statistical test results for differences in sample characteristics to improve transparency in Table S1.

Authors' response: We thank the reviewer for this comment. Following this suggestion, we have included statistical comparisons for group difference by adding a column to Supplementary Table 1 in the revised manuscript. Please note that the demographic differences between groups are unlikely to affect the findings (please see also the reply to reviewer 3 regarding age), as controls were only included in research question 1 where separate cosine regression models were run, rather than directly comparing groups.

12. Figure 4: I notice there are no asymptomatic profiles, yet the discussion states that 48-59% of PMDD patients didn't report any joint, muscle pain and headache. Would it not be appropriate to exclude anyone who didn't endorse that symptom from the analysis (e.g. never higher than 2) and report what % that was? Otherwise, the results as presented suggest that all participants endorsed all symptoms.

Authors' response: We thank the reviewer for this comment and apologize for the confusion. The statement in the discussion referred to the fact that the moderate late luteal pattern identified by the LCGA analysis for both joint or muscle pain and headache included the largest proportion of patients with PMDD (~48% and ~59%, respectively), compared to the other two temporal patterns, and showed low symptom severity scores (described as "asymptomatic" in the original manuscript). To clarify this, we have revised the discussion on page 12 to better describe the symptom severity levels represented by this pattern:

"Interestingly, the majority of patients with PMDD displayed a moderate late luteal temporal pattern characterized by very low symptom severity for joint or muscle pain and headache (48% and 59%, respectively). These findings suggest that joint or muscle pain may be less central to PMDD than currently assumed. They may also warrant reconsideration of how physical symptoms are weighted within PMDD diagnostic frameworks and commonly used symptom rating tools such as the DRSP [26], which includes additional symptoms such as headache alongside joint or muscle pain." **(Lines: 323-329)**

We did not exclude participants with low endorsement of a specific symptom, as one of the aims of the study was to characterize heterogeneity in symptom temporal patterns. Notably, a participant may show very low severity levels for one symptom (e.g. "joint or muscle pain") while displaying clinically relevant symptom increases in others. To avoid further misunderstanding, we also clarified throughout the manuscript that, for brevity, we used the same temporal pattern labels across symptoms (i.e., Severe full luteal, Severe late luteal, and Moderate late luteal), although some physical symptoms the LCGA analysis included a mild/asymptomatic temporal pattern that deviated from these generalized labels.

13. The purpose of Table S3 is unclear and its content is somewhat misleading (e.g., suggests a high prevalence of symptom cyclicity in the control participants?). My understanding is that the percentages reported reflect the proportion of participants from each group that displayed a W pattern – is that correct? However, the amplitude of the pattern is not taken into account so the significance of these percentages is unclear. Please consider deleting or clarifying the meaning/ importance of the information presented.

Authors' response: We thank the reviewer for this comment. The authors agree that Table S3 of the original manuscript can be misleading, as this table only report the proportions of positive cosine amplitudes (i.e., W-shape, premenstrual increase of symptom), without taking magnitude of symptom into consideration. We have therefore decided to remove Table S3 in the revised version of the manuscript upon the reviewer's suggestion.

14. Figure 5 and its connection to the results presented in Figure 4 was a little unclear on first pass. Consider adding a sentence or two at the beginning of the Figure note to explain the overarching purpose of this figure. Also, I found the two panels at the bottom to be somewhat confusing as they appear to be unrelated to the figure above – I would suggest removing them.

Authors' response: We thank the reviewer for this comment and for drawing our attention to the unclarity regarding the presentation of these two Figures. To clarify, the heatmap on top of Figure 5 of the original manuscript shows the temporal pattern membership across symptoms for each participant (columns) clustered into three clusters with most similar combinations found in the two-dimensional hierarchical clustering. Rows show PMDD symptoms clustered into five clusters based on similarities of temporal pattern membership. The bottom panels in Figure 5 of the original manuscript are examples of one participant displaying different temporal patterns for different symptoms, and one participant showing the same temporal pattern for most symptoms. We have revised the legend of Figure 5 on page 30 to clarify the connection between Figure 4 and 5 by adding the following sentence to the beginning of the legend:

“Building on the temporal patterns identified for each symptom using LCGA (Figure 4), Figure 5 illustrates how these patterns cluster across symptoms and across patients with PMDD, revealing the similarity and variability of temporal pattern membership across symptoms within and across individuals.” **(Lines: 742-745)**

We have further revised Figure 5 to better connect the two panels at the bottom to the top of the figure illustrating the hierarchical clustering (as in Figure RL7), and revised the figure legend accordingly.

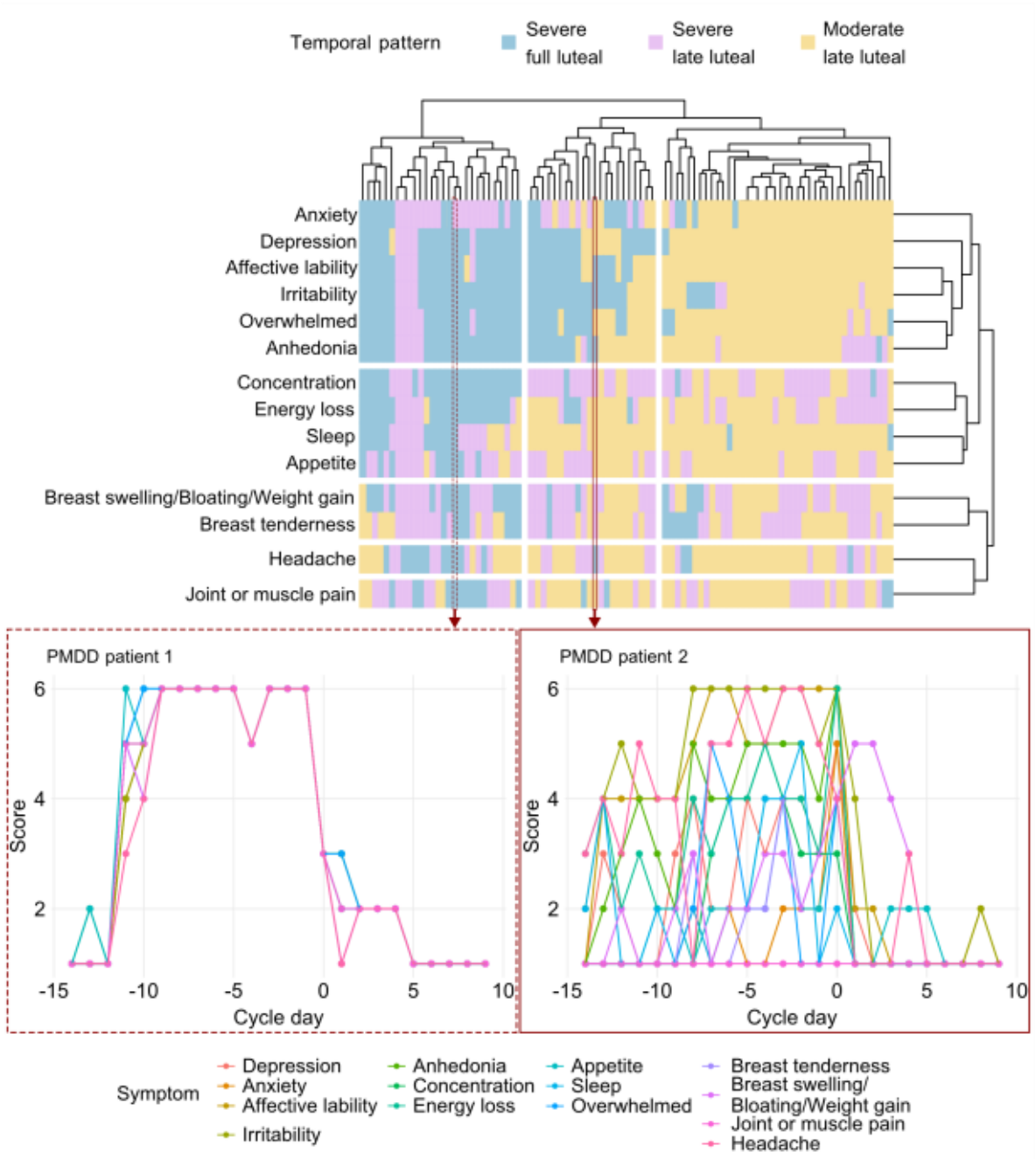

Figure RL7. Clusters of temporal patterns by symptoms of PMDD. The figure shows the clustering of temporal pattern membership across symptoms, and clustering of symptoms according to temporal pattern membership across patients. This is the revised version of Figure 5 of the original manuscript.

Additionally, we added the following text in the results section on page 7:

“Given the symptom-specific variation in temporal patterns, it was examined whether symptoms clustered according to shared temporal trajectories within individuals.” **(Lines: 193-194)**

15. Table S4: Please clarify how to interpret negative effect sizes (% explained variance).

<u>Authors’ response:</u> We thank the reviewer for this comment and agree that further clarification is needed. The negative values in Table S4 of the original manuscript arise from the way variance explained was calculated in the simplest model (referred to as Model 1). Negative effect sizes occurred for some symptoms for controls, which means that when adding the cosine function it explains the variance worse than the baseline model. This occurs because Model 1 included a linear time trend, meaning that the baseline already accounted for some time-related variance. For a more consistent comparison of effect sizes to each person’s baseline, we therefore recomputed Model 1 as a random intercepts-only model, so that the effect sizes solely reflect the proportion of within-person variance accounted for by the cosine function compared to each person’s mean level. We also recomputed the ICC-values and the effect sizes of the fixed and random effects of the cosine function and updated the table, which is Supplementary Table 3 in the revised manuscript and Table RL4 in this letter. As reviewer 1 also requested to replace the uniformly scaled proportional time variable with a phase-aligned cycle time scaled (PACTS) variable, the values in the revised supplement reflect the models estimated with the PACTS scaled time variable. Notably, the values were robust as they are virtually similar, and the rank order of the symptoms remained the same for all effects.

Table RL4. Re-computed effect sizes explaining the variance between and within patients with PMDD and controls.

Symptom	Random intercepts (Model 1) (ICC)		Fixed effects of cycle (Model 2) (% variance explained)		Random effects of cycle (Final model) (% variance explained)	
	PMDD	Control	PMDD	Control	PMDD	Control
Depression	0.17*	0.38*	18 %***	0.04 %	25 %***	3.0 %
Anxiety	0.17*	0.41*	13 %***	0.11 %	21 %***	1.3 %
Affective lability	0.15*	0.22*	17 %***	0.89 %***	24 %***	4.9 %***
Irritability	0.15*	0.21*	14 %***	0.70 %**	21 %***	5.0 %***
Anhedonia	0.13*	0.25*	16 %***	0.18 %	23 %***	2.0 %*
Concentration	0.20*	0.36*	14 %***	0.22 %	21 %***	2.3 %
Energy loss	0.16*	0.28*	15 %***	0.61 %**	21 %***	1.8 %**
Appetite	0.25*	0.37*	16 %***	3.6 %***	24 %***	8.9 %***
Sleep	0.24*	0.38*	15 %***	0.04 %	21 %***	2.5 %
Overwhelmed	0.20*	0.29*	16 %***	0.09 %	24 %***	1.4 %
Breast tenderness	0.22*	0.41*	18 %***	5.3 %***	32 %***	24 %***
Breast swelling/ Bloating/ Weight gain	0.19*	0.27*	23 %***	7.2 %***	33 %***	19 %***
Joint or muscle pain	0.36*	0.49*	9.5 %***	0.34 %	22 %***	4.4 %***
Headache	0.27*	0.37*	6.3 %***	0.17 %	12 %***	4.0 %**

***: p < 0.001; **: p < 0.01; *: p < 0.05
p-values for fixed and random effects were corrected for multiple comparisons using Bonferroni correction.

16. Line 808: The lagged effects were calculated across a timeframe of Δt=0-30 days. Please clarify the rationale for choosing this window given that average menstrual cycle lengths were 28 days.

Authors' response: We thank the reviewer for this comment and the need for clarification. The choice of Δt was based on the fact that the majority of participants had an observed cycle length of 24-30 days (79%) in the most symptomatic cycle used for the CT-VAR model. Further, it was not expected that symptoms would predict each other or themselves from cycle day one to the last day of the cycle, which is why we chose a value of 30 days reflecting the majority of participants rather than the maximum observed cycle length.

However, the authors also want to clarify that the choice of Δt does not affect the results but rather gives the possibility to explore predictive abilities across time differences defined in the chosen timeframe, which in our original manuscript was 0-30 days. In Figure 7 of the original manuscript, it can be seen that the strongest effects occurred within time differences of 3-5 days for all symptoms, and that around time differences of 15-20 days there were no effect for the predictive ability of any symptom, meaning that the choice of 30 vs the average menstrual cycle length of 28 is negligible. To further clarify, Figure RL8 shows the lagged effects of the core symptoms of PMDD but instead across a timeframe of Δt=0-28 days, indicating the same results. To improve clarity, while leaving the results unaltered, we replaced the original output with this revised one in the revised manuscript as Figure 7 on page 32, while the results remain unchanged.

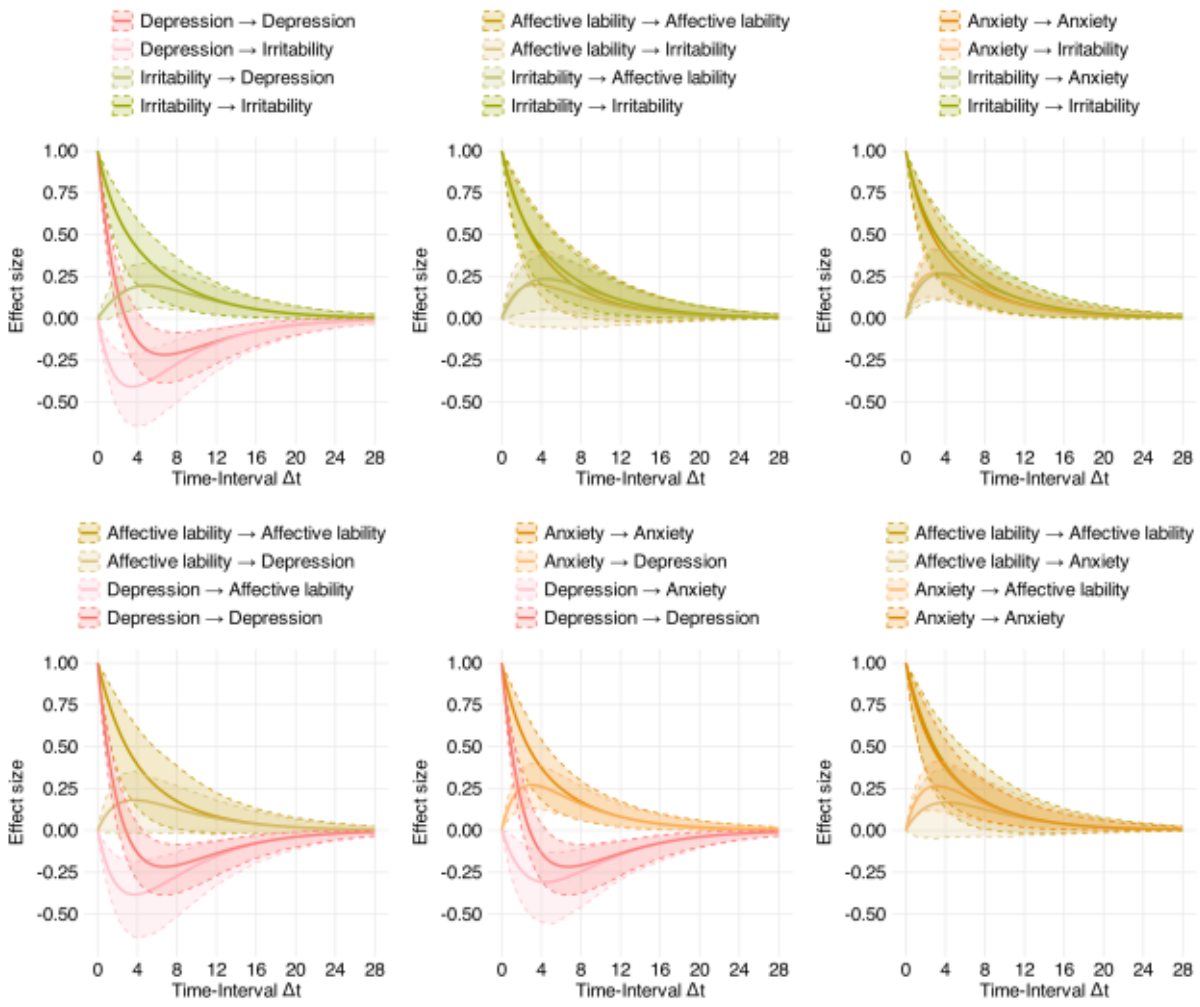

Figure RL8. Lagged effects of the core symptoms of PMDD. The figure depicts how each of the core symptoms of PMDD will predict the future values of itself and the other core symptoms depending on the time-interval (days) between measurements across a range of time differences between 0-28 days. The graphs are derived from one CT-VAR model including all four symptoms, in which all days from the most symptomatic cycle for each patient with PMDD was used. A regular time variable in days was used in the model, and the low/no symptom state was used as equilibrium.

Discussion:

17. For the temporal dynamics analyses, the results are framed as high levels of one symptom driving change in others but isn't another interpretation that underlying hormonal/ neurosteroid changes have differing lag times depending on the symptom? So it may not be symptom severity per se that is driving change in others – rather they may all be driven by the same neuroendocrine process but at different rates? Please consider using less causal language and consider discussing this possibility in the discussion section.

Authors' response: We thank the reviewer for this important comment that helped us to strengthen our manuscript. We agree that CT-VAR modelling does not imply causation, but rather statistical temporal associations. Upon the reviewer's suggestion we have revised the language throughout the abstract, introduction, results, discussion, and methods section in the manuscript and the extended methods section in the supplementary materials, to avoid causal claims. Specifically, causal language has been replaced with associational language (e.g., 'associated with'), and claims have been stated using terms such as 'potential' and 'suggesting'. Additionally, 'causal ordering' and 'causal hierarchies' have been replaced with 'temporal ordering' and 'temporal hierarchies' to more accurately reflect the CT-VAR output.

Additionally, the following text has been added to the discussion section on page 14, to explicitly discuss the alternative explanation suggested by the reviewer:

"While temporal associations in the CT-VAR models are described in terms of symptom-to-symptom influences for interpretive clarity, these findings cannot be interpreted as evidence of direct causal relationships between symptoms. An alternative explanation is that observed temporal dependencies reflect a shared underlying neuroendocrine process, such as cyclical fluctuations in ovarian hormones and neurosteroids, with different symptoms exhibiting distinct sensitivity profiles and temporal lags in response to these upstream biological changes. Under this interpretation, and consistent with the GABAergic sensitivity discussed above, anxiety may appear to precede other symptoms because affective and threat-related systems are closely linked to rapid modulation of GABAergic-mediated inhibitory tone by neurosteroids such as allopregnanolone, which has been shown to influence both amygdala-based threat sensitivity and central stress–arousal regulation via effects on HPA-axis reactivity and neural excitability [31-34] . This has also been recently corroborated by PMDD having been associated with altered GABA A receptor subunit plasticity (especially δ-subunits) [35]." **(Lines: 391-403)**

18. More comparison with Schmalenberger's PMDD subtypes paper (cited in the introduction) is warranted: though the results presented herein are quite comparable with respect to total DRSP score, the manuscript by Schmalenberger finds a higher proportion of PMDD patients reporting no anxiety or irritability; as well, Schmalenberger finds a higher proportion showing a late luteal flare in depression relative to these findings.

Authors' response: We have followed the reviewer's suggestion and have expanded comparison and discussed discrepancies in the revised version of the manuscript. As the reviewer mentions, the results for the total DRSP score corroborated the results found by Eisenlohr-Moul et al. [6]. However, when further examining temporal patterns of the individual symptoms, there are some discrepancies between Eisenlohr-Moul et al and the results presented in this study. In Eisenlohr-Moul et al., the largest proportion for depression was found in the trajectory "severe premenstrual week with late offset", however, such trajectory was not found in the present study, but rather a severe full luteal pattern (including 42.2% of participants), and a severe late luteal pattern with late offset (only including 5.6% of participants). Most participants were, however included in the moderate late luteal pattern for depression (52.2% of participants). Regarding anxiety, we found very similar proportions and trajectory shape of the moderate late luteal pattern (46.7% compared to 47.2% in Eisenlohr-Moul et al.), similarly the severe late luteal pattern with late offset (28.9% of participants), displayed an earlier onset (~day -11) than the other core symptoms and is thus comparable to the trajectory "severe full luteal phase with late offset" reported by Eisenlohr-Moul et al., which had a symptom onset around day -11 as well and included a comparable proportion of 21.6%. However, as the reviewer mentions, a proportion of participants were found to not have any symptoms of both Anxiety and Irritability (31.2% and 35%, respectively). This was not found in our sample, which may reflect the more stringent definition of PMDD used in the present study [1], as discussed in one of the previous questions from the reviewer. Finally, comparing the results of muscle pain, results were very similar to the results found in Eisenlohr-Moul et al., where one asymptomatic group was found (47.8% of participants in our sample vs 35% in Eisenlohr-Moul et al.), a mild premenstrual week pattern (35.6% of participants in our sample vs 30% in Eisenlohr-Moul et al.), and finally a moderate full luteal pattern (16.7% of participants in our sample vs 35% in Eisenlohr-Moul et al.). The discrepancies between the two samples could be due to the use of different tools to assess daily symptom ratings, the stricter threshold for symptom increase between the follicular and luteal phase used in our study (50% increase), compared to the lower threshold used in Eisenlohr-Moul et al., which ensures high specificity and clinically relevant PMDD in all included participants in our study [1]), sociocultural differences, or other factors.

On page 12 in the Discussion of the revised manuscript we also added the following paragraph, which is integrated with the discussion of the DASH-MC framework, mentioned in a further question by the reviewer:

"The current findings can be interpreted in light of aspects of the Dimensional Affective Sensitivity to Hormones across the Menstrual Cycle (DASH-MC) framework [22], supporting heterogeneity in temporal symptom patterns and hormonal sensitivity, consistent with prior work in non-clinical samples [18]. Irritability showed a predominant severe full luteal pattern, which aligns with the luteal-onset sensitivity dimension and previous findings [21]. Notably, anxiety emerged as a central symptom in the CT-VAR analyses despite not being central in DASH-MC, suggesting that existing dimensional models may not fully capture dynamic symptom interactions in PMDD. Depression showed partial correspondence with prior hypotheses, here predominantly luteal rather than perimenstrual timing [21, 22]. Furthermore, unlike [21], the absence of asymptomatic patterns for anxiety and irritability may reflect the more stringent symptom threshold used here [27]." **(Lines: 330-339)**

19. Lines 285-286: Headache was referred to as a diagnostic criterion for PMDD. However, it is not listed among the diagnostic criteria in the DSM-5, which should be clarified in more detail, especially in light of the conclusion of revising PMDD criteria.

Authors' response: We apologize for the lack of clarity. As headache is included in the DRSP [8], a widely used prospective tool for daily ratings in PMDD research, our intention was not to discuss headache as a diagnostic criterion, but rather as a clinically relevant symptom assessed within the DRSP framework. The reviewer is correct and this statement in the discussion section on page 12 has been revised and now reads:

"These findings suggest that joint or muscle pain may be less central to PMDD than currently assumed. This may also warrant reconsideration of how physical symptoms are weighted within PMDD diagnostic frameworks

and commonly used symptom rating tools such as the DRSP [26], which includes additional symptoms such as headache and joint or muscle pain." **(Lines: 325-329)**

The statement in the conclusion on page 16 has further been revised and now reads:

"these findings can inform future refinement of the PMDD diagnostic criteria and tools for daily symptom rating, and pave the way for temporally targeted, evidence-based treatments tailored to individual symptom profiles to prevent symptom escalation." **(Lines: 446-449)**

20. The authors should acknowledge and consider discussing the Dimensional Affective Sensitivity to Hormones across the Menstrual Cycle (DASH-MC) framework (Peters et al. Mol Psychiatry. 2025 Jan;30(1):251-262). The reported symptom trajectories appear to be mostly consistent with this framework (perhaps with the exception of depressive symptoms flaring less in the late luteal phase in the current study than the DASH framework would predict). Introducing the framework in the Introduction, and discussing these connections in the Discussion, would further strengthen the theoretical interpretation of the results.

Authors' response: We appreciate the reviewer's suggestion and have revised the manuscript accordingly. The Introduction section, has now been revised on page 4:

"Emerging theoretical work has further highlighted the potential relevance of heterogeneity in menstrual cycle-related affective sensitivity [22]." **(Lines: 82-84)**

Integrated discussion of findings with the framework has further been added to the Discussion section on page 12:

"The current findings can be interpreted in light of aspects of the Dimensional Affective Sensitivity to Hormones across the Menstrual Cycle (DASH-MC) framework [22], supporting heterogeneity in temporal symptom patterns and hormonal sensitivity, consistent with prior work in non-clinical samples [18]. Irritability showed a predominant severe full luteal pattern, which aligns with the luteal-onset sensitivity dimension and previous findings [21]. Notably, anxiety emerged as a central symptom in the CT-VAR analyses despite not being central in DASH-MC, suggesting that existing dimensional models may not fully capture dynamic symptom interactions in PMDD. Depression showed partial correspondence with prior hypotheses, here predominantly luteal rather than perimenstrual timing [21, 22]. Furthermore, unlike [21], the absence of asymptomatic patterns for anxiety and irritability may reflect the more stringent symptom threshold used here [27]." **(Lines: 330-339)**

In the Discussion section on page 13 the following was added to further relate the LCGA findings to the CT-VAR findings:

"This is consistent with the LCGA findings, where the severe full luteal and severe late luteal patterns together accounted for the majority of participants, both showing an earlier luteal onset compared to the other symptoms, suggesting that anxiety may emerge earlier in the luteal phase." **(Lines: 372-375)**

Reviewer #2 (Remarks to the Author):

I co-reviewed this manuscript with one of the reviewers who provided the listed reports. This is part of the Nature Communications initiative to facilitate training in peer review and to provide appropriate recognition for Early Career Researchers who co-review manuscripts.

Authors' response: Thank you very much.

Reviewer #3 (Remarks to the Author):

Thank you for the opportunity to read and assess this important addition to the literature. There are very few well done studies on PMDD and these particular analyses are set to be a pivotal and seminal work. Indeed,

PMDD and any menstrual related mood disorder has been brushed off in the medical community for decades so righting this wrong is very important by papers of this nature. Their findings of a potential for anxiety to be a driver of influence will be crucial for investigating new therapeutics. The idea that PMDD should be thought of "as a spectrum of temporally patterned symptom profiles, rather than a single homogeneous condition" needs to be acknowledged in the medical community.

Authors' response: We thank Reviewer 3 for these positive comments, for recognizing the novelty and significance of our findings, and for the valuable comments on data analysis that contributed significantly to improve the paper.

Regarding methods:
1. • Although appropriate to use, a major limitation that should be acknowledge is using counting methods to determine/assume phase timing + no hormone measures.

Authors' response: We appreciate this comment. As mentioned in the response to reviewer 1, we now clarified in the Methods section on page 18 and on page 4 and 9 of the Supplementary materials that ovulation was not confirmed:

"biological confirmation of ovulation was not available" **(Manuscript: Line 495) (Supplementary materials: Lines 118-119 + 252-253)**

However, as also mentioned in the response to reviewer 1, studies have shown that the luteal phase length is relatively stable on 14 days across subjects [2, 3], and it is the follicular phase that contributes most to cycle length variability [4]. In the present study, average menstrual cycle length was 28 days (SD 2.0) and the premenstrual phase was assessed through daily reporting of first day of menstrual bleeding, and backward counting. Specifically, cycle phase was verified using bleeding records of current and subsequent cycles.

Importantly, no analysis was centered around ovulation and possible misalignment of the periovulatory phase and ovulation was thus unlikely to affect the results, as also confirmed by the results of the complementary analyses that have been added.

Here follows a reflection regarding eventual impact of cycle misalignment for the each of the three research questions:

The multilevel cosine regression analysis used as the main analysis in research question 1 was designed to detect both premenstrual and mid-cycle symptom increases using the positive (W-shape) / negative (M-shape) cosine amplitudes. A proportional time variable was however used, anchoring the cycle at the onset of menses in cycle 1 and 2, meaning that the premenstrual increases of symptoms are aligned. Nevertheless, to address the concern of possible misalignment of the periovulatory phase, we have re-estimated the cosine analysis scaling the time variable consistently with the approach of PACTS, which has been more thoroughly described in the answer to question 6 by reviewer 1. Ovulation was set to day -15 as suggested in Nagpal et al. [5] when biological assessment of ovulation is not available. The new estimated cosine amplitudes were highly consistent across the cosine regression models estimated with the PACTS-like time variable and the proportional time variable used in the original submission (Figure RL9; RL stands for Response Letter), both for the patients with PMDD and controls, and the cosine amplitudes between the models were highly correlated on the individual level. This indicates that the results of the cosine regressions were robust to the method of cycle scaling, with amplitude estimates being virtually identical between cycles with separate follicular and luteal phase scaling with ovulation fixed at day -15 vs. cycles that were scaled uniformly. Nevertheless, the cosine regression models estimated with the proportional time variable presented in the original manuscript have been replaced with the new cosine regression models estimated with the PACTS time variable, and the original moved to the Supplementary materials.

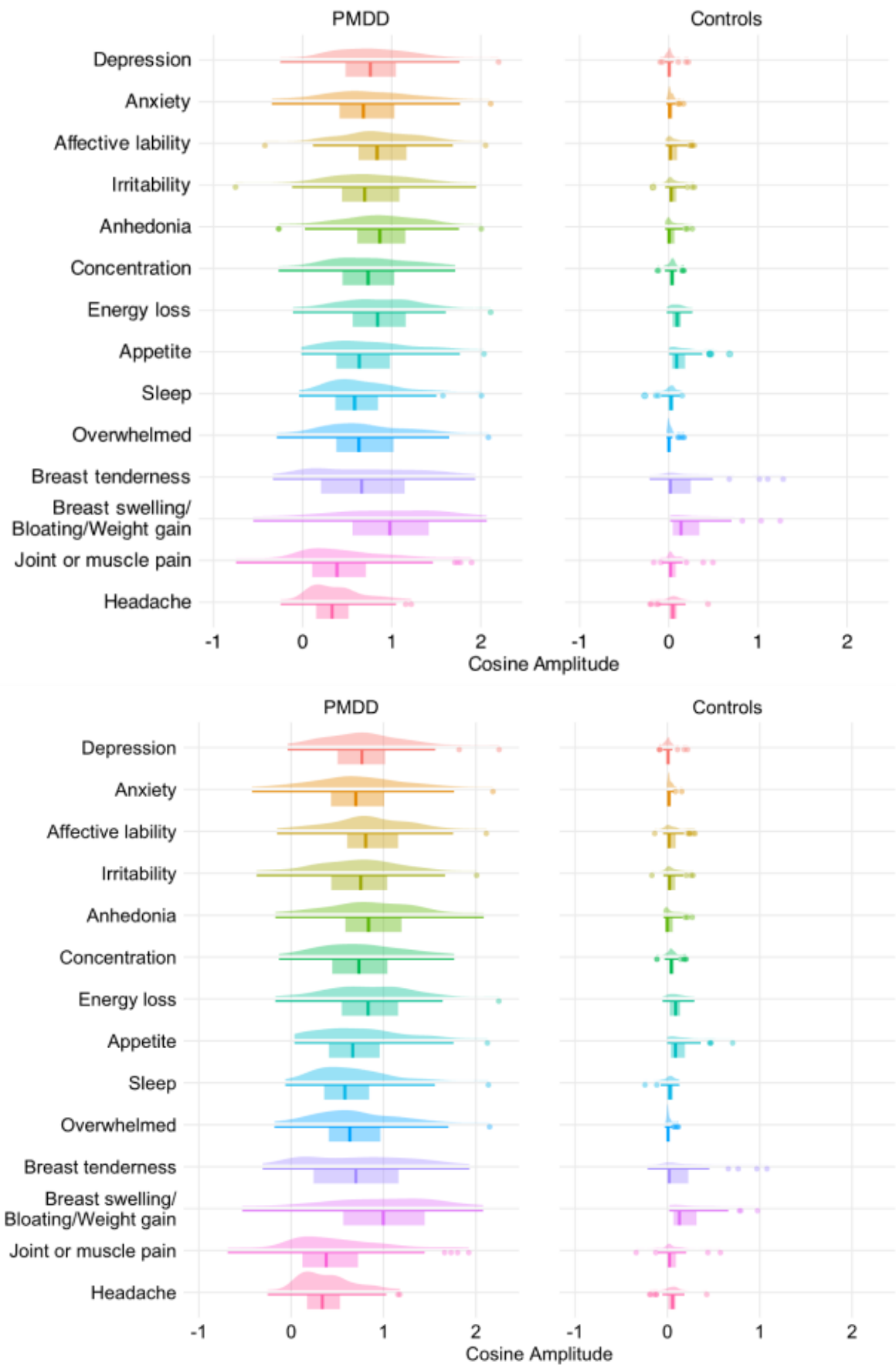

Figure RL9. Distribution of cosine amplitudes, illustrating the degree of convergence between the revised vs. the original statistical approaches.
Top: New analysis, Distribution of cosine amplitudes with PACTS scaling. The figure shows the distribution of the individual cosine amplitudes from the separate cosine regression models for each PMDD symptom for patients and controls, when estimating the models based on a PACTS scaled time variable, setting ovulation to day -15. The size of the cosine amplitude reflects the severity of the specific symptom, and the sign of the cosine amplitude reflects the timing of the symptom. **Bottom: Original analysis, Distribution of cosine amplitudes from original cosine regression models.** The figure shows the original figure of the distribution of cosine amplitudes from the cosine regression models estimated with time as a proportional variable computed based on the onset of menses and cycle length. The size of the cosine amplitude reflects the severity of the specific symptom, and the sign of the cosine amplitude reflects the timing of the symptom.

The LCGA analysis used as the main analysis in research question 2 was restricted to the symptomatic window of day -14 to day +9, where day 0 is the onset of menses, and is therefore not affected by this limitation. This approach is also consistent with the one used in Eisenlohr-Moul et al. [6].

Finally, the CT-VAR analysis used as the main analysis in research question 3 is unlikely to have been affected by the potential ovulation misalignment. This analysis models temporal dynamics between symptoms by capturing deviations from each individual's baseline (the low/no symptom state in the follicular phase). Because the model tracks day-to-day symptom changes relative to this baseline, it will detect premenstrual symptom elevation when it occurs and is therefore not dependent on precise ovulation or cycle phase alignment.

We now explicitly acknowledge in the Discussion of each of the three main analyses, how the potential misalignment of the periovulatory phase and ovulation likely had a minor or no impact on the results, as the main interest was on premenstrual dynamics. The discussion regarding the cosine regression results on page 10 now reads:

"In addition to the W-shaped pattern, the cosine approach also allowed detection of mid-cycle M-shaped symptom increases, which reflect periovulatory symptom changes. Ovulation was not biologically confirmed in the present study. Cosine regression models were however estimated using a phase-aligned cycle time scaled (PACTS) variable assuming ovulation at day -15 across individuals [23-25]. As the vast majority of cosine amplitudes were positive for all symptoms in patients with PMDD (94-100%; Figure 2), reflecting predominant W-shaped patterns, potential misalignment around the periovulatory phase likely had a minor, noise-like, impact on the results." **(Lines: 274-282)**

Further, the discussion regarding the results of the LCGA analyses on page 11 now reads:

"As the focus of this analysis was on premenstrual dynamics and the luteal phase is assumed to be relatively stable [24, 25], the results are considered less sensitive to ovulation timing. Furthermore, these results were consistent with those found in an independent sample for total DRSP scores [21]." **(Lines: 296-299)**

And finally, the discussion regarding the CT-VAR results on page 13 now reads:

"CT-VAR modelling, capturing deviations during the premenstrual phase from each individual's low/no-symptom state in the follicular phase, revealed temporally ordered associations among the core PMDD symptoms across adjacent observations, whereby the worsening of one symptom was associated with subsequent worsening of other symptoms (Figure 6). As CT-VAR models within-person symptom dynamics rather than precise cycle transitions, these findings are likely less sensitive to ovulation misalignments (Figure 6)." **(Lines: 364-369)**

2. • On that note, the follicular phase is highly variable, and they define it as days 6-12 for PMDD determination > were differences in length of cycle taken into consideration?

Authors' response: We thank the reviewer for pointing out the lack of clarity regarding menstrual cycle data use. The variable follicular phase length did not affect the results (cosine regressions) or was not relevant (LCGA and CT-VAR), as described above. We also want to clarify that the day 6-12 range for the follicular phase was only used in the inclusion of participants with PMDD, to evaluate the increase in symptoms between the follicular and luteal phase. Days 6-12 were assumed to be asymptomatic, which should apply for all cycle lengths of at least 24 days (which was the minimum cycle length in the PMDD group of this sample). Days -7 to -1 used as the luteal phase in the inclusion of participants with PMDD was assessed through backward counting from the onset of menses verified using bleeding records. The only impact of using days 6-12 of the follicular phase in the determination of PMDD would be to potentially miss participants with short cycle lengths (<24 days). However, there were no participants with PMDD with cycle lengths below 24 days.

We now clarify the inclusion of PMDD in the Methods section on page 16, and acknowledge in the Discussion of the results of each research question on page 10, 11, and 13 (as indicated in the response to the reviewer's previous question), that not accounting for cycle length variability may introduce some misalignment around the periovulatory phase, but likely only had very minor impact on both inclusion of participants with PMDD and the results. It did not have any impact on the results of the LCGA analysis as the primary focus was the symptomatic phase (research question 2), or the CT-VAR analysis as the focus was temporal dynamics between PMDD symptoms (research question 3). For the cosine regressions (research question 1), in which we included both cycles, we did account for differences in cycle length by using a proportional time variable. However, ovulation might still have been misaligned as it does not occur mid-cycle for shorter and longer cycle lengths. To account for this, we re-estimated the cosine regression models using a time variable scaled differently in the follicular and luteal phase (PACTS), with ovulation set to day -15 across subjects, as suggested in Nagpal et al. [5] when biological measures of ovulation is not available. This yielded virtually identical results as in the original analysis, confirming that potential misalignment of ovulation did not affect our results (Figure RL9).

3. • Age range is 20-46 and there are changes in cycle structure across that timespan so was age considered in the analyses. Given this, it might be wise to do a sensitivity analyses? In addition, no mention is made of history of contraceptives or pregnancy which could also impact cycles. Was any history taken? Did it differ among groups. This is more out of curiousity.

Authors' response: We thank the reviewer for this comment. We conducted analyses considering age for each of the three main analyses and show that the main findings remained virtually similar.

This is reported in the Results section on page 6:

"likewise age-adjusted analyses (Supplementary Note 1; Supplementary Figure 3-5; Supplementary Table 6-7)" **(Lines: 145-146)**

On page 7:

"Age-adjusted analyses yielded similar results (Supplementary Note 1; Supplementary Figure 9; Supplementary Table 11)." **(Lines: 170-171)**

And finally on page 9:

"likewise age-adjusted analyses (Supplementary Note 1; Supplementary Figure 10; Supplementary Table 12)" **(Lines: 238-239)**

Finally, the details of the sensitivity analysis was added in the Supplementary materials in the Supplementary Note 1 on page 11-13 **(Lines: 329-389)**, Figure RL10 was added as Supplementary Figure 8, Figure RL11 as Supplementary Figure 9, Figure RL12 as Supplementary Figure 10, Table RL5 as Supplementary Table 11, and Table RL6 as Supplementary Table 12.

In detail:

Cosine regression analyses
For the cosine regression analyses, we initially tested if the output from the original cosine regression models correlated with age by running Spearman correlations between the individual cosine amplitudes of each symptom from the original models and age, both for patients with PMDD and controls. No cosine amplitudes of any symptom except "Joint or muscle pain" for controls correlated significantly with age (ρ=-0.38, p=0.01), indicating that older controls have less cyclical variation for joint or muscle pain across the menstrual cycle. This may reflect age-related increases in baseline pain levels, resulting in less pronounced cyclical variation. Cosine amplitudes of all other symptoms did not correlate with age and therefore indicated that the cyclical variation did not differ across age.

As suggested by the reviewer, we also ran all cosine regression models again adjusting for age as a sensitivity analysis showing that the results were very robust. Age was a significant covariate only for "Irritability" in the PMDD group (p=0.004), with older patients with PMDD showing slightly higher levels of irritability, however the effect size was negligible (Estimate=0.013, cosine amplitude range for irritability=-0.38 to 2.02). Figure RL10 shows the distribution of the cosine amplitudes from each cosine regression model adjusted for age. Comparing Figure RL10 with the bottom of Figure RL9, that shows the distribution of the cosine amplitudes of the original models included in the manuscript, the distributions are nearly identical, confirming that the results of the cosine regressions are robust to age adjustment.

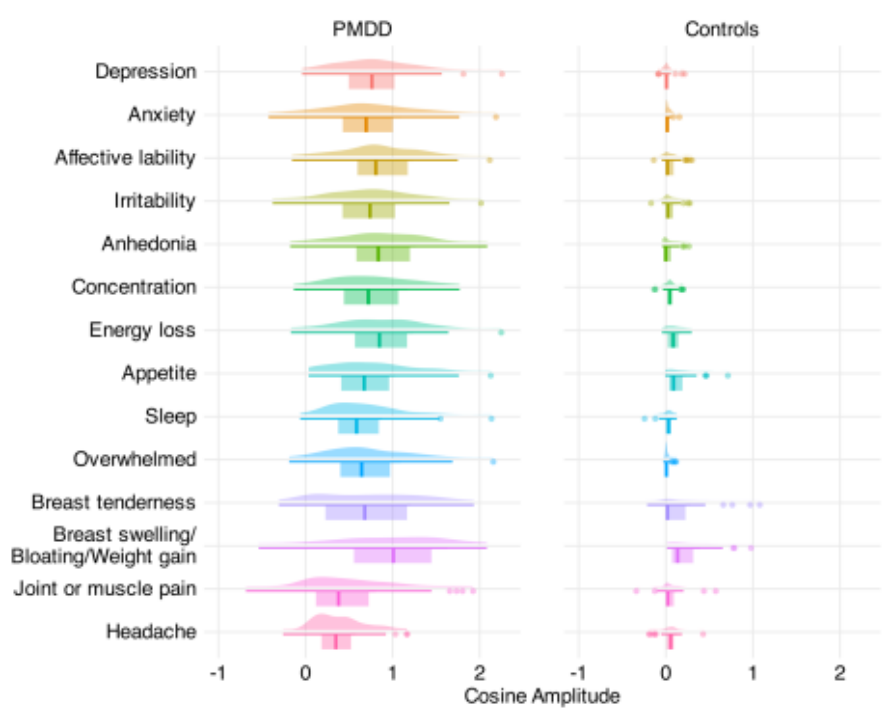

Figure RL10. Distribution of cosine amplitudes adjusting for age. The figure shows the distribution of the individual cosine amplitudes from the separate cosine regression models for each PMDD symptom for patients

and controls, when adjusting for age in the models. The size of the cosine amplitude reflects the severity of the specific symptom, and the sign of the cosine amplitude reflects the timing of the symptom.

LCGA analyses

For the LCGA analyses, we initially conducted Kruskal Wallis analyses for age across the three classes for each LCGA model, to test if age differed significantly between the original classes. No significant differences were found between the classes in each symptom and the total DRSP score. We further conducted sensitivity analyses by including age as a covariate in all LCGA models, to see if the shapes of the temporal patterns changed when adjusting for age, and if class membership differed between unadjusted and adjusted models. One patient was missing information on age, and therefore these comparisons include 89 patients instead of 90. Table RL5 shows that class assignments were highly consistent between original and age-adjusted models across most symptoms, with 14 out of 15 models exceeding 90% agreement (or close to as in the case of energy loss with 86.5% agreement). However, concentration difficulty showed lower agreement (58.4%), suggesting that age may have had a greater influence on temporal pattern solutions for this symptom specifically. Shapes of temporal patterns were virtually identical for all classes in 14 out of 15 models (comparing Figure RL11 with Figure 4 of the original manuscript), except for ‘concentration difficulty’ in which the age-adjusted model found a temporal pattern with a later offset of symptoms that was not found in the original solution (although including only 9% of participants).

Table RL5. Agreement of participants in the same LCGA class between the original model and the age-adjusted model. The table includes how many participants that belonged to the same class in the original LCGA models for each symptom and total DRSP score as after adjusting for age vs. belonged to a new class after adjustment.

Symptom	N same class	N changed class	% Agreement between models
Total DRSP score	88	1	98.8%
Depression	88	1	98.8%
Anxiety	87	2	97.8%
Affective lability	88	1	98.8%
Irritability	83	6	93.3%
Anhedonia	86	3	96.6%
Concentration	51	38	58.4%
Energy loss	77	12	86.5%
Appetite	81	8	91.0%
Sleep	84	5	94.3%
Overwhelmed	87	2	97.8%
Breast tenderness	89	0	100%
Breast swelling/Bloat/Weight gain	86	3	96.6%
Joint or muscle pain	85	4	95.5%
Headache	86	3	96.6%

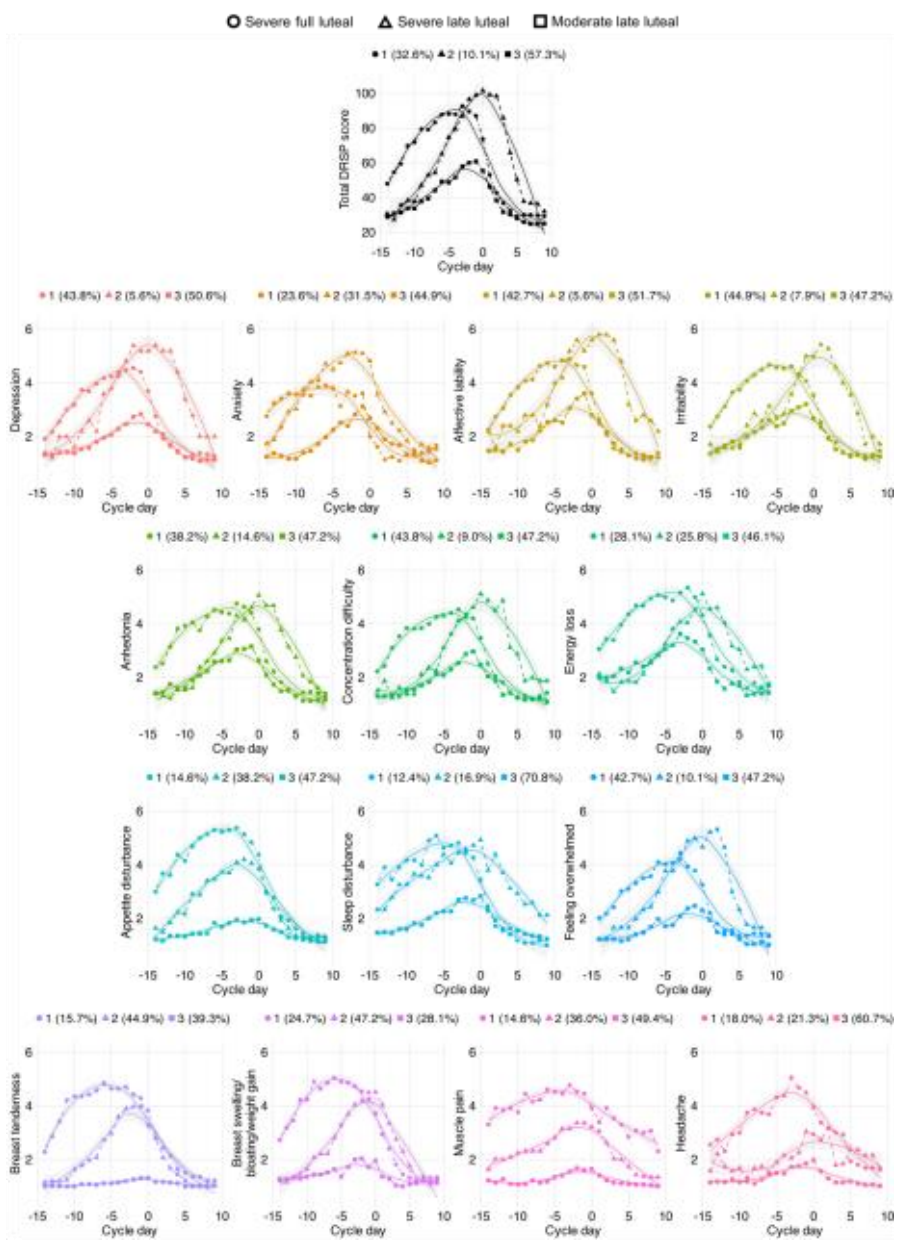

Figure RL11. Temporal patterns of premenstrual symptoms (age adjusted). The graphs show the result of the LCGA models adjusted for age with the k=3 solution for the total DRSP score and each individual symptom.

CT-VAR analyses

For the CT-VAR analyses, we included scaled age as a time-independent predictor and re-estimated the model with the four core symptoms of PMDD. From this model we could extract drift parameters estimated when keeping age constant at the mean (~35 years) (Figure RL12). Comparing Figure RL12 with Figure 6 of the revised manuscript, showing the network of the original CT-VAR analysis, it can be seen that they follow the key same patterns.

As in the original results, Depression went faster back to a low/no-symptom state than the other three core symptoms (more negative drift parameter estimate), and it had a dampening effect on the other symptoms, anxiety was a driver of the rate of change of all the other core symptoms with similar positive cross-symptom effects as in the original model. One difference noted between the age-adjusted model and the original model was that when adjusting for age, affective lability had a significant cross-symptom effect on the rate of change of depression and irritability, and irritability similarly had a significant cross-symptom effect on the rate of change of affective lability. So, affective lability was slightly more involved in the dynamic process compared to the original unadjusted model. Table RL6 of this letter further includes significant moderations of age, which should be interpreted as effects were age was a significant moderator of the effect. The positive effect of both anxiety and irritability on the rate of change of depression was estimated to be larger than the average estimate for older participants. On the contrary, the positive effect of irritability on the rate of change of affective lability was estimated to be smaller than the average estimate for older participants. Finally, the rate of which affective lability and irritability returns to equilibrium was found to be slower for older participants.

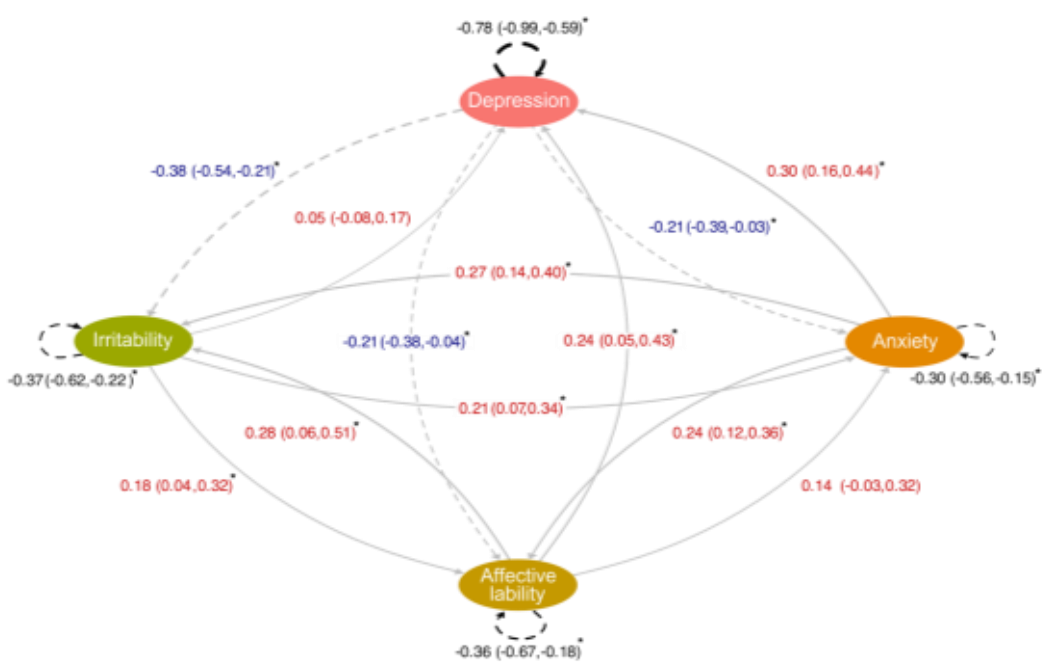

Figure RL12. Dynamic network of the core symptoms (age-adjusted). Dynamic network based on CT-VAR model adjusted for age.

Table RL6. Effect of age on drift parameters estimated in the age-adjusted CT-VAR model.

**Symptom 1 to Symptom 2**	**Age moderation estimate**	**95% CI**	**Interpretation**
Anxiety to Depression	0.12	[0.02, 0.23]	The significant positive effect of anxiety on the rate of change of depression is larger for older participants
Affective lability to Affective lability	0.10	[0.04, 0.18]	The rate of which affective lability returns to equilibrium is slower for older participants
Irritability to Depression	0.16	[0.03, 0.29]	The effect of irritability on the rate of change of depression is larger for older participants
Irritability to Affective lability	-0.17	[-0.31, -0.06]	The effect of irritability on the rate of change of affective lability is smaller for older participants
Irritability to Irritability	0.13	[0.07, 0.22]	The rate of which irritability returns to equilibrium is slower for older participants

Other
Regarding the second point of this comment, we did not assess history of contraceptives, except for if individuals in the PMDD groups had been using hormonal contraceptives as a treatment for PMDD in the past. This is reported in Supplementary Table 1 on page 14-15 in the Supplementary materials. Regarding history of pregnancies, we did ask this in the screening process, and it is included in Supplementary Table 1. It did differ significantly between groups, but as we did not have a direct comparison between groups, it was not considered in the analyses.

Regarding stats:
4. • The use of the scaled cyclepoint variable is appreciated. And while it makes good sense to use a cosine regression for cyclical effects, were alternative models/functions considered. Please take a look at this paper and the use of GAM(M) modeling which may be a better approach as it is data-driven and works well with standardized cycle variables (Perovic et al. 2023 Scientific Reports 13, 21999 https://www.nature.com/articles/s41598-023-48628-x)

Authors' response: We thank the reviewer for this comment. The goal of research question 1 was to test for variability across individuals and symptoms using a statistical approach that provides information that is not provided by other approaches. Specifically, using cosine regressions provides coefficients indicating the direction and magnitude of cyclical effects, distributional information of cyclical change (mean level, variance), and that can be used to test for associations in cyclical changes across variables/symptoms (e.g., are cyclical changes in mood associated with cyclical changes in somatic symptoms?). We used cosine regression to approach this specific aim since it is an established method to model cyclical patterns in time-series data by fitting a sinusoidal wave, particularly suited for menstrual cycle research as it can estimate the cyclical variation for each individual, as described in [7].

A central benefit of the cosine approach is the cosine coefficients that are generated for each individual. These can directly give us the direction and magnitude of symptom change for each individual, which can be useful to explore heterogeneity of PMDD symptoms within and across patients with PMDD and controls, as well as associations among cyclical changes across variables. Notably, GAMM models that are used in Perovic et al. [10], do not directly give any equivalent parameter estimates that provide this type of information or analytic opportunities. The cosine function may not fully capture the heterogeneity in symptom trajectory shape as not all individuals do fit into a smooth sinusoidal "M" or "W" pattern, which GAMM may do better since it is more flexible. However, the LCGA analyses presented in research question 2 directly address this limitation by allowing flexible, data-driven identification of distinct trajectory shapes across individuals. Unlike GAMM, which is a suitable model to fit data in a more flexible manner than cosine regression and can be used to find the average shape of symptom expression, LCGA identifies latent subgroups of individuals following similar

trajectory patterns, providing both the flexibility of GAMM and the additional benefit of characterizing heterogeneity across individuals through class membership. We do not see the decision between the use of cosine-regression vs GAMM as an either-or decision, but rather, two approaches which provide different types of information that could both be considered to gain the richest understanding possible. Since LCGA was already included as the next analysis, we would argue that the combination of using cosine regression and LCGA, provides the same information as would have been provided by GAMM and more.

Nevertheless, upon the reviewer's suggestion, we have estimated GAMM models as a complementary analysis to the cosine regression, and to see how well the average curve for each symptom fits the data. Figure RL4 shows the GAMM-estimated population average symptom trajectories together with the average cosine regression curves for each symptom across the two menstrual cycles for PMDD and controls, respectively. The dashed lines represent the GAMM population average smooth, the solid lines with dots represent the average cosine regression curve, and the shaded area represents ±1SD of raw DRSP scores, reflecting individual variability around the average trajectory. We clearly see the W-shape of the average symptom trajectory estimation of the GAMM models for all symptoms in the PMDD group, which would suggest that cosine regression provides a valid estimate of the shape of change across time, comparable to the ones of GAMM, in addition to the estimates of the cosine coefficient. In Table RL7, the total explained variances of the models are presented, and as for the cosine regression models, we see that there is substantial variability that cannot be explained by the average symptom trajectories across two menstrual cycles. Thus, we believe that the Cosine regressions are a valid and meaningful way to address research question 1, with the addition of LCGA to address research question 2 to investigate the substantial heterogeneity in trajectory shape – found both with cosine regression and GAMM models.

Table RL7. Total explained variance of GAMM models for each symptoms for patients with PMDD and controls.

	R^2 adjusted	
**Symptom**	**PMDD**	**Controls**
Depression	0.27	0.04
Anxiety	0.25	0.04
Affective lability	0.29	0.06
Irritability	0.27	0.03
Anhedonia	0.26	0.007
Concentration	0.22	0.02
Energy loss	0.21	0.02
Appetite	0.22	0.02
Sleep	0.18	0.003
Overwhelmed	0.22	0.04
Breast tenderness	0.24	0.03
Breast swelling/ Bloating/Weight gain	0.29	0.07
Joint or muscle pain	0.09	0.01
Headache	0.07	0.04

However, to strengthen the transparency, the complementary GAMM analyses have been added to the supplementary material in the revised manuscript. Figure RL4 was added as Supplementary Figure 5 on page 33 of the Supplementary materials, and the following text was added to the Supplementary materials on page 10 under Supplementary analyses:

"Additionally, GAMM models were estimated using a proportional time variable scaled uniformly across each cycle, ranging from 0 to 2. The average pattern across the two cycles was similar to the average W-shape that was found with cosine regression estimated with the same time variable (Supplementary Figure 5), which suggests that cosine regression provides valid estimates of the shape of change across time, comparable to the ones of GAMM." **(Lines: 299-303)**

In the Methods section of the revised manuscript, the following addition was made on page 18:

"Lastly, as a complement to the cosine regression models, Generalized Additive Mixed Models (GAMM) were estimated to examine the explained variability and the shapes of the observed average patterns of change across symptoms (Supplementary Note 1)." **(Lines: 510-512)**

In the Results section of the revised manuscript, the following addition was made on page 6:

"Complementary Generalized Additive Mixed Models (GAMM), using both PACTS-scaled and uniformly scaled proportional time variables, captured the average observed pattern of symptom change across the menstrual cycle and supported the robustness of the cosine regression findings (Supplementary Note 1; Supplementary Figure 3-5; Supplementary Table 6-7)." **(Lines: 149-153)**

5. • It would be good to include the number of people in each of the clusters they identified in Table S5, where there any demographic differences between people in clusters? Similarly please give us % in lines 183-189 – not "some" "others"

<u>Authors' response:</u> The authors thank the reviewer for this comment. Number of people in each cluster identified for k=1:4 has been added to Supplementary Table 8 in the revised manuscript (which corresponds to Table S5 in the original manuscript). The original column "Temporal pattern percentages (%)" has been renamed to "Temporal pattern size (N (%))" and now includes both number of people and the percentage out of the total number of people. Supplementary Table 8 is however for model selection, in which BIC, AIC, and temporal pattern percentage was considered, but we agree that it is more transparent to also present the number of people in each cluster.

To further address the reviewer's question about demographic differences between people in clusters, we compared all demographic characteristics presented in Supplementary Table 1 in the manuscript between clusters across all symptoms and total DRSP score. This was done using Kruskal-Wallis tests for continuous demographic variables and Fisher's exact tests for categorical demographic variables. Although a few nominal differences were observed before correction for multiple testing, none remained statistically significant after FDR correction, suggesting that temporal pattern membership was not related to differences in demographic characteristics.

Regarding the reviewer's request for percentages in the original manuscript lines 183-189, we have revised these sentences and replaced qualitative descriptors such as "some" and "others" with percentages reflecting the proportion following the same trajectory for all symptoms in each symptom cluster found with hierarchical clustering, and the proportion following the same trajectory for all symptoms. In the revised manuscript on lines 194-205 (corresponding to the paragraph on lines 183-189 in the original manuscript) on page 8 it now reads:

"Clusters based on participants' patterns of temporal changes across symptoms and on individual symptoms' patterns of change were identified through two-dimensional hierarchical cluster analysis (Figure 5). Patients generally showed similar patterns of change across diverse symptoms. However, this similarity appeared to be strongest for the psychological and vegetative symptoms, where 39% and 23% followed the same temporal pattern across all symptoms, respectively. Regarding physical symptoms, the proportion following the same temporal pattern was 21%. However headaches and joint or muscle pain emerged as separate clusters in the hierarchical clustering, and 68% followed the same temporal pattern for breast tenderness and breast swelling/bloating/weight gain. The majority of patients (98%) displayed a variable symptom profile, with different symptoms or symptom clusters flowing different temporal patterns; all symptoms followed the same temporal pattern in only 2% of patients." **(Lines: 194-205)**

6. • Instead of "moment-to-moment dynamics" wouldn't daily dynamics be appropriate?

<u>Authors' response:</u> Thank you for this comment. We agree and have now replaced "moment-to-moment dynamics" with "daily dynamics" throughout the text.

7. • P values or effect sizes in the results would be welcome.

Authors' response: We thank the reviewer for this comment. We agree that reports of p-values and/or effect sizes would increase transparency of the results. For the main cosine regression analysis with the PACTS scaled time variable, significance of fixed and random effects, Bonferroni corrected, is indicated with significance stars in Supplementary Table 3; ***: p<0.001; **: p<0.01; *: p<0.05. The LCGA analyses are based on model selection criteria such as BIC, AIC, and temporal pattern proportions, and p-values are therefore not applicable, but rather the model selection parameters, average posterior probabilities, and entropy (which is a measure of class separation). The range of average posterior probabilities was indicated in the Result section on page 6 in the original submission. Lastly, for the CT-VAR models, significance is reported with significance stars in Figure 6 of the manuscript, together with the drift parameters and confidence intervals for each cross-symptom and autoregressive effect.

To address the reviewer's suggestion, we have now reported p-values and effect sizes throughout the Results section when applicable. For the results of the LCGA we put more emphasis on the average posterior probabilities in the text, and added the range of entropy values to further strengthen the class separation. Entropy values for each model have also been added to Supplementary Table 8 on page 23-25 in the Supplementary materials.

8. • Was cycle phase accounted for in the CT-VAR models? IF this is an average ("all days from the most severe cycle for each patient with PMDD was used") this may be potentially underestimating the symptom relationships during the high-symptom parts of the cycle and overestimating during the low-symptom parts (especially if more severe cycles are selected, so the differences between no-symptom and symptom parts of cycle are larger).

Authors' response: We apologize for the lack of clarity regarding the CT-VAR models. The statement included in the manuscript that the reviewer is mentioning "all days from the most severe cycle for each patient with PMDD was used", was referring to that all days were used in the model, not the average across days which would not have been possible with the CT-VAR approach, as it requires several timepoints per subject to be able to estimate the temporal dynamics between symptoms. This has now been clarified in the Figure text of Figure 6 and 7 on page 31 and 32 of the revised manuscript, as well as in the Supplementary Methods section on page 7 of the Supplementary materials.

9. • Table S3 is interesting given the larger discrepancy between certain symptoms in PMDD and controls. Of course some are expected given the population but it is worth noting these for those not as familiar with PMDD (which will be a lot of people). Many of the somatic symptoms are seen in both populations but some of the others symptoms do have a perhaps notable difference (headaches, sleep, overwhelmed) which is imp to note in the discussion at least.

Authors' response: We appreciate the reviewer's comment. The authors think that Table S3 in the original manuscript might have been somewhat misleading, as it just presents the proportions of positive cosine amplitudes without considering magnitude of the amplitude. For this reason, we have decided to remove the table, as Figure 2 in the original manuscript already visualizes the large proportion of positive cosine amplitudes while also capturing the magnitude of the values. Nevertheless, we agree with the reviewer that it is interesting that controls show larger positive cosine amplitudes for some symptoms (such as appetite, breast tenderness, and breast swelling/bloating/weight gain), while other symptoms are not experienced to the same extent by controls, or more commonly experienced mid-cycle (such as sleep, joint or muscle pain, and headache). This have now been added to the Discussion section on page 10-11:

"In comparison, controls showed mild premenstrual increases of appetite, breast tenderness and breast swelling/bloating/weight gain, for which the discrepancies in variance explained by the menstrual cycle between patients with PMDD and controls were less prominent than for other symptoms such as sleep and energy loss. Mild mid-cycle symptom increases were more common for sleep, joint or muscle pain, and headache in controls." **(Lines: 286-291)**

10. • IN addition to the point above, maybe it is my mistake but it appears that other symptoms in S4 show great disparities between variance accounted for in PMDD and controls that are worth mentioning: joint pain, overwhelmed, sleep, energy, maybe even appetite. But these are not mentioned in the discussion or results when talking about the data in Table S4.

Authors' response: We thank the reviewer for pointing out this discrepancy that should be mentioned in the discussion. We have integrated this with the addition of the reviewer's previous point to the Discussion section on page 10-11:

"In comparison, controls showed mild premenstrual increases of appetite, breast tenderness and breast swelling/bloating/weight gain, for which the discrepancies in variance explained by the menstrual cycle between patients with PMDD and controls were less prominent than for other symptoms such as sleep and energy loss. Mild mid-cycle symptom increases were more common for sleep, joint or muscle pain, and headache in controls." **(Lines: 286-291)**

11. • In addition to the core symptoms of PMDD and using them for drivers of change in symptoms.. I am curious were other symptoms tested? Given that there are large changes for sleep and energy maybe these symptoms are also drivers along with anxiety? Or alone? Lines 204-216. Similar question for Figure 7 – why only the core symptoms?

Authors' response: We would like to thank the reviewer for raising this point. As mentioned on row 215-219 in the original manuscript, an additional CT-VAR model was estimated including anxiety, anhedonia, concentration, and feeling overwhelmed (secondary psychological symptoms), where similar effect sizes of significant positive cross-symptom effects from anxiety to anhedonia and concentration difficulties were observed. The figure of the dynamic network including the estimated cross-symptom and autoregressive effects has been included in this letter (top of Figure RL13). To address the reviewer's comment, we further estimated an additional CT-VAR model as an extra analysis, including anxiety, sleep, energy loss, and appetite (bottom of Figure RL13). Anxiety showed positive cross-lagged effects on all three symptoms. Sleep and energy loss did however not increase the rate of change of anxiety significantly. Figure RL13 was added as Supplementary Figure 6 in the Supplementary materials on page 34.

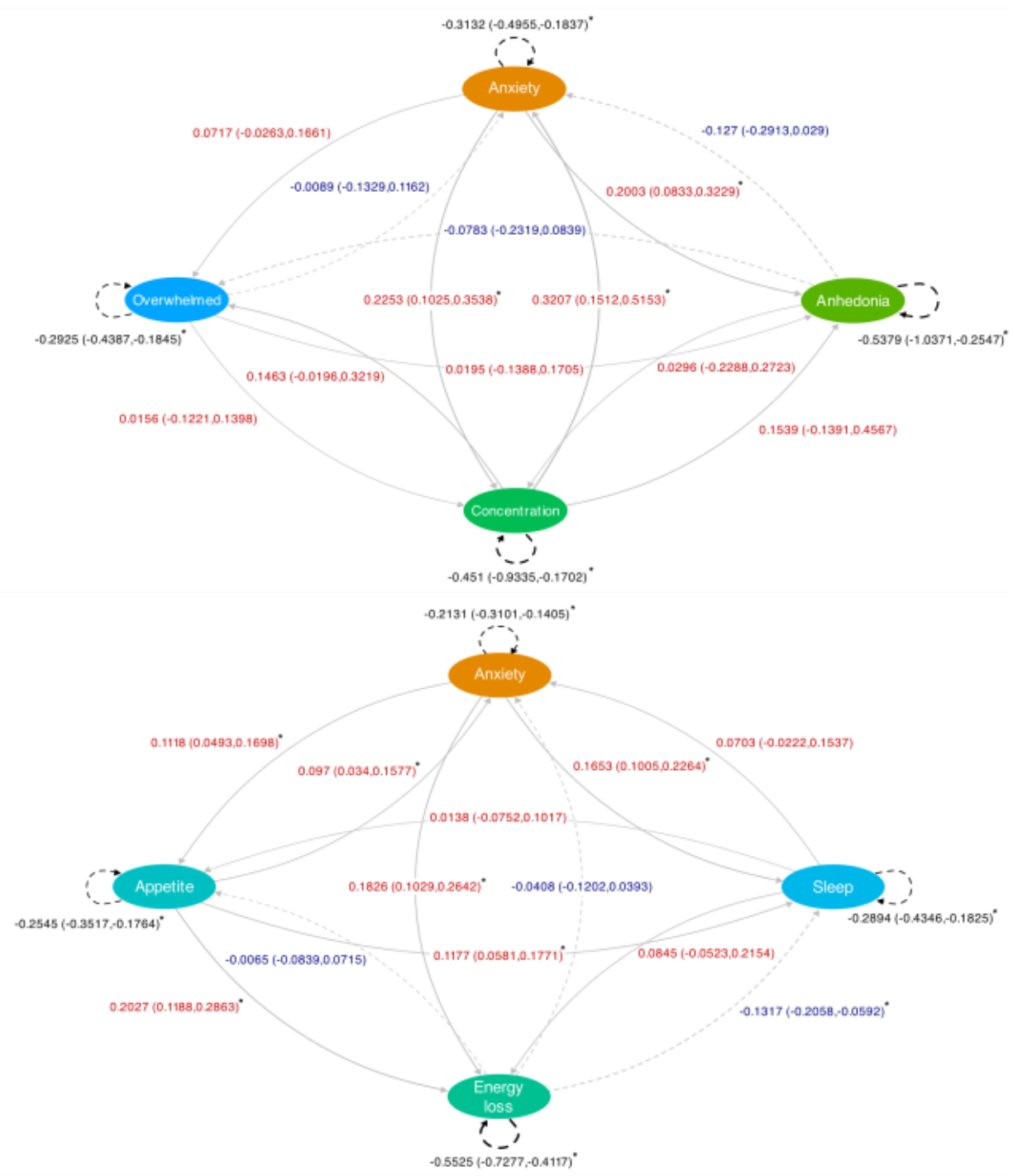

Figure RL13. Dynamic networks of additional CT-VAR models. The top network shows the cross-symptom and autoregressive effects of the CT-VAR model including anxiety together with the secondary psychological symptoms of PMDD; anhedonia, concentration, and feeling overwhelmed. The bottom network shows the cross-symptom and autoregressive effects of the CT-VAR model including anxiety together with the vegetative symptoms of PMDD; appetite, sleep, and energy loss. * Indicate effects that were significant after correction for multiple testing using the Benjamini-Hochberg correction. See Figure 6 for a full description of model parameters and interpretation.

The following paragraph was added to the Supplementary analyses section of the Supplementary materials on page 11:

"To further examine whether the associations of anxiety extended beyond the core symptoms of PMDD, two complementary CT-VAR models were estimated. The first model included anxiety and the three secondary psychological symptoms included in the PMDD diagnosis (anhedonia, concentration difficulties, and feeling overwhelmed), and the second model included anxiety and the three vegetative symptoms included in the PMDD diagnosis (energy loss, appetite, and sleep). Anxiety was significantly associated with increases in the rate of change of anhedonia, concentration difficulties, and all vegetative symptoms (Supplementary Figure 6), further supporting the central role of anxiety in the temporal dynamics of PMDD symptoms." **(Lines: 320-327)**

To the Results section on page 8, the following was added:

"Complementary analyses with anxiety and the secondary psychological symptoms (anhedonia, concentration difficulties, and feeling overwhelmed) and anxiety with the vegetative symptoms (energy loss, appetite, and sleep), showed that anxiety also was associated with the rate of change of anhedonia, concentration difficulties, and all vegetative symptoms with the same direction and similar effect sizes (Supplementary Figure 6)." **(Lines: 225-229)**

And finally, in the Discussion section on page 13 where the first extra model was already mentioned in the original manuscript, now reads:

"Results based on the cross-symptom effects identified anxiety as a potential dynamic driver of the other symptoms' rate of change, suggesting that increases in anxiety were associated with accelerated worsening of the other three core symptoms, as well as five of the secondary psychological symptoms." **(Lines: 369-372)**

To further address the reviewer's question about why only the core symptoms were included. This restriction was due to three reasons: Firstly, these symptoms represent the primary defining features of PMDD as specified in the DSM-5 diagnostic criteria, and are therefore most clinically meaningful targets for understanding PMDD symptom dynamics. Secondly, including additional symptoms would substantially increase the number of parameters to be estimated, e.g., a 4-symptom model estimates 16 drift parameters, whereas including all 14 symptoms for instance would require estimation of 196 drift matrix parameters, exceeding what can be reliably supported given our sample size. Lastly, CT-VAR networks become increasingly difficult to interpret with increasing number of variables included, as the model represents an underlying dynamic system rather than one effect by two variables, meaning that for each additional variable the underlying dynamic system becomes more complex. This clarification has been added to the Methods section on page 19 in the revised manuscript which now reads:

"The model was limited to the core symptoms as these represent the primary defining features of PMDD, and including all symptoms would substantially increase the model complexity and number of parameters to be estimated, risking unreliable estimates given the sample size. A sensitivity analysis using the less symptomatic cycle was performed to assess the robustness of the findings, as well as two extra CT-VAR models including anxiety with the secondary psychological symptoms and the vegetative symptoms of PMDD, respectively (Supplementary Note 1)." **(Lines: 537-541)**

The first sentence was also added to the Supplementary Methods section on page 7 **(Lines: 199-202)** of the Supplementary materials.

12. • Although PMDD proportions varied lines 283-286, was it also not true that the % variance accounted for with random effects was higher in PMDD for these symptoms? Joint, headache? How do you reconcile those findings?

Authors' response: We thank the reviewer for this comment. Indeed, the % variance accounted for with random effects of the menstrual cycle was higher in PMDD for joint or muscle pain and headache compared to controls, reflecting greater inter-individual variability in the degree to which these symptoms fluctuate across the menstrual cycle for patients with PMDD. The proportions mentioned on lines 283-286 of the original manuscript refer to the most common temporal pattern (e.g. moderate late luteal pattern) for joint or muscle pain and headache found with the LCGA analysis, which were nearly asymptomatic. However, the LCGA also identified two additional symptomatic temporal patterns for both symptoms, including a substantial minority of the patients with PMDD. The co-existence of very mild/asymptomatic and symptomatic temporal patterns across patients is thus consistent with the higher % variance accounted for by random effects for both joint or muscle pain and headache (22% and 12%, respectively), which captures the variability around the average menstrual cycle effect across individuals.

Minor
13. Line 97-99.. sentence structure not quite right
Authors' response: We thank the reviewer for noticing this. We have now changed the sentence in the text on lines 100-103 on page 4 of the revised manuscript to:

"iii) How is the level of severity of one symptom associated with the rate of change of another symptom (or itself) and how does the level of severity of one symptom predict the severity of another symptom (or itself) at a later specific point in time (Figure 1)?" **(Lines: 94-97)**

REFERENCES

1. Halbreich, U., *et al.* Clinical diagnostic criteria for premenstrual syndrome and guidelines for their quantification for research studies. *Gynecol Endocrinol* **23**, 123–130 https://doi.org/10.1080/09513590601167969 (2007).
2. Crawford, N. M., Pritchard, D. A., Herring, A. H. & Steiner, A. Z. Prospective evaluation of luteal phase length and natural fertility. *Fertil Steril* **107**, 749–755 https://doi.org/10.1016/j.fertnstert.2016.11.022 (2017).
3. Lenton, E. A., Landgren, B. M. & Sexton, L. Normal variation in the length of the luteal phase of the menstrual cycle: identification of the short luteal phase. *Br J Obstet Gynaecol* **91**, 685–689 https://doi.org/10.1111/j.1471-0528.1984.tb04831.x (1984).
4. Fehring, R. J., Schneider, M. & Raviele, K. Variability in the phases of the menstrual cycle. *J Obstet Gynecol Neonatal Nurs* **35**, 376–384 https://doi.org/10.1111/j.1552-6909.2006.00051.x (2006).
5. Nagpal, A., *et al.* How to study the menstrual cycle as a continuous variable: Implementing phase-aligned cycle time scaling (PACTS) with the menstrualcycleR package. *Psychoneuroendocrinology* **181**, 107584 https://doi.org/10.1016/j.psyneuen.2025.107584 (2025).
6. Eisenlohr-Moul, T. A., *et al.* Are there temporal subtypes of premenstrual dysphoric disorder?: using group-based trajectory modeling to identify individual differences in symptom change. *Psychol Med* **50**, 964–972 https://doi.org/10.1017/s0033291719000849 (2020).
7. Kiesner, J., Mendle, J., Eisenlohr-Moul, T. A. & Pastore, M. Cyclical Symptom Change Across the Menstrual Cycle:Attributional, Affective, and Physical Symptoms. *Clinical Psychological Science* **4**, 882–894 https://doi.org/10.1177/2167702616635031 (2016).
8. Nagin, D. S. & Odgers, C. L. Group-based trajectory modeling in clinical research. *Annu Rev Clin Psychol* **6**, 109–138 https://doi.org/10.1146/annurev.clinpsy.121208.131413 (2010).
9. Sinha, P., Calfee, C. S. & Delucchi, K. L. Practitioner's Guide to Latent Class Analysis: Methodological Considerations and Common Pitfalls. *Crit Care Med* **49**, e63–e79 https://doi.org/10.1097/ccm.0000000000004710 (2021).
10. Perovic, M., Heffernan, E. M., Einstein, G. & Mack, M. L. Learning exceptions to category rules varies across the menstrual cycle. *Sci Rep* **13**, 21999 https://doi.org/10.1038/s41598-023-48628-x (2023).
